# Genome-wide identification and characterization of the NPF genes provide new insight into low nitrogen tolerance in *Setaria*

**DOI:** 10.3389/fpls.2022.1043832

**Published:** 2022-12-14

**Authors:** Jinjin Cheng, Helin Tan, Meng Shan, Mengmeng Duan, Ling Ye, Yulu Yang, Lu He, Huimin Shen, Zhirong Yang, Xingchun Wang

**Affiliations:** ^1^ College of Agriculture, Shanxi Agricultural University, Taigu, China; ^2^ State Key Laboratory of Crop Genetics and Germplasm Enhancement, Nanjing Agricultural University, Nanjing, China; ^3^ College of Life Sciences, Shanxi Agricultural University, Taigu, China; ^4^ Department of Basic Sciences, Shanxi Agricultural University, Taigu, China; ^5^ Shanxi Key Laboratory of Minor Crops Germplasm Innovation and Molecular Breeding, Shanxi Agricultural University, Taigu, China

**Keywords:** Setaria, nitrate/peptide transporter, expression profile, natural variation, three-dimensional structure, NRT1.1, low nitrogen tolerance

## Abstract

**Introduction:**

Nitrogen (N) is essential for plant growth and yield production and can be taken up from soil in the form of nitrate or peptides. The *NITRATE TRANSPORTER 1/PEPTIDE TRANSPORTER* family (*NPF*) genes play important roles in the uptake and transportation of these two forms of N.

**Methods:**

Bioinformatic analysis was used to identify and characterize the NPF genes in Setaria. RNA-seq was employed to analyze time-series low nitrate stress response of the SiNPF genes. Yeast and Arabidopsis mutant complementation were used to test the nitrate transport ability of SiNRT1.1B1 and SiNRT1.1B2.

**Results:**

We identified 92 and 88 putative *NPF* genes from foxtail millet (*Setaria italica* L.) and its wild ancestor green foxtail (*Setaria viridis* L.), respectively. These *NPF* genes were divided into eight groups according to their sequence characteristics and phylogenetic relationship, with similar intron-exon structure and motifs in the same subfamily. Twenty-six tandem duplication and 13 segmental duplication events promoted the expansion of *SiNPF* gene family. Interestingly, we found that the tandem duplication of the *SiNRT1.1B* gene might contribute to low nitrogen tolerance of foxtail millet. The gene expression atlas showed that the *SiNPFs* were divided into two major clusters, which were mainly expressed in root and the above ground tissues, respectively. Time series transcriptomic analysis further revealed the response of these *SiNPF* genes to short- and long- time low nitrate stress. To provide natural variation of gene information, we carried out a haplotype analysis of these *SiNPFs* and identified 2,924 SNPs and 400 InDels based on the re-sequence data of 398 foxtail millet accessions. We also predicted the three-dimensional structure of the 92 SiNPFs and found that the conserved proline 492 residues were not in the substrate binding pocket. The interactions of SiNPF proteins with ${\text{NO}}_{3}^{-}$ were analyzed using molecular docking and the pockets were then identified. We found that the SiNPFs NO_3_^−^ binding energy ranged from-3.4 to -2.1 kcal/mol.

**Discussion:**

Taken together, our study provides a comprehensive understanding of the *NPF* gene family in Setaria and will contribute to function dissection of these genes for crop breeding aimed at improving high nitrogen use efficiency.

## Introduction

Nitrogen (N) is an essential macronutrient for plant growth and crop production. Over the past half-century, global food production has increased considerably, which relies heavily on the application of N fertilizer (Liu et al., 2013; Li et al., 2017). It is estimated that over 120 Mt of N fertilizer is used annually and only approximately 30-40% of the applied N fertilizer can be used by crop plants (Raun and Johnson, 1999; Li et al., 2017). The excessive use of N fertilizers coupled with the low use efficiency of crops not only increases the cost of farmers but also causes severe environmental and ecological pollution (Guo et al., 2010; Xu et al., 2012). A better understanding of N uptake and transportation within the plant is crucial for molecular breeding for crops with high nitrogen use efficiency (NUE) and the development of sustainable agriculture (Xu et al., 2012).

In aerated soils, plants take up N mainly in the form of nitrate due to nitrification (Xu et al., 2012). To ensure an efficient up take over a wide range of external nitrate concentrations, plants have evolved two different nitrate transport systems, the low-affinity transport system (LATS) and the high-affinity transport system (HATS) (Edith Laugier et al., 2012). The LATS enables nitrate uptake in high (> 0.5 mM) external nitrate concentration, while the HATS allows nitrate absorption at low (< 0.5 mM) external nitrate concentrations (Crawford and Glass, 1998). The NITRATE TRANSPOTER 1 (NRT1)/PTR FAMILY (NPF) and NRT2 are responsible for LATS and HATS, respectively, except for AtNPF6.3 (also known as CHL1 or NRT1.1) that displays a dual affinity property (Liu et al., 1999). In addition to NPF and NRT2 genes, chloride channel (CLC) and slowly activating anion channel/homologues (SLAC1/SLAH) family genes also participate in nitrate transport (Krapp et al., 2014). Among these nitrate transporter families, NPF is the largest one and plays multifunctional roles in nitrate uptake and transport throughout the plant body (Wang et al., 2012).

During the last three decades, a great number of *NPF* genes were identified and extensively characterized in the model plants, especially in Arabidopsis and rice (Léran et al., 2014; Drechsler et al., 2018). Up to now, at least 53 *NPF* genes were identified in Arabidopsis, of which 19 were demonstrated to be involved in nitrate uptake and transport (**Supplementary Table 1**). The AtNRT1.1 and AtNRT1.2 transporters localized on the plasma membrane of the root epidermis, cortex or endothelial cell absorb nitrate from the outside (Tsay et al., 1993; Huang et al., 1999). *AtNRT1.1* was the first identified nitrate transport gene in Arabidopsis, which functions as a dual-affinity nitrate transport in the high and low affinity ranges (Tsay et al., 1993; Liu et al., 1999). The dual-affinity nitrate transport activity is achieved through the phosphorylation and dephosphorylation of threonine at position 101 (Parker and Newstead, 2014). This precise regulation mechanism enables plants to quickly switch between high-affinity and low-affinity systems to adapt to the varying levels of nitrate supply in the soil. While Nitrate Excretion Transport 1 (NAXT1), another member of the NRT1 family, can induce nitrate to flow out of the root under acidic conditions, thereby removing excess nitrate from the plants (Segonzac et al., 2007). The nitrate absorbed by the root system is further transported to the plant stem by AtNRT1.5, AtNRT1.8 and AtNRT1.9 (Lin et al., 2008; Li et al., 2010b; Wang and Tsay, 2011). In the later stages of plant growth and development, AtNRT1.7, AtNRT1.11 and AtNRT1.12 are responsible for the transport of nitrate from mature organs to young tissues and reproductive organs for reuse (Hsu and Tsay, 2013; Liu et al., 2017). The nitrate redistribution mediated by these transporters plays an important role in improving nitrogen use efficiency under low nitrogen conditions. In contrast to Arabidopsis, there are more NRT/PTR members in rice indicating that nitrate uptake and transport in rice are regulated more precisely. There are three putative homologs of *AtNRT1.1* were identified in rice: *OsNRT1.1A* (*OsNPF6.3*), *OsNRT1.1B* (*OsNPF6.5*), and *OsNRT1.1C* (*OsNPF6.4*) (Plett et al., 2010). Overexpression of *OsNRT1.1A* conferred high NUE, high yield and early maturation in rice, thus providing a target to produce high yield and early maturation simultaneously (Wang et al., 2018b). Another NRT1.1 gene, *OsNRT1.1B*, also plays an important role in nitrate utilization in rice. A single polymorphism in this gene contributes to the long-noted divergence in NUE between *indica* and *japonica* subspecies of Asian cultivated rice (Hu et al., 2015). In addition to the function of nitrate transporter and sensor, *O_S_NRT1.1B* is also involved in the establishment of the rice root microbiota and contributes to the divergence of the root microbiota between *indica* and *japonica* subspecies (Zhang et al., 2019). Recently, a rare variant of *OsNPF6.1*, *OsNPF6.1^HapB^* was identified that contributes to high NUE and can be trans-activated by OsNAC42, another NUE-related gene (Tang et al., 2019). In addition to the model plants, the NPF genes were also identified and characterized in several other species including sugarcane and coffee (Santos et al., 2017; Wang et al., 2019). These NPF genes provides valuable resources for high NUE and yield breeding.

The Setaria system contains two important C_4_ Panicoid grass species, namely wild green foxtail (*S Setaria viridis* L.) and its cultivated cousin foxtail millet (*Setaria italica* L.) (Kellogg, 2017). These two species demonstrate a wide capacity for adaptation. Green foxtail is one of the most widespread weeds on the planet; while foxtail millet is one of the most resilient cereal crops with high NUE that can grow on marginal land with minimal agricultural inputs. However, the molecular mechanisms underlying the barren tolerance in *Setaria* remain largely unexplored. Although a unified nomenclature of *NPF* genes in plants has been reported (Léran et al., 2014), this gene family has not been studied thoroughly in the barren-tolerant crop foxtail millet and its wild ancestor green foxtail. Recently, we developed a mini-plant (dubbed *xiaomi*) of foxtail millet with an extremely short life cycle of two months and produced a high-quality genome sequence by combining PacBio single molecule real-time sequencing chromosome conformation capture sequencing technology (http://sky.sxau.edu.cn/MDSi.htm) (Yang et al., 2020b). In this study, we carried out a BLAST search using Arabidopsis and rice NPFs against the newly available *xiaomi* genome and the A10 green foxtail genome (Bennetzen et al., 2012), and identified 92 and 88 *NPF* genes from *Xiaomi* and A10, respectively. We further performed a comprehensive analysis of these *NPF* genes including expression pattern, response to low nitrate, and natural variation and domestication. As the first systematic study of the *NPF* genes in foxtail millet, our results will provide a valuable information for selecting candidate genes to improve crop NUE and further investigating the function of NPFs in foxtail millet.

## Materials and methods

### Plant materials and low nitrate treatment

For low nitrate treatment, seeds of *xiaomi*, a fast life cycle mini-foxtail millet, were germinated on moist sponge in the auto-controlled growth chamber under 28°C/22°C day/night cycle with a 14 h photoperiod. Seven-day-old, uniform size and healthy seedlings were selected and transferred to a tank containing full nutrient solution. The full nutrient contained 2 mM KNO_3_, 0.25 mM KH_2_PO_4_, 0.75 mM K_2_SO_4_, 0.65 mM MgSO_4_, 2.0 mM CaCl_2_, 0.2 mM Fe-EDTA, 1 μM MnSO_4_, 1 μM ZnSO_4_, 0.1 μM CuSO_4_, 0.005 μM Na_2_MO_7_O_4_ and 1 μM H_3_BO_4_. The solution was renewed every 3 days. On the tenth day, seedlings were transferred to continuous light condition to avoid the effect of photoperiod on gene expression. After 5-day culture, the seedlings were treated with low nitrate solution containing 0.2 mM KNO_3_. To avoid potential potassium deficiency due to the low concentration of KNO_3_ solution, KCl was added to the solution to maintain K level same as those under the higher KNO_3_. Each sample had three biological replications. The roots and shoots were separately harvested at the designated time point.

### Identification of *NPF* genes and analysis of their physical and chemical properties

The NPF protein sequences of Arabidopsis and rice (Léran et al., 2014) were retrieved from TAIR (https://www.arabidopsis.org/) and Phytozome (https://phytozome.jgi.doe.gov/pz/portal.html), respectively. These protein sequences were then blasted against the *xiaomi* genome database (http://sky.sxau.edu.cn/MDSi.htm) using BLASTP algorithm with an E value< 10^-10^ to retrieve putative NPFs. In addition, HMMER program was used to identify NPF genes in *xiaomi*. The Hidden Markov Model (HMM) profile of PTR (PF00854), a conserved domain of NPF proteins, were obtained from pfam 32.0 (http://pfam.xfam.org/). Then the hmmsearch program in HMMERv3.1b2 package (http://hmmer.org/) was used to search for proteins containing PTR domain with “trusted cutoff” as threshold (E-value<10^-10^) based on the local *xiaomi* protein database. The members of NPF proteins identified using BLAST and HMMER were merged as candidate NPF proteins in *xiaomi*. The conserved PTR domain of these candidate NPF proteins was further detected using SMART (http://smart.embl.de/smart/set_mode.cgi?NORMAL=1) and NCBI-CDD (https://www.ncbi.nlm.nih.gov/Structure/cdd/wrpsb.cgi), and proteins without PTR domain were removed. The genomic and CDS sequences of *NPF* genes were downloaded from our MDSi database (http://sky.sxau.edu.cn/MDSi.htm). These *NPF* genes were named using the previous nomenclature (Wang et al., 2018a): *SiNPFX.Y* and *SvNPFX.Y* for foxtail millet and green foxtail millet, respectively. *Si* and *Sv* were the Latin abbreviation for species; X represented for the subfamily, Y for a specific number within the subfamily.

### Homology analysis and phylogenetic tree construction

The MCScanX program was used to analyze homology genes among foxtail millet (*xiaomi*), green foxtail, rice and Arabidopsis, and the result was visualized using CIRCOS software (http://circos.ca/). To investigate the evolutionary relationship of NPF proteins between different species, the full-length NPF amino acid sequences of Arabidopsis, rice, and foxtail millet were aligned by ClustalW in MEGA X with default parameters (https://www.megasoftware.net/). Then, an unrooted neighbor-joining phylogenetic tree was constructed using Treeview.

### Chromosome location, gene duplication and synteny analysis

The chromosome location data of *NPF* genes were obtained from *xiaomi* genomic database (http://sky.sxau.edu.cn/MDSi.htm) and then mapped to the chromosomes with Mapchart (https://www.wur.nl/en/show/Mapchart.htm). The genes failed to be mapped to chromosomes were not shown in the image. Gene duplication event was analyzed using the Multicollinearity Scanning Toolkit (McScanx) server with default parameters. KAKS_Calculator 2.0 was used to calculate the nonsynonymous substitution (ka) and synonymous substitution (ks) for each repeat NPF gene. In order to show the homologous relationship of homologous NPF genes, the homologous analysis map was drawn by Dual Synteny Plotter software (https://github.com/cj-chen/tbtools).

### Gene structure, motif and *cis*-acting elements analysis

The *NPF* exon-intron structures were analyzed using the web-based gene structure display server 2.0 (http://gsds.cbi.pku.edu.cn/). The conserved motifs were analyzed using the online MEME tool (http://meme-suite.org/tools/meme) with the default parameters except that the maximum number of motifs was 10. For promoter analysis, 2.0 kb upstream sequence from the initiation codon (ATG) of each *NPF* was truncated, and then submitted to PlantCARE (http://bioinformatics.psb.ugent.be/webtools/plantcare/html/) to predict *cis*-acting elements.

### Expression analysis of *SiNPF* genes in *xiaomi*

The expression data of 11 different tissues covering the entire lifecycle of *xiaom*i were used to monitor the expression of the above *NPF* genes, including the 3 d imbibed seeds (seed), 2-week-old whole seedling (seedling), root, stem, the top first fully extended leaf of 2-week-old seedling (leaf 1), the top second leaf of 30-day-old plants (leaf 2), flag leaf (leaf 3), the fourth leaf (leaf 4), immature panicle (panicle 1), panicle at pollination stage (panicle 2) and panicle at grain-filling stage (panicle 3). MeV software was used to generate and hierarchical cluster of heatmaps.

### RNA isolation, library construction and sequencing

Total RNA was extracted from root and shoot samples using Plant RNA kit (OMEGA, USA) and RNAprep Pure Plant Kit (Tiangen Biotech Co.,Ltd., Beijing, China), respectively, according to the manufacturer’s protocols. RNA-Seq libraries were constructed using NEBNext Ultra RNA Library Prep Kit for Illumina ((#E7770, New England BioLabs, USA) following the manufacture’s instruction. Briefly, mRNA was purified and fragmented into 200 nt fragments. The fragmented mRNAs were then reverse transcribed to synthesize the first-strand cDNA and the second-strand cDNA. After end repair and adaptor ligation, the products were selected by Agencourt AMPure XP beads (Beckman Coulter, Inc.) and enriched by PCR amplification to create a cDNA library. Finally, the cDNA libraries were sequenced on an Illumina HiSeq X-ten platform.

### RNA-seq reads processing and identification of differential expressed genes

RNA-seq reads were firstly cleaned using Trimmomatic (Bolger et al., 2014) with the following parameters: ILLUMINACLIP : TruSeq3-PE.fa:2:30:10 LEADING:3 TRAILING:3 SLIDINGWINDOW:4:15 MINLEN:30 HEADCROP:10. The clean reads were then mapped to the foxtail millet reference genome (http://sky.sxau.edu.cn/MDSi.htm) using hisat2 (Kim et al., 2015) with default parameters. Gene expression levels were calculated using R with the transcripts per million (TPM) (Li et al., 2010a). Fold changes were calculated using the log_2_ ratio of TPM. Average log values of three biological replications for each sample were then computed and used for further analysis. The cutoff of log_2_-fold changes ≥1 (2-fold absolute value) and padj value ≤ 0.05 were used for selecting significant DEGs.

### Haplotype analysis and verification

Haplotype analysis was performed using the independently developed Perl script CandiHap.pl by our group (https://github.com/xukaili/CandiHap).

### Three-dimensional structure prediction and molecular docking

Three-dimensional (3D) structure of the SiNPFs were predicted using the AlphaFold2 software (Jumper et al., 2021) following the instructions on the website https://github.com/deepmind/alphafold. The obtained 3D structures (pdb files) were then imported into AutoDockTools 1.5.6 for molecular docking analysis (Morris et al., 2009). The 2D and 3D NPF-nitrate interaction model were visualized by Ligplot 2.2.4 (Laskowski and Swindells, 2011) and PyMOL (http://www.pymol.org/), respectively.

### Plasmid construction and complementation of yeast and Arabidopsis mutants

For yeast complementation analysis, the CDS fragments of *SiNRT1.1B1* and *SiNRT1.1B2* were synthesized and cloned into the *Sal* I-*Spe* I sites of the integrative pYNR-EX vector. The constructs were linearized at *Bst*EII in LEU2 and transformed into *Δynt1*, a high-affinity nitrate transporter mutant-deficient strain. Finally, the yeast growth assay was performed as previously described (Martin et al., 2008).

For Arabidopsis complementation analysis, the genomic DNA fragments of *SiNRT1.1B1* and *SiNRT1.1B2* were cloned into pCAMBIA1300 vector, respectively. Due to the large size of the *SiNRT1.1B1* gene, we first amplified two partially overlapped DNA fragments with primer pairs NRT1.1B1F1- NRT1.1B1R1 (fragment 1A) and NRT1.1B1F2- NRT1.2B1R2 (fragment 1B) using KOD-plus DNA polymerase (Toyobo, Osaka, Japan). The fragment 1A was then digested with *Eco*R I-*Xho* I and cloned into *Eco*R I-*Sal* I sites of pCAMBIA1300 to construct the intermediate vector. Finally, the resultant intermediate vector and fragment 1B were digested with *Nru* I and *Nco* I, and ligated to generate the pC1300-SiNRT1.1B1 vector. Similarly, the *SiNRT1.1B2* gene was amplified and inserted into the pCAMBIA1300 vector using appropriate primer pairs and restriction sites to generate pC1300-SiNRT1.1B2 vector. After verification by extensive restriction digestion and DNA sequencing analysis, these constructs were transformed into the *Arabidopsis atnrt1.1* mutant (SALK_097431C) *via Agrobacterium*-mediated floral dip method (Clough and Bent, 1998). All primers used for vector constructions are listed in **Supplementary Table 2**.

## Results

### Genome-wide identification of the *NPF* genes in Setaria

To identify the entire *NPF* genes in Setaria, we performed a BLAST search against x*iaomi* (foxtail millet, http://sky.sxau.edu.cn/MDSi.htm) and A10 (green foxtail) (Bennetzen et al., 2012) genomes using the full-length amino acid sequences of 53 Arabidopsis and 93 rice, repectively. After removing redundant sequences manually, a total of 92 *SiNPF* genes and 88 *SvNPF* genes were retrieved from foxtail millet and green foxtail, respectively (**Supplementary Datasheet 1**).

The length of the SiNPF proteins ranged from 191 amino acid (Si6g12910/SiNPF4.7) to 765 amino acid (Si2g07520/SiNPF2.3), with molecular weight ranging from 21035.05 to 82541.31 Da. Among these *SiNPFs*, 88 genes were unevenly distributed on 9 chromosomes of *xiaomi* except four genes (*Si0g05510*, *Si0g08150*, *Si0g08180* and *Si0g08230*) not assembled on the genome (**Supplementary Figure 1**). There were only two *SiNPF* genes (*Si2g07520* and *Si2g38080*) on chromosome 2, which had the least number of *NPF* genes. There were 26 *SiNPF* genes on chromosome 9, which contained the largest number of *SiNPF* genes. Further analysis revealed that the *SvNPF* genes showed a similar chromosome distribution pattern to the *SiNPF* genes (**Supplementary Figure 2**).

Gene duplication is a main driving force during evolution, which creates the raw genetic materials for natural selection. Of the 92 *SiNPF* genes, 56 genes were involved in duplication events, including 26 tandem gene pairs and 13 segmental duplications gene pairs (**Supplementary Datasheet 2**). The Ka/Ks values of all gene pairs were less than 1, suggesting that these genes were subjected to different levels of purifying selection. Similarly, twenty-three *SvNPF* gene pairs underwent tandem duplication, and 12 gene pairs underwent segmental duplication. The Ka/Ks values were also less than 1 (**Supplementary Datasheet 2**).

**Figure 1:**
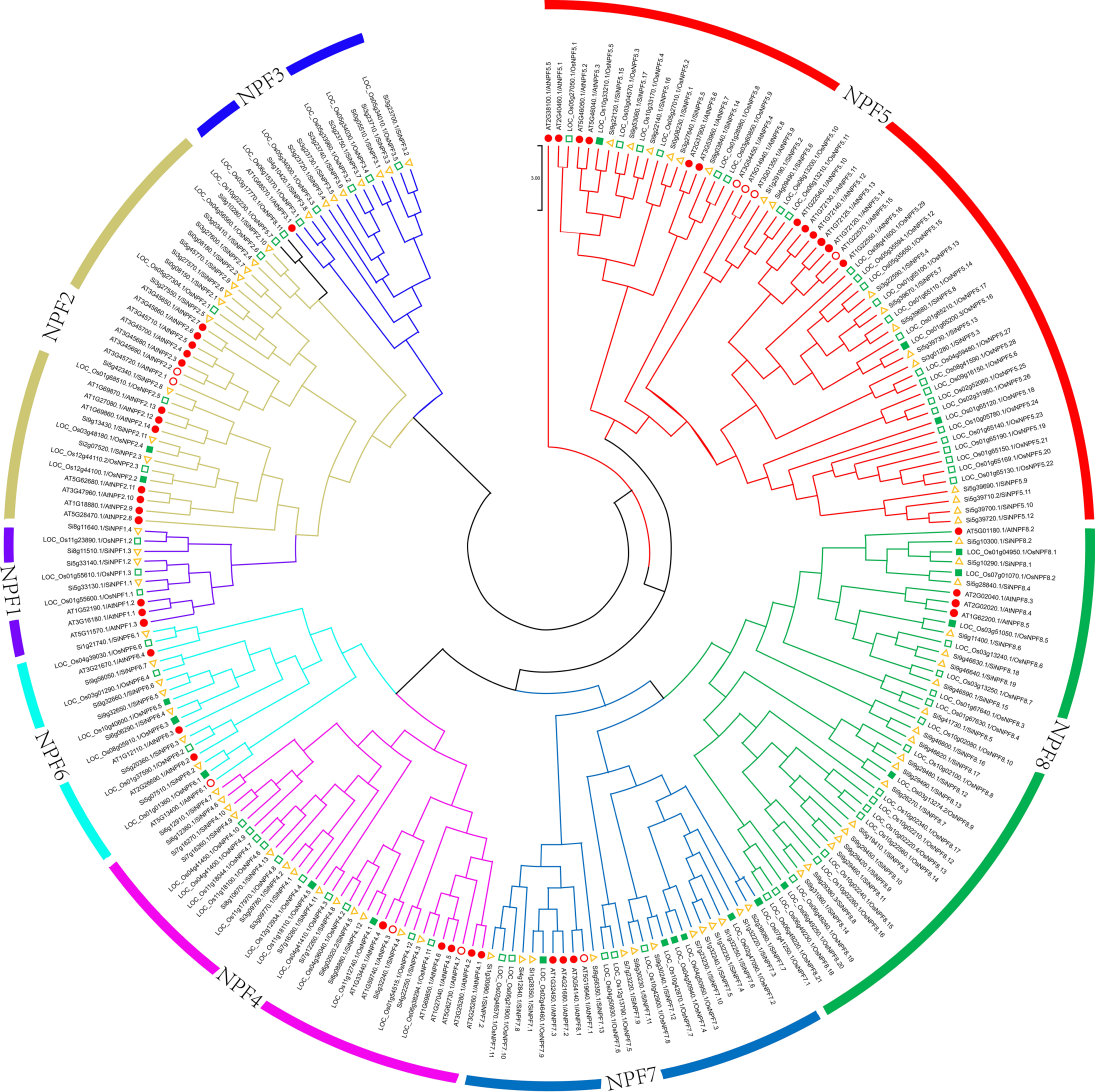
Phylogenetic relationship of NPF proteins among foxtail millet, rice and Arabidopsis. Different subfamilies are color-coded as illustrated in the figure. The red filled circle and the green filled square indicates these *NPFs* have been characterized in *Arabidopsis* (red filled circle) or rice (green filled square). The detailed information of these characterized *NPF* genes were summarized in **Table S1**.

**Figure 2:**
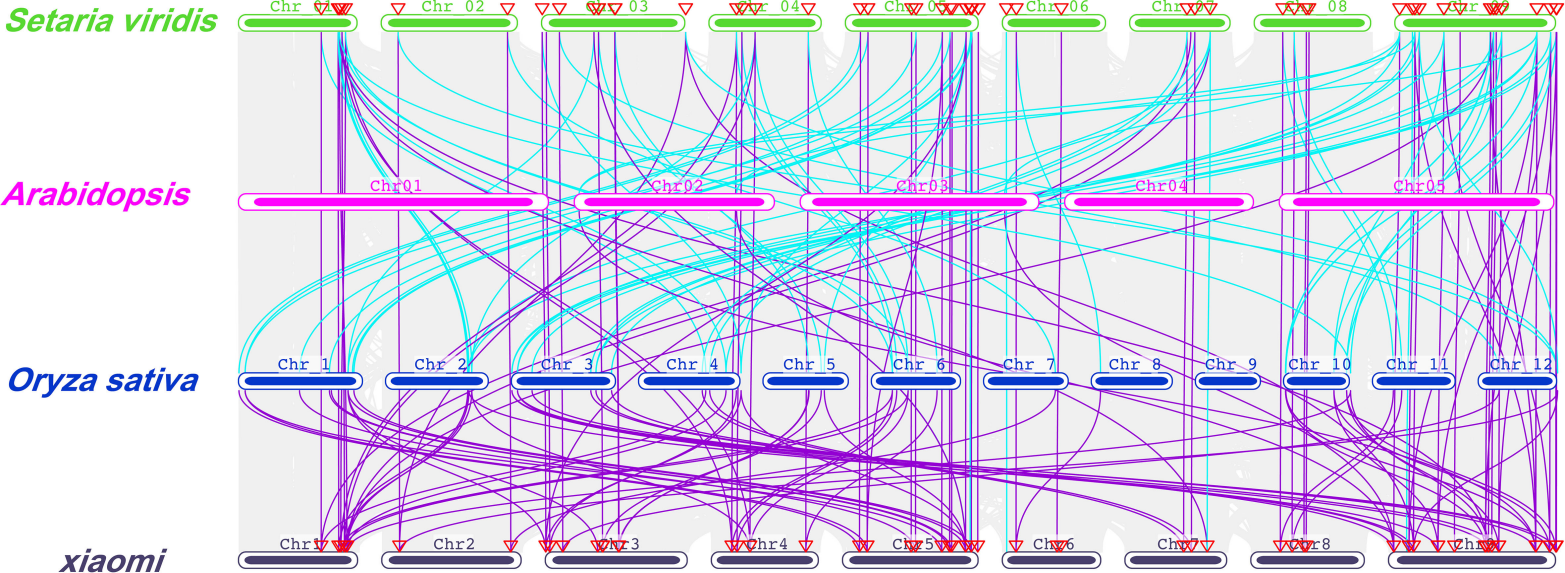
Collinearity of the orthologous *NPF* genes in foxtail millet, green foxtail, rice and Arabidopsis. The genome of each species is shown in one row, and the *NPF* genes of foxtail millet and green foxtail are shown with different colored lines. The collinear relationship of all orthologous genes in different species was shown with the gray lines.

These NPF proteins were well-aligned with NPFs in Arabidopsis and rice, and separated into eight clades (**Figure 1** and **Supplementary Figure 3**). Group eight contains 19 SiNPF proteins, which is the largest group, followed by Group five, which contained 17 SiNPF proteins. There were 38 SiNPFs and 39 SvNPFs in the same branch of rice NPF proteins. The collinear relationship between different species was clearly distinguishable (**Figure 2**). There were three, 59 and 68 orthologous genes of *SiNPFs* were found in Arabidopsis, rice and green foxtail (**Supplementary Datasheet 3**). There were four and 58 orthologous genes of *SvNPF* genes in Arabidopsis and rice. Each orthologous gene pair belonged to the same subfamily. The number of homologous genes of *SiNPF* and *SvNPF* in rice was significantly higher than that in Arabidopsis.

### Gene structure and protein motif analysis of the *NPF* genes in *Setaria*

**Figure 3:**
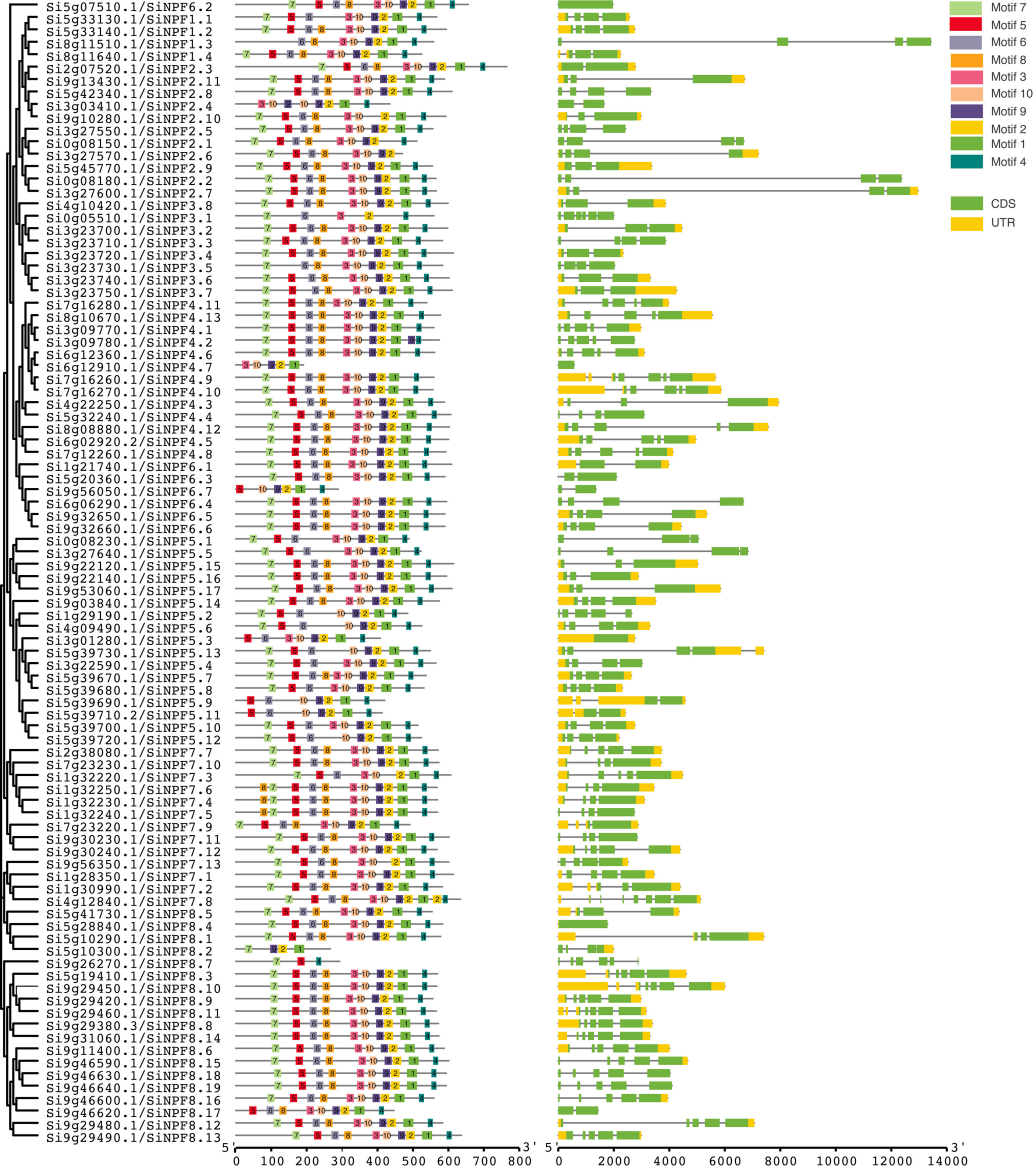
Phylogenetic relationship, gene structure analysis, and motif distributions of *SiNPF* genes. Exon-intron structures are customized in each subfamily (with customized scale bar), the green bar represents exons, the black line represents introns, and the yellow bar represents UTR (untranslated region). The different motifs are color-coded as illustrated in the figure. The scale represents amino-acid length.

To further investigate the structural diversity of *NPF* genes in *Setaria*, the exon-intron organization of these genes were generated based on their coding sequences and corresponding genome sequences (**Figure 3** and **Supplementary Figure 4**). We found that the closely related *NPFs* tended to have similar gene structures with same number of exons and introns. Their differences mainly occurred in the length of UTR and intron regions (**Figure 3**). The number of exons ranged from one to seven. Four genes, *Si3g01280*, *Si5g07510*, *Si5g28840* and *Si6g12910*, had one exon, while *Si4g12840* contained seven exons.

The MEME program was used to search the conserved motifs in these NPF proteins (**Figure 3**). In total, we identified ten conserved motifs. The number of motifs in each NPF varied from three to 11. Approximately two-thirds of the SiNPF (65 out of 92) contained all of the 10 motifs. Although the number of motifs was different, the order of motif 1-10 in NPFs was similar. For instance, motif 7 was located at the C-terminus of all NPF proteins, whereas motif 4 was located at the N-terminus.

### *cis*- regulatory elements and expression atlas of the *SiNPFs*

**Figure 4:**
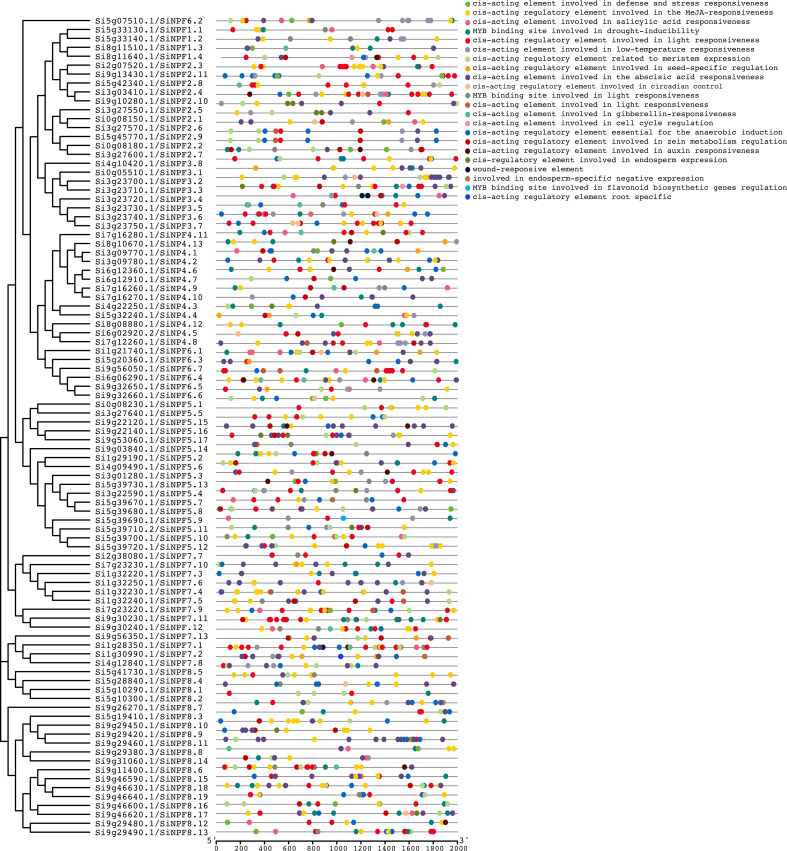
*cis*-acting regulatory elements in the *SiNPF* promoters. Different colored circles represent different types of cis-elements.

The *cis*-regulatory elements (CREs) in the promoter regions provide insights into gene functions. In total, 22 and 21 types of CREs were found in promoter regions of *SiNPF* and *SvNPF* genes, respectively (**Figure 4** and **Supplementary Figure 5**). These elements are involved in growth and development, stress and hormonal responses (ethylene, abscisic acid, auxin, gibberellin, methyl jasmonate, and salicylic acid). A total of 463 light-responsive elements were identified in the promoter regions of all *SiNPF* genes except for *Si0g08230*, which is the most abundant CREs in the *SiNPF* promoters. Among the hormone-responsive elements, the CREs related to the response to methyl jasmonate was the most numerous, followed by abscisic acid. These two hormones have important functions in plant stress response, so the function of *SiNPF* gene may be closely related to these two hormones under low nitrogen stress.

**Figure 5:**
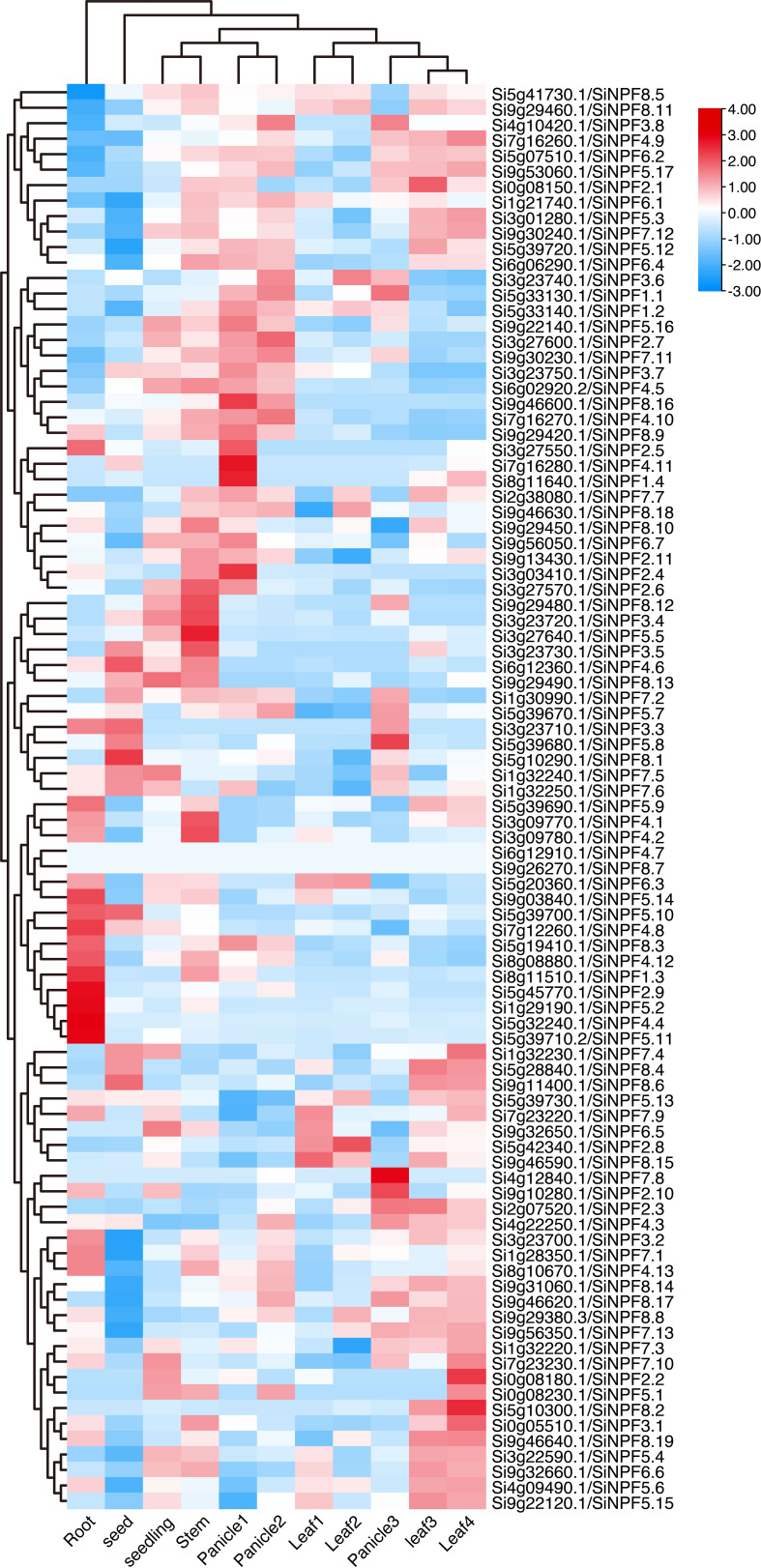
Gene expression atlas of the *SiNPFs* across eleven diverse tissues. 3 d imbibed seeds (seed), 2-week-old whole seedling (seedling), root, stem, the top first fully extended leaf of 2-week-old seedling (leaf 1), the top second leaf of 30-day-old plants (leaf 2), flag leaf (leaf 3), the fourth leaf (leaf 4), immature panicle (panicle 1), panicle at pollination stage (panicle 2) and panicle at grain-filling stage (panicle 3).

To provide a dynamic expression atlas for gene function dissection, we analyzed the gene transcript levels of the *SiNPFs* in eleven diverse tissues representing the major organs over various developmental stages. We found that the *SiNPF* genes expressed in a spatial and temporal manner (**Figure 5** and **Supplementary Datasheet 4**). These *SiNPFs* were divided into two major clusters based on their expression profiles. The first cluster *SiNPFs* were mainly expressed in root, while genes of another cluster were mainly expressed in above ground tissues (including seed, leaf, panicle and stem). Additionally, the young reproductive tissues (panicle 1 and panicle 2), the young leaves (leaf 1 and leaf 2) and the mature leaves (leaf 3 and leaf 4) clustered together strongly, respectively. In particular, *Si1g29190*, *Si5g39710*, *Si5g45770* and *Si9g03840* were highly and preferentially expressed in root, indicating that they might be involved in uptake of nitrate/peptide from soil. Thirty-six *SiNPFs* were highly expressed in stem, which might translocate nitrate from root to shoot. *Si4g12840* was highly expressed in Panicle3, but hardly detected in other tissues. The expression of *Si5g10290* in seed was significantly higher than that in other tissues, while the expression of *Si5g42340* in leaf2 was higher than that in other tissues.

### Time-series low nitrate stress response of the *SiNPF* genes

**Figure 6:**
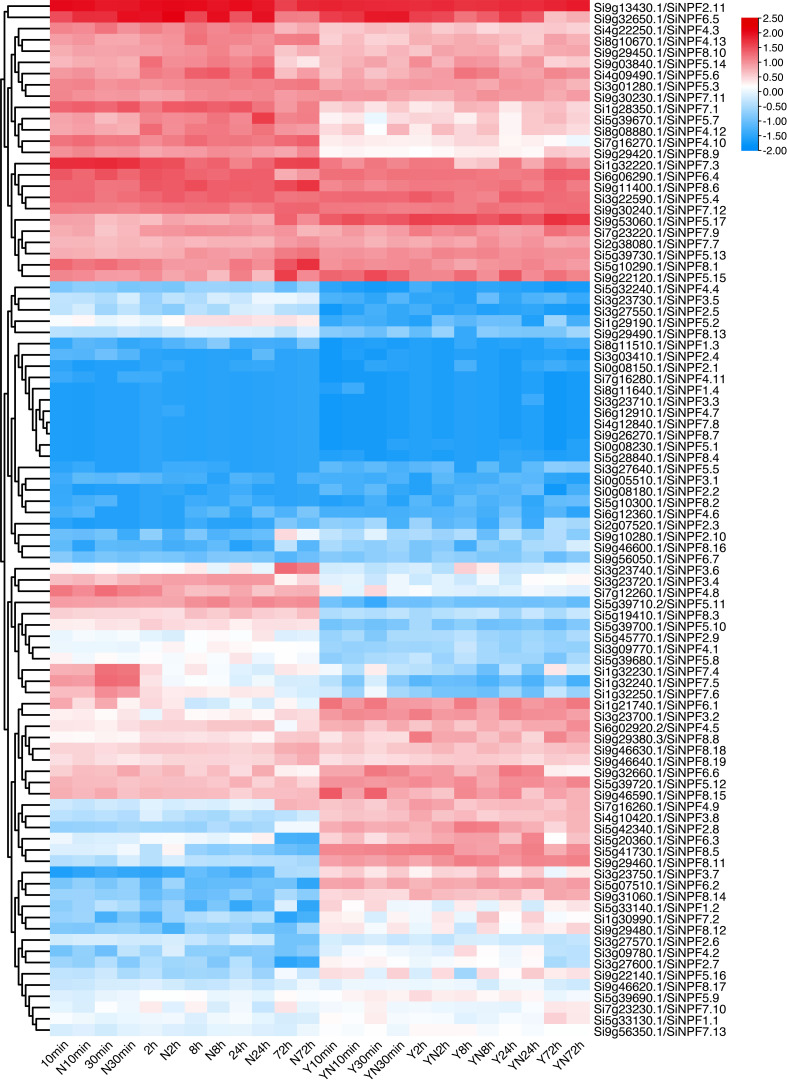
Time-series low nitrate stress response of the *SiNPF* genes.

To gain a better view of the low nitrate response of these *SiNPFs*, we detected the gene expression profile under 10 min, 30 min, 2 h, 8 h, 24 h and 72 h of low nitrate stress. Transcriptome analysis showed that *SiNPF* genes exhibited abundant expression patterns under low nitrogen stress (**Figure 6** and **Supplementary Datasheet 5**). The expression patterns of these *SiNPF* genes were clustered into two broad classes (root and shoots). Eight genes (*Si7g16280*, *Si8g11640*, *Si3g23710*, *Si6g12910*, *Si0g08230*, *Si5g28840*, *Si4g12840* and *Si9g26270*) were hardly expressed under normal N or low N stress. After low nitrogen stress, the number of genes with a foldchange greater than 2 in the shoot (38) was more than that in the root (24). Indeed, more differentially expressed genes (DEGs) were identified at 8h (11), 24h (12) and 72h (12) than at 10min (1), 30min (16) and 2h (10) under LN stress, suggesting the more *SiNPF* genes responded to a longer period of low nitrogen stress. The *Si5g33140* gene was significantly up-regulated in roots after 72 hours of nitrogen stress, predominantly expressed in panicles.

### Natural variations of the *NPF* family genes in foxtail millet

Natural variation is central to understanding gene function, evolution, and which in turn improves breeding in foxtail millet. To provide more information about *SiNPF* genes and facilitate the use of natural accessions, we performed a haplotype analysis based on the re-sequence data of 398 foxtail millet accessions including 162 cultivars, 198 landraces and 38 wild *S. viridis* (Li et al., 2022). A total of 2924 SNPs and 400 InDels were detected in the 92 *SiNPF* genes (**Supplementary Datasheets 6, 7**). These variations exhibited an uneven distribution: the *Si8g11510* contained the highest number of SNPs/InDels, whereas no SNPs/InDels were found in *Si0g05510*, *Si0g08150*, *Si0g08180*, *Si0g08230*, *Si1g32240* and *Si9g26270*. Two SNPs (*Si3g03410* and *Si5g10300*) and 1 InDel (*Si9g29460*) located in splicing site and may cause abnormal splicing of the intron. 245 SNPs/InDels were non-synonymous and were distributed in the coding regions of 53 *SiNPF* genes. Out of the 400 InDels, 22 were found in the coding sequencing and caused frameshift or nonframeshift deletion (*Si1g29190*, *Si2g07520*, *Si3g03410*, *Si7g16280*, *Si8g08880*, *Si9g13430*, *Si9g56350*, *Si5g39700* and *Si8g11640*), insertion (*Si1g29190*, *Si5g10300* and *Si9g13430*) or stop-gain (*Si1g29190*). These *SiNPF* haplotype data provided valuable information for further gene function dissection and molecular design breeding.

### Three-dimensional structure of the SiNPFs and their interaction with nitrate

Protein 3D structure can provide invaluable information to predict its biological function. Thus, we predicted the three-dimensional structure of the NPFs in foxtail millet using AlphaFold2 (Jumper et al., 2021). The SiNPFs members shared a canonical major facilitator superfamily (MFS) fold structure, which was characterized by 12 transmembrane helices (TMs) with a central linker connecting the N-domain (TM1-TM6) and C-domain (TM7-TM12) (**Supplementary Data 1**, **Supplementary Datasheet 8**). Only 15 SiNPFs have few TMs, which are distributed in all except subfamily III (**Supplementary Datasheet 8**). Further analysis revealed that both of the N- and C-terminal structure of the SiNPFs are not conserved, and their exact function in nitrate transport remains to be further elucidated.

**Figure 7:**
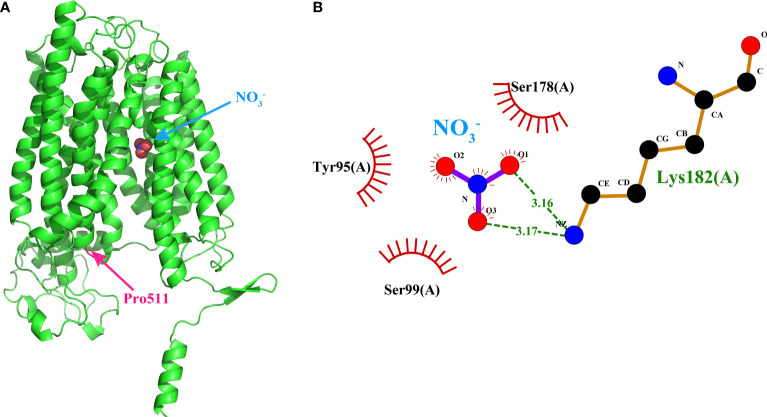
Binding model of Si5g32240 to nitrate by moleculardocking. **(A)** The 3D Si5g32240-nitrate interaction model. The conserved Pro511 was marked in pink. **(B)** The 2D Si5g32240-nitrate interaction model. Dashed lines indicated potential covalent bonds between Lys182 and NO_3_^−^, and the eyelash indicated the non-covalent bonds between Tyr95, Ser99, Ser178 and NO_3_^−^. The3D and 2D interaction models of the other 91 NPFs could be obtained from **Supplementary Data 2** and **3**, respectively.

As nitrate is the major substrate of NPFs, we evaluate theaffinity of the 92 SiNPFs with NO_3_^−^ by molecular docking. Thebinding energy of the SiNPFs to NO_3_^−^ ranged from -3.4 to -2.1 kcal/mol (**Supplementary Datasheet 9**). Among them, Si5g32240 had thelowest binding energy of -3.4 kcal/mol, indicating highly stablebinding. Previously, Ho et al. (2009) reported that the Pro 492 residue of NRT1.1 is important for the nitrate transport activity inArabidopsis. We found that only 10 members do not have theproline residue at the corresponding position, indicating that thisresidue is highly conserved in the SiNPFs (**Supplementary Figure 6**). In the Si5g32240 structure, the conserved proline (Pro511) islocated at the short TMH10-TMH11 loop, but not in thesubstrate binding pocket (**Figure 7A**), indicated that variation ofthe conserved proline might not affect the nitrate binding ability. The 2D Si5g32240- NO_3_^−^ interaction analysis revealed that Tyr95, Ser99, Ser178, and Lys182 might interact with NO3− (**Figure 7B**). These amino acids might play important role in nitrate bind and transport for Si5g32240.

### Tandem duplication of the NRT1.1 gene may contribute to low nitrogen tolerance in foxtail millet

**Figure 8:**
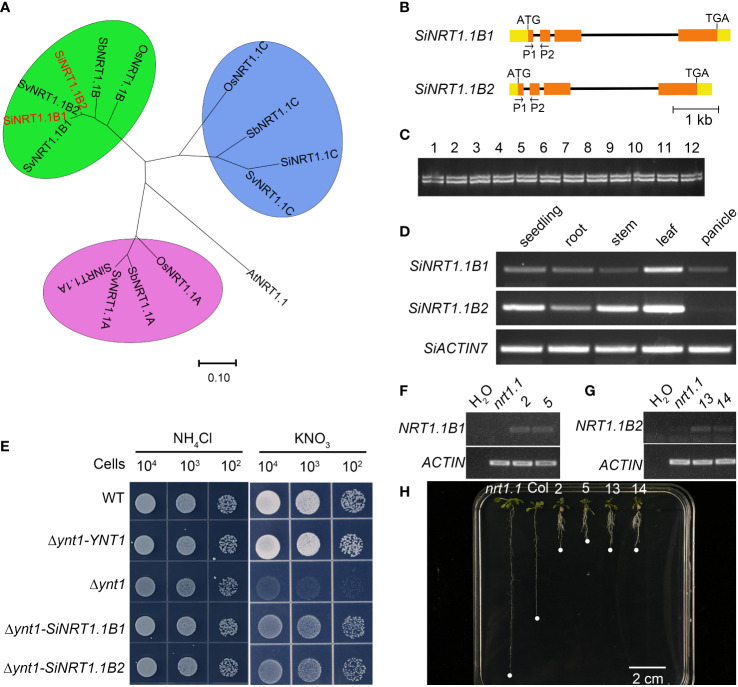
Duplication of the *SiNRT1.1B* gene might confers tolerance to low nitrate in foxtail millet. **(A)** The phylogenetic relationship of the NRT1.1 subfamily in Arabidopsis, rice, sorghum, foxtail millet and green foxtail. AtNRT1.1 (At1G12110), OsNRT1.1A (LOC_Os08g05910), OsNRT1.1B (LOC_Os10g40600), OsNRT1.C (LOC_Os03g01290), SiNRT1.1A (Si6g06290), SiNRT1.1B1 (Si9G32650), SiNRT1.1B2 (Si9G32660), SiNRT1.1C (Si9g56050), SbNRT1.1A (Sobic.007G044300), SbNRT1.1B (Sobic.001G302800), SbNRT1.1C (Sobic.001G541900). **(B)** Gene structure of the *SiNRT1.1B1* and *SiNRT1.1B1* genes. P1 and P2 are primers used for copy number analysis in Panel C **(C)** Copy number analysis of the SiNRT1.1B1 gene in *Setaria*. 1-8 are foxtail millet, and 9-12 are green foxtail. **(D)** The *SiNRT1.1B1* and *SiNRT1.1B2* expression pattern in various organs. **(E)** *SiNRT1.1B1* and *SiNRT1.1B2* rescued the yeast *Δynt1* mutant. *Δynt1*-YNT1, *Δynt1-SiNRT1.1B1* and *Δynt1-SiNRT1.1B2* are *Δynt1* mutant strain transformed with the yeast *YNT1* gene, *SiNRT1.1B1* and *SiNRT1.1B2* gene, respectively. **(F)** RT-PCR analysis of *SiNRT1.B1* expression in *atnrt1.1* mutant (*nrt1.1*), wild type (Col) and two independent *atnrt1.1* transgenic lines carrying a p*SiNRT1.1B1::SiNRT1.1B1* gene (2 and 5). **(G)** RT-PCR analysis of *SiNRT1.B1* expression in *atnrt1.1* mutant (*nrt1.1*), wild type (Col) and two independent *atnrt1.1* transgenic lines carrying a p*SiNRT1.1B2::SiNRT1.1B2* gene (13 and 14). **(H)** *SiNRT1.1B1* and *SiNRT1.1B2* enhanced chlorate sensitivity of the *atnrt1.1* mutant. *nrt1.1* was *atnrt1.1* mutant; Col was the wildtype, 2, 5, 13 and 14 were transgenic lines showed in Panels **(F, G)**.

To dissect the possible molecular mechanisms underlying low nitrogen tolerance of foxtail millet, we performed a synteny analyses of the *NPF* genes between foxtail millet and *Arabidopsis* and rice (**Figure 2** and **Supplementary Datasheet 3**). Interestingly, we found there were two *NRT1.1B* gene copies in a tail-to-tail orientation in both foxtail millet (*Si9g32650/SiNRT1.1B1* and *Si9g32660/SiNRT1.1B2*) and green foxtail (*SvNRT1.1B1/Sevir.9g333900* and *SvNRT1.1B2/Sevir.9g334100*), while there was only one copy in rice and sorghum (**Figure 8A**). Despite of the very high amino acid sequence identity (96.27%) between the two SiNRT1.1Bs, we found the first intron of *SiNRT1.1B1* was 26 bp longer than that of *SiNRT1.1B2* (**Figure 8B**). To determine if there was copy number variation at the *SiNRT1.1Bs* locus in foxtail millet, we performed a PCR analysis using primers spanning the first intron. However, all the germplasm resources detected, including 360 foxtail millets and 38 green foxtails, harbored two copies of *NRT1.1B* gene (**Figure 8C**, and data not shown). This result indicated that the gene duplication events might occur before the divergence of foxtail millet and green foxtail, and after the divergence of the *Setaria* and sorghum.

Duplicated genes usually diverge at the level of gene expression, protein function or both. Thus, it is interesting to know whether the two copies of *NRT1.1B* have similar function or not. As shown in **Figure 5**, *SiNRT1.1B1* and *SiNRT1.1B2* exhibited a similar expression pattern, with highly expressed in vegetative tissues, especially in leaves, but low in reproductive organs. These results were further confirmed by RT-PCR (**Figure 8D**). To further determine whether these two copies could transport nitrate, we transformed them into *Δynt1*, a high affinity nitrate transporter mutant deficient yeast strain (Martin et al., 2008). Unlike wildtype strain, the *Δynt1* mutant could not grow under low nitrate condition. This growth defect could be partially completed by *SiNRT1.1B1* and *SiNRT1.1B2* (**Figure 8E**). To further confirm this result, we transformed the *SiNRT1.1Bs* to the Arabidopsis *nrt1.1* mutant and performed a chlorate-sensitivity assay **Figures 8F–H**. As shown in **Figure 8H**, the transgenic lines of *SiNRT1.1B1* and *SiNRT1.1B2* exhibited higher chlorate sensitivity than that of the *atnrt1.1* mutant. These results clearly demonstrated that both *SiNRT1.1B1* and *SiNRT1.1B2* had nitrate transport activity. Since *NRT1.1B* gene plays an important role in nitrate absorption and transport (Hu et al., 2015), the duplication of the *NRT1.1B* gene may contribute to low nitrogen tolerance of foxtail millet.

**Figure 9:**
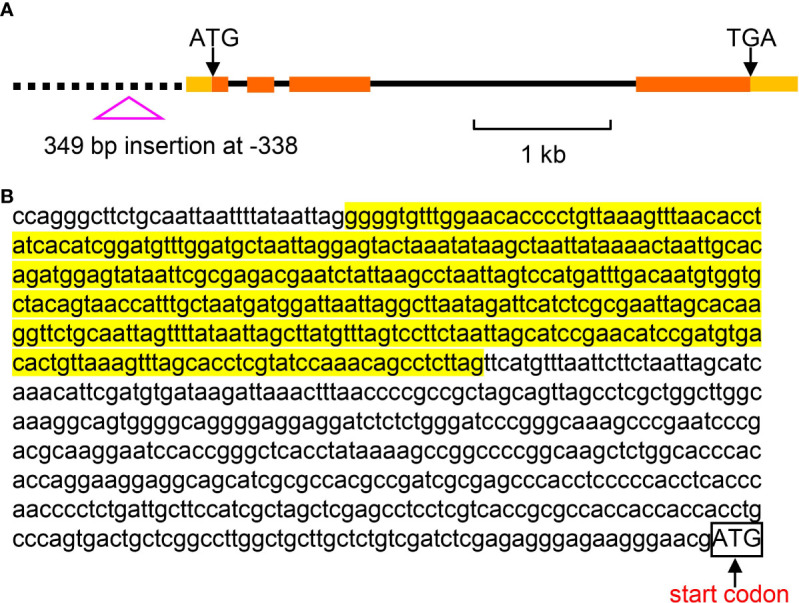
The large InDel in the promoter region of *SiNRT1.1B2*. **(A)** The genomic structure of *SiNRT1.1B2* gene and the large InDel in the promoter. Exons, introns and promoter are denoted by filled boxes, lines and dash line, respectively. **(B)** The nucleotide sequence of the large InDel in the promoter of *SiNRT1.1B2*. The highlighted fragment indicates the 349 bp InDel. The box refers to the start codon.

Due to the relatively short read length, large InDels could not be identified based on the genome re-sequencing data. To provide more genetic variation information, we randomly selected 114 foxtail millet accessions to identify large InDels. Interestingly, we found a 349 bp insertion in the promoter region at nucleotide -398 (the first nucleotide of the putative translation start codon of the genomic sequence is referred to as +1) (**Figure 9**). The effects of this large InDel on gene expression and nitrateuptake need to be further studied.

## Discussion

Genome-wide identification of *NPF* genes have been extensively studied in diverse plant species, including *Arabidopsis* (Léran et al., 2014), rice (Yang et al., 2020a), *Populus tomentosa* (Zhao et al., 2021), *Spirodela polyrhiza* (Lv et al., 2022) and *Triticum aestivum* (Kumar et al., 2022). However, there has been little information about *NPF* family in the barren tolerant species *Setaria*, which greatly limits our understanding of the molecular mechanisms underlying high NUE in this species. In this study, 92 and 88 *NPF* genes were identified in foxtail millet and its wild type ancestor green foxtail, respectively (**Supplementary Datasheet 1**). Further natural variation analysis revealed that there were abundant genetic variations in the *SiNPF* genes (**Supplementary Datasheet 6, 7**). Undoubtedly, our findings provide fundamental information for further research on the biological functions of NPFs and regulation mechanism of barren tolerance in *Setaria*. We also acknowledge that limitations still remain for identification and analysis of the natural variation based on a single reference genome, since a single-reference assembled genome represents only a small fraction information of the entire *Setaria* species. Thus, a species-representative pan-genome is necessary to better capture the full genetic diversity of the NPF gene family in *Setaria*.

The gene number of NPF family varies greatly among species, which is mainly caused by gene duplication including tandem duplication, segmental duplication and transposition events. Our results revealed that tandem duplication and segmental duplication were the driving forces of the *SiNPF* gene expansion (**Supplementary Datasheet 2**). Further analysis revealed that all the *SiNPF* gene pairs underwent strong purifying selection, as evidenced by Ka/Ks ratios lower than 1.0, indicating that function of these genes was highly preserved. Notably, despite the similar number of *NPF* genes in foxtail millet (92) and rice (93), the duplicated events were not exactly the same. We found there were three clades of closely related *AtNRT1.1* genes, *OsNRT1.1A*, *OsNRT1.1B* and *OsNRT1.1C* in rice, while four clades in foxtail millet, namely *SiNRT1.1A*, *SiNRT1.1B1*, *SiNRT1.1B2* and *SiNRT1.1C* (**Figures 1**, **8A**). Among these gene, the *SiNRT1.1B1* and *SiNRT1.1B2* were the tandem duplicated gene pair of *AtNRT1.1*, while there only contains one linear homolog in rice. Interestingly, there is also only one linear homolog of *OsNRT1.1B* in *Sorghum bicolor* L, another Panicoideae species closely to foxtail millet. Therefore, there are at least two genes potentially fill the same functional role of *OsNRT1.1B* in foxtail millet, and this might contribute to the low nitrogen tolerance in *Setaria*. Therefore, it is possible to improve crop NUE by increasing the copy number of *NRT1.1* gene in the future.

The demand for nitrate varies greatly in different tissues or at different growth stages in foxtail millet, therefore nitrate must reach to each of them by different routes (Dechorgnat et al., 2011). Our data showed that approximately one third of the *SiNPF* genes were highly expressed in root, which might play important role in uptake nitrate/peptide or other substrate from soil (**Figure 5**, **Supplementary Datasheet 4**). In additions, *NPFs* that are highly expressed in germinated seed, stem, leaf and panicle were also identified. Since gene express pattern reflects specific tissue functions, these expression data provide valuable clues to understanding their biological function in the future. Notably, we found that many *SiNPF* genes showed similar expression pattern to their orthologous and phylogenetic close Arabidopsis or rice *NPF* genes. For example, the *SiNPF6.3* was predominately expressed in leaves. Similar expression pattern was also reported for the Arabidopsis *AtNPF6.2/AtNRT1:4* (Chiu et al., 2004). Therefore, *SiNPF6.3* is likely to be a key player in regulating leaf nitrate homeostasis and leaf development as *AtNPF6.2*. Differences in expression patterns were also found between *SiNPFs* and their orthologous. Previously, Li et al. (2009) reported that rice *OsNPF4.1/OsSP1* was predominantly expressed in young panicle and with a unique function in panicle development. In contrast to *OsNPF4.1*, the foxtail millet homologous *SiNPF4.12* was highly expressed in roots other than panicles, indicating an additional root function. The functional divergence between orthologues suggests that it is unreliable to predict the NPF function based on their phylogenetic relationships.

The plant NPFs are well known for their essential roles in nitrate uptake, transport and allocation. To date, at least 43 NPFs in Arabidopsis have been characterized, and 23 of them were able to transport nitrate (**Table S1**). Moreover, several NPFs were also been extensively characterized in rice (**Table S1**). However, the substrate(s) and biological functions of NPFs are largely unknown in Seteria, a barren-tolerant species. Here, we provide a solid foundation for further functional dissection of these *NPF* genes. Conventional, gene function could be predicted according to their sequence homology. The collinear analysis showed that there were 3 and 59 linear orthologous genes of *SiNPFs* in Arabidopsis and rice, respectively (**Supplementary Datasheet 3**), which might have similar functions. The Pro492 residue of AtNPF6.3 is highly conserved in Arabidopsis, and is crucial for the nitrate transport activity in Arabidopsis (Ho et al., 2009; Chen et al., 2021). Consistently, there were only 10 SiNPFs that were not proline residue at the corresponding position (**Supplementary Figure 6** **)**. Whether these SiNPFs can transport nitrate remains to be further verified. The *AtNRT1.1* (*AtNPF6.3*) is the most deeply studied *NPF* genes which play important roles in nitrate uptake and signal transduction. The AtNPF6.3- NO_3_^−^ binding energy was -2.4 kcal/ mol. We found that a total 87 SiNPFs had binding energy lower than or equal to -2.4 kcal/mol (**Supplementary Datasheet 9**). These SiNPFs might involve in nitrate uptake and transport in foxtail millet, and should be the focus in future research. In addition to nitrate, some NPFs can also transport phytohormones including auxin, abscisic acid, jasmonates and gibberellins (Corratge-Faillie and Lacombe, 2017). On the other hand, several *NPF* genes involved in phytohormone transport demonstrated hormone- and/or stress- response characteristics (Saito et al., 2015; Tal et al., 2016). In this study, many *cis-*acting element involved in hormone responses were detected in the promoter region of *SiNPF* genes, which suggested their potential hormone-inducing characteristics of these genes (**Figure 3**). Natural variations in *NPF* genes may greatly alter the N uptake and ability. For example, a single-nucleotide polymorphism (SNP) in *OsNRT1.1B* contributed to the NUE divergence between the two main rice subspecies, *indica* and japonica (Hu et al., 2015). Here, we identified 2,924 SNPs and 400 InDels in 92 *SiNPF* genes (**Supplementary Datasheet 6, 7**). Undoubtedly, nonsynonymous or nonsense mutation in *SiNPFs* will be useful for gene function dissection. It is worth mentioning that most synonymous mutations in yeast are extremely harmful, instead of being neutral as generally believed (Shen et al., 2022). If it holds true for genes in foxtail millet, the large amount of synonymous mutation information provided here will be of great value for the future gene function analysis and crop breeding for high NUE. Finally, the accurate 3D structure of the SiNPFs and were also predicted with AlphaFold2. To our knowledge, it is the first NPF family that have 3D structure information.

## Conclusion

In this study, 92 and 88 putative *NPF* genes were identified in foxtail millet and its wild ancestor green foxtail, respectively. These *NPFs* could be divided into eight subfamilies based on sequence similarity and phylogenetic relationship. Among the 92 *SiNPF* genes, about one fourth are highly expressed in root, suggesting that these genes might play roles in nitrate uptake. The time series of transcriptomes provided insight into response of these *SiNPFs* to short- and long- time low nitrate treatments. Interestingly, we found that the *NRT1.1B* gene might contribute to low nitrogen tolerance in *Setaria*. Coupled with the natural variation, 3D information and SiNPF-nitrate interaction models, these results provided the basis for comprehensive understanding of *NPF* genes for low nitrogen tolerance in *Setaria*.

## Data availability statement

The raw RNA-seq data of foxtail millet treated with low nitrate have been deposited in the National Genomics Data Center (https://ngdc.cncb.ac.cn/) under the BioProject accession PRJCA012843. The other sequencing data were obtained from our Multi-omics Database for Setaria italica (MDSi) (http://sky.sxau.edu.cn/MDSi.htm) and JGI Phytozome (https://phytozome-next.jgi.doe.gov/).

## Author contributions

XCW, ZRY conceived and designed the experiments. JJC performed bioinformatics analysis, low nitrate treatment of seedlings. HLT performed 3D structure and natural variation analysis. MS, MMD and YLY participated in data collection. LH and HMS performed molecular docking analysis. XCW and ZRY wrote the manuscript. All authors contributed to the article and approved the submitted version.

## Supplementary material

Supplementary Figure 1 Chromosome location and distribution analysis of the *SiNPF* genes. Tandem duplicated genes are linked by a red curve.

Supplementary Figure 2 Chromosome location and distribution analysis of the *SvNPF* genes. Tandem duplicated genes are linked by a red curve.

Supplementary Figure 3 Phylogenetic relationship of *NPF* proteins among green foxtail, rice and Arabidopsis. Different subfamilies are color-coded as illustrated in the figure. The red filled circle and the green filled square indicates these NPFs have been characterized in Arabidopsis (red filled circle) or rice (green filled square). The detailed information of these characterized NPF genes were summarized in **Table S1**.

Supplementary Figure 4 Phylogenetic relationship, gene-structure analysis, and motif distributions of *SiNPF* genes. Exon-intron structures are customized in each subfamily (with customized scale bar), the green bar represents exons, the black line represents introns, and the yellow bar represents UTR (untranslated region). The different motifs are color-coded as illustrated in the figure. The scale represents amino-acid length.

Supplementary Figure 5
*cis*-acting regulatory elements in the *SvNPF* promoters. Different colored circles represent different types of cis-elements.

Supplementary Figure 6 Amino acid sequence alignment of AtNRT1.1 and SiNPFs. The red star indicates Pro492 in AtNRT1.1.

Supplementary Table 1 Summary of the characterized *NPF* genes in Arabidopsis and rice

Supplementary Table 2 Primers used in this study.
