# Corrigendum: Genome-wide identification and characterization of the NPF genes provide new insight into low nitrogen tolerance in *Setaria*

**DOI:** 10.3389/fpls.2023.1237890

**Published:** 2023-07-28

**Authors:** Jinjin Cheng, Helin Tan, Meng Shan, Mengmeng Duan, Ling Ye, Yulu Yang, Lu He, Huimin Shen, Zhirong Yang, Xingchun Wang

**Affiliations:** ^1^ College of Agriculture, Shanxi Agricultural University, Taigu, China; ^2^ State Key Laboratory of Crop Genetics and Germplasm Enhancement, Nanjing Agricultural University, Nanjing, China; ^3^ College of Life Sciences, Shanxi Agricultural University, Taigu, China; ^4^ Department of Basic Sciences, Shanxi Agricultural University, Taigu, China; ^5^ Shanxi Key Laboratory of Minor Crops Germplasm Innovation and Molecular Breeding, Shanxi Agricultural University, Taigu, China

**Keywords:** *Setaria*, nitrate/peptide transporter, expression profile, natural variation, three-dimensional structure, NRT1.1, low nitrogen tolerance

In our published article, the molecular docking was carried out using blind docking approach, which enclosed the entire protein in the grid box. However, this approach led to inaccuracies in predicting the binding sites as they were not always located within the channel. To address this issue, we utilized the center on ligand approach to define the grid box for docking and manually inspected the resulting conformations. As a result of these changes, **Figure 7** and its caption, as well as **Supplementary Data 2** ; **3** , **Supplementary Datasheet 9** and the related text have been corrected.

**Figure 7:**
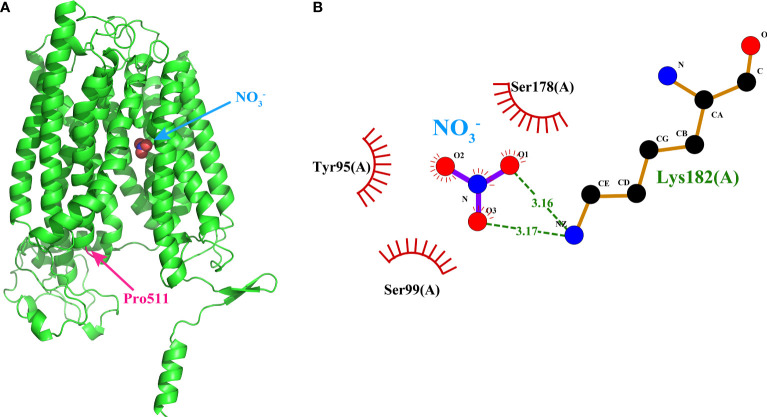
Binding model of Si5g32240 to nitrate by molecular docking. **(A)** The 3D Si5g32240-nitrate interaction model. The conserved Pro511 was marked in pink. **(B)** The 2D Si5g32240-nitrate interaction model. Dashed lines indicated potential covalent bonds between Lys182 and NO_3_^−^, and the eyelash indicated the non-covalent bonds between Tyr95, Ser99, Ser178 and NO_3_^−^. The 3D and 2D interaction models of the other 91 NPFs could be obtained from Supplementary Data 2 and Supplementary Data 3, respectively.

In the published article, there was an error in the legend for **Figure 7**. In the new binding model predicted using the center on ligand approach, Si5g32240, instead of Si5g10290, Si8g08880, and Si8g11510, had the lowest binding energy. Therefore, the 3D and 2D interaction models of Si5g32240-nitrate but not Si8G11510-nitrate should be presented in **Figure 7**. The legend of **Figure 7** should be corrected to reflect this change, with “Si5g32240” instead of “Si8G11510”. We also want to clarify that the conserved proline in Si5g32240 is Pro511, and the amino acids interacting with NO_3_^-^ were Tyr95, Ser99, Ser178, and Lys182.

In the published article, there was an error in **Figure 7**. The 3D and 2D interaction models of Si5g32240-nitrate but not Si8G11510-nitrate in the published article, should be presented in **Figure 7**. The corrected **Figure 7** Binding model of Si5g32240 to nitrate by molecular docking and its caption **Figure 7** Binding model of Si5g32240 to nitrate by molecular docking. (A) The 3D Si5g32240-nitrate interaction model. The conserved Pro511 was marked in pink. (B) The 2D Si5g32240-nitrate interaction model. Dashed lines indicated potential covalent bonds between Lys182 and NO_3_^−^, and the eyelash indicated the non-covalent bonds between Tyr95, Ser99, Ser178 and NO_3_^−^. The 3D and 2D interaction models of the other 91 NPFs could be obtained from **Supplementary Data 2** and **3** , respectively. appear below.

“**Figure 7** Binding model of Si5g32240 to nitrate by molecular docking. (A) The 3D Si5g32240-nitrate interaction model. The conserved Pro511 was marked in pink. (B) The 2D Si5g32240-nitrate interaction model. Dashed lines indicated potential covalent bonds between Lys182 and NO_3_^−^, and the eyelash indicated the non-covalent bonds between Tyr95, Ser99, Ser178 and NO_3_^−^. The 3D and 2D interaction models of the other 91 NPFs could be obtained from **Supplementary Data 2** and **3** , respectively.”

In the published article, there was an error in **Supplementary Data 2** . Previously, the 3D interaction models were previously predicted using the blind docking approach. To obtained more accuracy data, we re-predicted 3D interaction models using the center on ligand approach. As a results, the 3D interaction models of the SiNPF-nitrate changed.

In the published article, there was an error in **Supplementary Data 3** . The 2D interaction models were derived from the 3D interaction models presented in **Supplementary Data 2** . Due to modifications made to the 3D models, the 2D interaction models of SiNPF-nitrate were also changed accordingly.

In the published article, there was an error in **Supplementary Datasheet 9** . The affinity energy presented in **Supplementary Datasheet 9** was calculated based on the 3D interaction models presented in **Supplementary Data 2** . As modifications were made to the 3D models, the affinity energy of SiNPF-nitrate was also changed accordingly.

In the published article, there were several errors in the text. The re-calculated binding energy range of SiNPFs-NO_3_^−^ was found to be -3.4 to -2.1 kcal/mol instead of -3.8 to -2.7 kcal/mol as previously reported. We also found that Si5g32240, instead of Si5g10290, Si8g08880, and Si8g11510, had the lowest binding energy. Furthermore, the conserved proline and the amino acids binding to NO_3_^−^ should be Pro511 and Tyr95, Ser99, Ser178, and Lys182 in Si5g32240, respectively.

A correction has been made to **Abstract**, *Results*, Paragraph 3. This sentence previously stated:

“We found that the SiNPFs NO_3_^−^ binding energy ranged from -3.8 to -2.7 kcal/mol.”

The corrected sentence appears below:

“We found that the SiNPFs NO_3_^−^ binding energy ranged from -3.4 to -2.1 kcal/mol.”

Corrections have also been made to **Results**, *Three-dimensional structure of the SiNPFs and their interaction with nitrate*, Paragraph 2. These sentences previously stated:

“As nitrate is the major substrate of NPFs, we evaluate the affinity of the 92 SiNPFs with NO_3_^−^ by molecular docking. The binding energy of the SiNPFs to NO_3_^−^ ranged from -3.8 to -2.7 kcal/mol (**Supplementary Datasheet 9**). Among them, Si5g10290, Si8g08880 and Si8g11510 had the lowest binding energy of -3.8, indicating highly stable binding. Previously, Ho et al. (2009) reported that the Pro 492 residue of NRT1.1 is important for the nitrate transport activity in Arabidopsis. We found that only 10 members do not have the proline residue at the corresponding position, indicating that this residue is highly conserved in the SiNPFs (**Supplementary Figure 6**). In the Si8g11510 structure, the conserved proline (Pro457) is located at the short TMH10-TMH11 loop, but not in the substrate binding pocket (**Figure 7A**), indicated that variation of the conserved proline might not affect the nitrate binding ability. The 2D Si8g11510- NO_3_^−^ interaction analysis revealed that Gln433 and Tyr512 directly binding to NO_3_^−^ (**Figure 7B**). These two amino acids might play important role in nitrate bind and transport for Si8g11510.”

The corrected sentences appear below:

“As nitrate is the major substrate of NPFs, we evaluate the affinity of the 92 SiNPFs with NO_3_^−^ by molecular docking. The binding energy of the SiNPFs to NO_3_^−^ ranged from -3.4 to -2.1 kcal/mol (**Supplementary Datasheet 9** ). Among them, Si5g32240 had the lowest binding energy of -3.4 kcal/mol, indicating highly stable binding. Previously, Ho et al. (2009) reported that the Pro 492 residue of NRT1.1 is important for the nitrate transport activity in Arabidopsis. We found that only 10 members do not have the proline residue at the corresponding position, indicating that this residue is highly conserved in the SiNPFs (**Supplementary Figure 6** ). In the Si5g32240 structure, the conserved proline (Pro511) is located at the short TMH10-TMH11 loop, but not in the substrate binding pocket (**Figure 7A**), indicated that variation of the conserved proline might not affect the nitrate binding ability. The 2D Si5g32240- NO_3_^−^ interaction analysis revealed that Tyr95, Ser99, Ser178, and Lys182 might interact with NO_3_^−^ (**Figure 7B**). These amino acids might play important role in nitrate bind and transport for Si5g32240.”

In the published article, there was an error. The N- terminal contains a ExxER motif, which is an important structural feature in NPFs. Thus, the N-terminal might play important role, rather than not essential for the transport ability.

A correction has been made to **Results**, *Three-dimensional structure of the SiNPFs and their interaction with nitrate*, Paragraph 1. This sentence previously stated:

“Further analysis revealed that both of the N- and C-terminal structure of the SiNPFs is not conserved, indicating that both terminals were not essential for the transport ability.”

The corrected sentence appears below:

“Further analysis revealed that both of the N- and C-terminal structure of the SiNPFs are not conserved, and their exact function in nitrate transport remains to be further elucidated.”

In the published article, there was an error. These two large InDels should be this large InDel.

A correction has been made to **Results**, *Tandem duplication of the NRT1.1 gene may contribute to low nitrogen tolerance in foxtail millet*, Paragraph 3. This sentence previously stated:

“The effects of these two large InDels on gene expression and nitrate uptake need to be further studied.”

The corrected sentence appears below:

“The effects of this large InDel on gene expression and nitrate uptake need to be further studied.”

In the published article, there were two errors. 1) The AtNPF6.3- NO3- binding energy was incorrectly stated as -3.2 kcal/mol, whereas the correct value is -2.4 kcal/mol instead of. 2) The number of SiNPFs with a binding energy lower than or equal to -2.4 kcal/mol was 87, not 58.

A correction has been made to Discussion, Paragraph 4. These sentences previously stated:

“The AtNPF6.3- NO_3_^−^ binding energy was -3.2 kcal/ mol. We found that a total 58 SiNPFs had binding energy lower than or equal to -3.2 kcal/mol (Supplementary Datasheet 9).”

The corrected sentence appears below:

“The AtNPF6.3- NO_3_^−^ binding energy was -2.4 kcal/ mol. We found that a total 87 SiNPFs had binding energy lower than or equal to -2.4 kcal/mol (Supplementary Datasheet 9)”

The authors apologize for these errors and state that this does not change the scientific conclusions of the article in any way. The original article has been updated.

