# Supplementary material for: Genome-wide identification and characterization of the NPF genes provide new insight into low nitrogen tolerance in Setaria
Source: Front Plant Sci. 2022 Dec 14;13:1043832. doi: 10.3389/fpls.2022.1043832 (PMC9795848; doi:10.3389/fpls.2022.1043832)
Supplement: Supplementary Figure 1 — Chromosome location and distribution analysis of the SiNPF genes. Tandem duplicated genes are linked by a red curve. [file DataSheet_1.zip › Supplementary Figure 5.pdf]

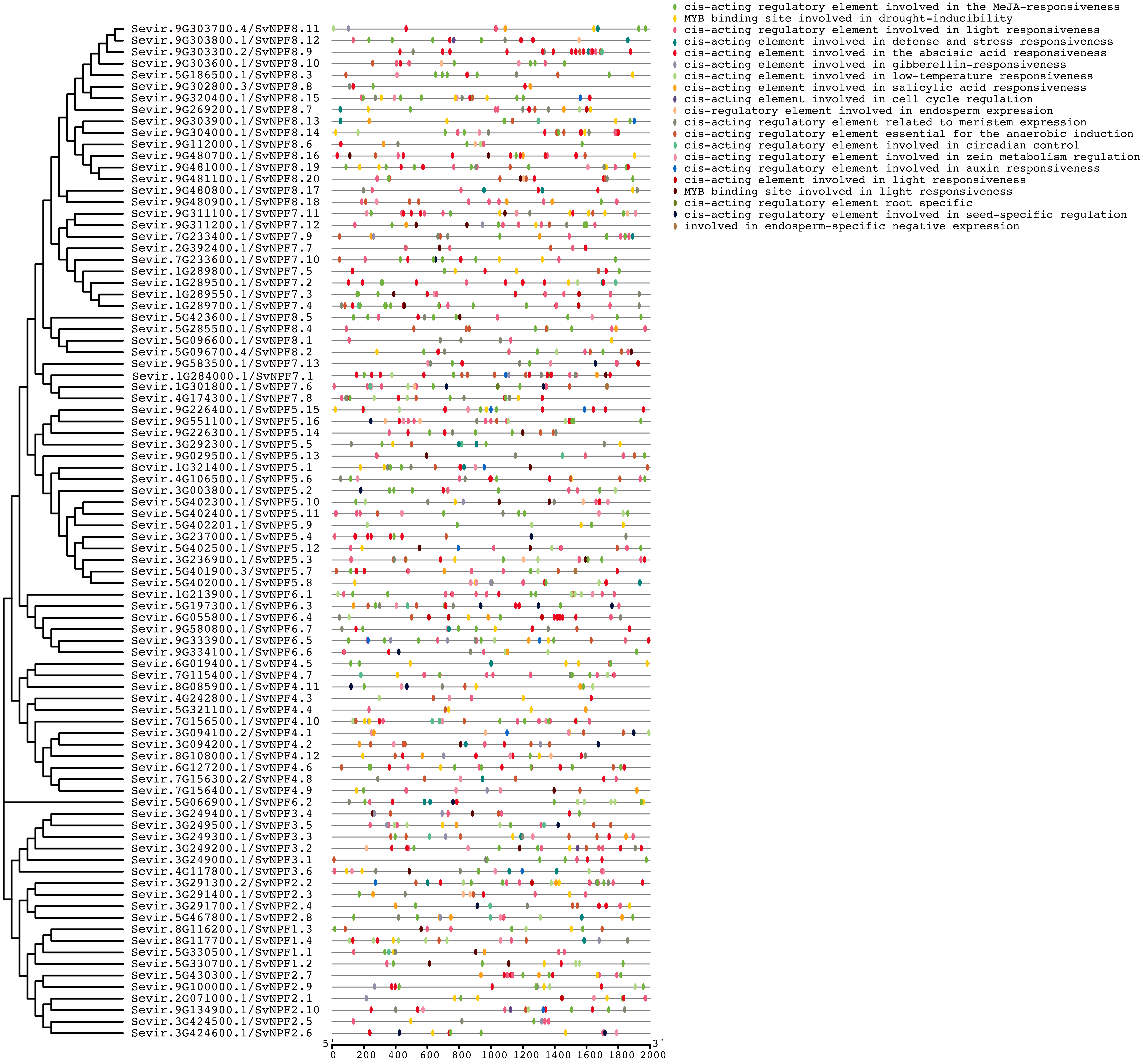
Sevir.9G303700.4/SvNPF8.11
Sevir.9G303800.1/SvNPF8.12
Sevir.9G303300.2/SvNPF8.9
Sevir.9G303600.1/SvNPF8.10
Sevir.5G186500.1/SvNPF8.3
Sevir.9G302800.3/SvNPF8.8
Sevir.9G320400.1/SvNPF8.15
Sevir.9G269200.1/SvNPF8.7
Sevir.9G303900.1/SvNPF8.13
Sevir.9G304000.1/SvNPF8.14
Sevir.9G112000.1/SvNPF8.6
Sevir.9G480700.1/SvNPF8.16
Sevir.9G481000.1/SvNPF8.19
Sevir.9G481100.1/SvNPF8.20
Sevir.9G480800.1/SvNPF8.17
Sevir.9G480900.1/SvNPF8.18
Sevir.9G311100.1/SvNPF7.11
Sevir.9G311200.1/SvNPF7.12
Sevir.7G233400.1/SvNPF7.9
Sevir.2G392400.1/SvNPF7.7
Sevir.7G233600.1/SvNPF7.10
Sevir.1G289800.1/SvNPF7.5
Sevir.1G289500.1/SvNPF7.2
Sevir.1G289550.1/SvNPF7.3
Sevir.1G289700.1/SvNPF7.4
Sevir.5G423600.1/SvNPF8.5
Sevir.5G285500.1/SvNPF8.4
Sevir.5G096600.1/SvNPF8.1
Sevir.5G096700.4/SvNPF8.2
Sevir.9G583500.1/SvNPF7.13
Sevir.1G284000.1/SvNPF7.1
Sevir.1G301800.1/SvNPF7.6
Sevir.4G174300.1/SvNPF7.8
Sevir.9G226400.1/SvNPF5.15
Sevir.9G551100.1/SvNPF5.16
Sevir.9G226300.1/SvNPF5.14
Sevir.3G292300.1/SvNPF5.5
Sevir.9G029500.1/SvNPF5.13
Sevir.1G321400.1/SvNPF5.1
Sevir.4G106500.1/SvNPF5.6
Sevir.3G003800.1/SvNPF5.2
Sevir.5G402300.1/SvNPF5.10
Sevir.5G402400.1/SvNPF5.11
Sevir.5G402201.1/SvNPF5.9
Sevir.3G237000.1/SvNPF5.4
Sevir.5G402500.1/SvNPF5.12
Sevir.3G236900.1/SvNPF5.3
Sevir.5G401900.3/SvNPF5.7
Sevir.5G402000.1/SvNPF5.8
Sevir.1G213900.1/SvNPF6.1
Sevir.5G197300.1/SvNPF6.3
Sevir.6G055800.1/SvNPF6.4
Sevir.9G580800.1/SvNPF6.7
Sevir.9G333900.1/SvNPF6.5
Sevir.9G334100.1/SvNPF6.6
Sevir.6G019400.1/SvNPF4.5
Sevir.7G115400.1/SvNPF4.7
Sevir.8G085900.1/SvNPF4.11
Sevir.4G242800.1/SvNPF4.3
Sevir.5G321100.1/SvNPF4.4
Sevir.7G156500.1/SvNPF4.10
Sevir.3G094100.2/SvNPF4.1
Sevir.3G094200.1/SvNPF4.2
Sevir.8G108000.1/SvNPF4.12
Sevir.6G127200.1/SvNPF4.6
Sevir.7G156300.2/SvNPF4.8
Sevir.7G156400.1/SvNPF4.9
Sevir.5G066900.1/SvNPF6.2
Sevir.3G249400.1/SvNPF3.4
Sevir.3G249500.1/SvNPF3.5
Sevir.3G249300.1/SvNPF3.3
Sevir.3G249200.1/SvNPF3.2
Sevir.3G249000.1/SvNPF3.1
Sevir.4G117800.1/SvNPF3.6
Sevir.3G291300.2/SvNPF2.2
Sevir.3G291400.1/SvNPF2.3
Sevir.3G291700.1/SvNPF2.4
Sevir.5G467800.1/SvNPF2.8
Sevir.8G116200.1/SvNPF1.3
Sevir.8G117700.1/SvNPF1.4
Sevir.5G330500.1/SvNPF1.1
Sevir.5G330700.1/SvNPF1.2
Sevir.5G430300.1/SvNPF2.7
Sevir.9G100000.1/SvNPF2.9
Sevir.2G071000.1/SvNPF2.1
Sevir.9G134900.1/SvNPF2.10
Sevir.3G424500.1/SvNPF2.5
Sevir.3G424600.1/SvNPF2.6
5'
3'
0
200
400
600
800
1000
1200
1400
1600
1800
2000
cis-acting regulatory element involved in the MeJA-responsiveness
MYB binding site involved in drought-inducibility
cis-acting regulatory element involved in light responsiveness
cis-acting element involved in defense and stress responsiveness
cis-acting element involved in the abscisic acid responsiveness
cis-acting element involved in gibberellin-responsiveness
cis-acting element involved in low-temperature responsiveness
cis-acting element involved in salicylic acid responsiveness
cis-acting element involved in cell cycle regulation
cis-regulatory element involved in endosperm expression
cis-acting regulatory element related to meristem expression
cis-acting regulatory element essential for the anaerobic induction
cis-acting regulatory element involved in circadian control
cis-acting regulatory element involved in zein metabolism regulation
cis-acting regulatory element involved in auxin responsiveness
cis-acting element involved in light responsiveness
MYB binding site involved in light responsiveness
cis-acting regulatory element root specific
cis-acting regulatory element involved in seed-specific regulation
involved in endosperm-specific negative expression
