# Supplementary material for: Genome-wide identification and characterization of the NPF genes provide new insight into low nitrogen tolerance in Setaria
Source: Front Plant Sci. 2022 Dec 14;13:1043832. doi: 10.3389/fpls.2022.1043832 (PMC9795848; doi:10.3389/fpls.2022.1043832)
Supplement: Supplementary Figure 1 — Chromosome location and distribution analysis of the SiNPF genes. Tandem duplicated genes are linked by a red curve. [file DataSheet_1.zip › Supplementary Figure 6.pdf]

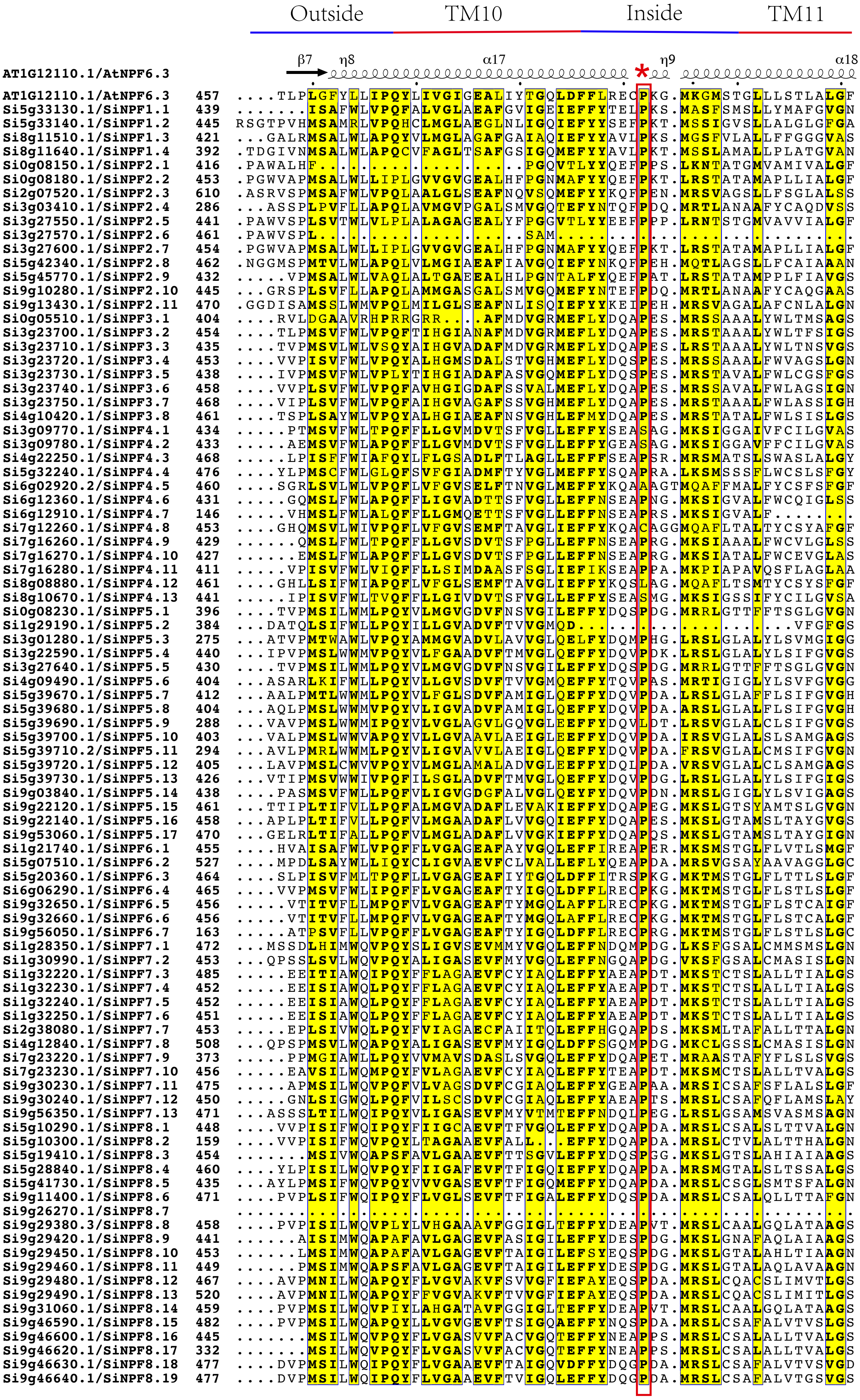
Outside
TM10
Inside
TM11
β7
η8
α17
η9
α18
AT1G12110.1/AtNPF6.3
AT1G12110.1/AtNPF6.3 457
Si5g33130.1/SiNPF1.1 439
Si5g33140.1/SiNPF1.2 445
Si8g11510.1/SiNPF1.3 421
Si8g11640.1/SiNPF1.4 392
Si0g08150.1/SiNPF2.1 416
Si0g08180.1/SiNPF2.2 453
Si2g07520.1/SiNPF2.3 610
Si3g03410.1/SiNPF2.4 286
Si3g27550.1/SiNPF2.5 441
Si3g27570.1/SiNPF2.6 461
Si3g27600.1/SiNPF2.7 454
Si5g42340.1/SiNPF2.8 462
Si5g45770.1/SiNPF2.9 432
Si9g10280.1/SiNPF2.10 445
Si9g13430.1/SiNPF2.11 470
Si0g05510.1/SiNPF3.1 404
Si3g23700.1/SiNPF3.2 454
Si3g23710.1/SiNPF3.3 435
Si3g23720.1/SiNPF3.4 453
Si3g23730.1/SiNPF3.5 438
Si3g23740.1/SiNPF3.6 458
Si3g23750.1/SiNPF3.7 468
Si4g10420.1/SiNPF3.8 461
Si3g09770.1/SiNPF4.1 434
Si3g09780.1/SiNPF4.2 433
Si4g22250.1/SiNPF4.3 468
Si5g32240.1/SiNPF4.4 476
Si6g02920.2/SiNPF4.5 460
Si6g12360.1/SiNPF4.6 431
Si6g12910.1/SiNPF4.7 146
Si7g12260.1/SiNPF4.8 453
Si7g16260.1/SiNPF4.9 429
Si7g16270.1/SiNPF4.10 427
Si7g16280.1/SiNPF4.11 411
Si8g08880.1/SiNPF4.12 461
Si8g10670.1/SiNPF4.13 441
Si0g08230.1/SiNPF5.1 396
Si1g29190.1/SiNPF5.2 384
Si3g01280.1/SiNPF5.3 275
Si3g22590.1/SiNPF5.4 440
Si3g27640.1/SiNPF5.5 430
Si4g09490.1/SiNPF5.6 404
Si5g39670.1/SiNPF5.7 412
Si5g39680.1/SiNPF5.8 404
Si5g39690.1/SiNPF5.9 288
Si5g39700.1/SiNPF5.10 403
Si5g39710.2/SiNPF5.11 294
Si5g39720.1/SiNPF5.12 405
Si5g39730.1/SiNPF5.13 426
Si9g03840.1/SiNPF5.14 438
Si9g22120.1/SiNPF5.15 461
Si9g22140.1/SiNPF5.16 458
Si9g53060.1/SiNPF5.17 470
Si1g21740.1/SiNPF6.1 455
Si5g07510.1/SiNPF6.2 527
Si5g20360.1/SiNPF6.3 464
Si6g06290.1/SiNPF6.4 465
Si9g32650.1/SiNPF6.5 456
Si9g32660.1/SiNPF6.6 456
Si9g56050.1/SiNPF6.7 163
Si1g28350.1/SiNPF7.1 472
Si1g30990.1/SiNPF7.2 453
Si1g32220.1/SiNPF7.3 485
Si1g32230.1/SiNPF7.4 452
Si1g32240.1/SiNPF7.5 452
Si1g32250.1/SiNPF7.6 451
Si2g38080.1/SiNPF7.7 453
Si4g12840.1/SiNPF7.8 508
Si7g23220.1/SiNPF7.9 373
Si7g23230.1/SiNPF7.10 456
Si9g30230.1/SiNPF7.11 475
Si9g30240.1/SiNPF7.12 450
Si9g56350.1/SiNPF7.13 471
Si5g10290.1/SiNPF8.1 448
Si5g10300.1/SiNPF8.2 159
Si5g19410.1/SiNPF8.3 454
Si5g28840.1/SiNPF8.4 460
Si5g41730.1/SiNPF8.5 435
Si9g11400.1/SiNPF8.6 471
Si9g26270.1/SiNPF8.7
Si9g29380.3/SiNPF8.8 458
Si9g29420.1/SiNPF8.9 441
Si9g29450.1/SiNPF8.10 453
Si9g29460.1/SiNPF8.11 449
Si9g29480.1/SiNPF8.12 467
Si9g29490.1/SiNPF8.13 520
Si9g31060.1/SiNPF8.14 459
Si9g46590.1/SiNPF8.15 482
Si9g46600.1/SiNPF8.16 445
Si9g46620.1/SiNPF8.17 332
Si9g46630.1/SiNPF8.18 477
Si9g46640.1/SiNPF8.19 477
