# Supplementary material for: Genome-wide identification and characterization of the NPF genes provide new insight into low nitrogen tolerance in Setaria
Source: Front Plant Sci. 2022 Dec 14;13:1043832. doi: 10.3389/fpls.2022.1043832 (PMC9795848; doi:10.3389/fpls.2022.1043832)
Supplement: Supplementary Figure 1 — Chromosome location and distribution analysis of the SiNPF genes. Tandem duplicated genes are linked by a red curve. [file DataSheet_1.zip › Supplementary Table 1.docx]

**Supplementary Table 1| Summary of the characterized *NPF* genes in Arabidopsis and rice**

| **NPF name** | **Gene Number** | **Old name** | **Gene expression pattern** | **Subcellular location** | **Substrates** | **Function annotation** | **Reference** |
| --- | --- | --- | --- | --- | --- | --- | --- |
| ***Arabidopsis thaliana*** | | | | | | | |
| AtNPF1.1 | *AT3G16180* | NRT1.12 | The phloem of the major veins of leaves | Plasma membrane | NO_3_^-^, ABA, GA, JA-Ile | Nitrate redistribution to young leaves | (Hsu and Tsay, 2013; Chiba et al., 2015) |
| AtNPF1.2 | *AT1G52190* | NRT1.11 | The phloem of the major veins of leaves | Plasma membrane | NO_3_-, GA, JA-Ile | Nitrate redistribution to young leaves | (Hsu and Tsay, 2013; Chiba et al., 2015) |
| AtNPF1.3 | *AT5G11570* | *NA* | ND | ND | NO_3_^-^ | Nitrate transport | (Chen and Ho, 2022) |
| AtNPF2.3 | *At3G45680* | *NA* | Root pericycle cells | Plasma membrane | NO_3_-, GA | Nitrate translocation to shoots under salt stress | (Chiba et al., 2015; Taochy et al., 2015) |
| AtNPF2.4 | *At3G45700* | NA | Root stele | Plasma membrane | Cl-, GA, JA-Ile | Loading and regulation of Cl^–^ into the xylem of roots during salinity stress | (Chiba et al., 2015; Li et al., 2016a) |
| AtNPF2.5 | *At3G45710* | NA | Root cortical cells | Plasma membrane | Cl-, ABA, GA | Chloride efflux from the root exclusion of Cl^−^ from the shoot | (Chiba et al., 2015; Li et al., 2016b) |
| AtNPF2.6 | *At3G45660* | NA | ND | ND | GA, JA-Ile | ND | (Li et al., 2016a) |
| AtNPF2.7 | *At3G45650* | NAXT1 | Cortex of mature roots | Plasma membrane | NO_3_-, GA, JA-Ile | Nitrate efflux in roots under acidic conditions | (Segonzac et al., 2007; Chiba et al., 2015) |
| AtNPF2.8 | *At5G28470* | NA | ND | Plasma membrane | 3-O-sophorosides | accumulation and transport of pollen-specific flavonol 3-O-sophorosides | (Grunewald et al., 2020) |
| AtNPF2.9 | *At1G18880* | NRT1.9 | Companion cells of root phloem | Plasma membrane | NO_3_^-^, GLS | Facilitate loading of nitrate into the root phloem | (Wang and Tsay, 2011; Nour-Eldin et al., 2012; Hunziker et al., 2020) |
| AtNPF2.10 | *At3G47960* | GTR1 | The leaf veins both of wounded and undamaged leaves | ND | GLS, NO_3_^-^, GA, JA-Ile | Transport of both methionine and tryptophan derived glucosinolates to seeds | (Nour-Eldin et al., 2012; Andersen et al., 2013; Chiba et al., 2015; Ishimaru et al., 2017; Chung et al., 2022) |
| AtNPF2.11 | *At5G62680* | GTR2 | Major veins of leaves, lateral root branching points | ND | GLS, NO_3_^-^, GA | Transport of both methionine and tryptophan derived glucosinolates to seeds | (Xu et al., 2019; Nambiar et al., 2020) |
| AtNPF2.12 | *At1G27080* | NRT1.6 | Vascular tissue of the funiculus and the silique | Plasma membrane | NO_3_^-^, GA | Delivering nitrate from maternal tissue to the developing embryo | (Almagro et al., 2008; Chiba et al., 2015; Babst et al., 2019) |
| AtNPF2.13 | *At1G69870* | NRT1.7 | The phloem of the leaf minor vein | ND | NO_3_^-^, GLS, GA, JA-Ile | Nitrate remobilization from old to young leaves | (Babst et al., 2019; Chen et al., 2020) |
| AtNPF2.14 | *At1G69860* | NA | ND | ND | GLS | ND | (Tsay et al., 2007) |
| AtNPF3.1 | *At1G68570* | Nitr | Root endodermis, major and minor veins of vascular tissue, and chloroplasts of leaf cells | ND | NO_3_^-^, NO_2_^-^,  GA, JA-Ile | Nitrite accumulation in leaves, GA accumulation | (Pike et al., 2014; David et al., 2016) |
| AtNPF4.1 | *At3G25260* | AIT3 | ND | Plasma membrane | ABA, GA, JA-Ile, GA | ND | (Kanno et al., 2012; Chiba et al., 2015; Saito et al., 2015) |
| AtNPF4.2 | *At3G25280* | AIT4 | ND | ND | ABA, GA | ND | (Kanno et al., 2012; Chiba et al., 2015) |
| AtNPF4.4 | *At1G33440* | NRT1.13 | parenchyma cells next to xylem in the petioles and the stem nodes | Plasma membrane | ND | Regulate shoot architecture and flowering time | (Chen et al., 2021) |
| AtNPF4.5 | *At1G27040* | AIT2 | ND | ND | ABA | ND | (Kanno et al., 2012) |
| AtNPF4.6 | *At1G69850* | NRT1.2/ AIT1 | Primarily in root hairs and the epidermis in both young (root tips) and mature regions of roots | ND | NO_3_^-^, ABA | Nitrate uptake in roots, ABA-mediated inhibition of seed germination | (Huang et al., 1999; Kanno et al., 2012; Chiba et al., 2015; Zhang et al., 2021) |
| AtNPF5.1 | *At1G19190* | NA | ND | ND | ABA, GA, JA-Ile | mediates ABA uptake by seed coat | (Shimizu et al., 2022) |
| AtNPF5.2 | *At5G46050* | PTR3 | ND | ND |  | Pathogen response; responses to wounding, virulent bacterial pathogens, and high NaCl concentrations | (Karim et al., 2005; Karim et al., 2007; Chiba et al., 2015) |
| AtNPF5.3 | *At5G46040* | NA | ND | ND | ABA | ND | (Chiba et al., 2015) |
| AtNPF5.5 | *At2G38100* | NA | Embryo | ND | NO_3_^-^ | Nitrogen accumulation in embryos | (Leran et al., 2015) |
| AtNPF5.6 | *At2G37900* | NA | ND | GA |  | ND | (Chiba et al., 2015) |
| AtNPF5.7 | *At3G53960* | NA | ND | ND | ABA, GA, JA-Ile | ND | (Chiba et al., 2015) |
| AtNPF5.10 | *At1G22540* | NA | Embryo | ND | NO_3_- | Nitrogen accumulation in embryos | (Leran et al., 2015; Lu et al., 2022) |
| AtNPF5.11 | *At1G72130* | NA | Root pericycle cells and xylem parenchyma cells | Tonoplast | NO_3_- | Uptake nitrate from vacuoles into cytosol | (He et al., 2017; Lu et al., 2022) |
| AtNPF5.12 | *At1G72140* | NA | Root pericycle cells and xylem parenchyma cells | Tonoplast | NO_3_- | Uptake nitrate from vacuoles into cytosol | (He et al., 2017; Lu et al., 2022) |
| AtNPF5.13 | *At1G72125* | ND | ND | ND | ND | ND | (Tsay et al., 2007) |
| AtNPF5.14 | *At1G72120* | NA | pericycle and xylem parenchyma cells | tonoplast | NO_3_^-^ | Transport of nitrate | (Lu et al., 2022) |
| AtNPF5.16 | *At1G22550* | NA | Root pericycle cells and xylem parenchyma cells | Tonoplast | NO_3_^-^ | Uptake nitrate from vacuoles into cytosol | (He et al., 2017) |
| AtNPF6.2 | *At2G26690* | NRT1.4 | Leaf petiole | ND | NO_3_^-^ | Nitrate storage in petiole | (Chiu et al., 2004) |
| AtNPF6.3 | *At1G12110* | NRT1.1 | Guard cells; Epidermis near root tips; cortex or endodermis in mature roots;  Emerging leaves and young stipules; style, stigma, and anthers | ND | NO_3_^-^, auxin |  | (Huang et al., 1996; Guo et al., 2001; Krouk et al., 2010) |
| AtNPF6.4 | *At3G21670* | NRT1.3 | Mesophyll, cortical cells of stems, pedicels, sepals, Hypocotyl | ND | ND | Polyamine resistance | (Tong et al., 2016) |
| AtNPF7.2 | *At4G21680* | NRT1.8 | Xylem parenchyma cells within the vasculature | Plasma membrane | NO_3_^-^ | Remove nitrate from xylem vessels and regulated nitrate distribution in Cd^2+^ tolerance | (Li et al., 2010; Wang et al., 2018b) |
| AtNPF7.3 | *At1G32450* | NRT1.5 | Root pericycle cells close to the xylem | Plasma membrane | NO_3_^-^ | Root xylem loading of nitrate | (Lin et al., 2008; Wang et al., 2018b; Cui et al., 2019; Du et al., 2019; Jian et al., 2019; Watanabe et al., 2020) |
| AtNPF8.1 | *At3G54140* | PTR1 | Vascular tissues throughout the plant；phloem and phloem parenchyma cells of leaves; vascular tissues of roots, sepals, and stamens | Plasma membrane | Dipeptides, JA-Ile | Uptake of di- and tripeptides | (Dietrich et al., 2004; Hammes et al., 2010; Chiba et al., 2015) |
| AtNPF8.2 | *At5G01180* | PTR5 | Pollen and ovules during early seed development | Plasma membrane | Dipeptides ABA, GA, JA-Ile | Peptide transport into germinating pollen | (Komarova et al., 2008) |
| AtNPF8.3 | *At2G02040* | PTR2 | Embryo through in situ hybridization | ND | Dipeptides | Late flowering  and altered seed development | (Cheng et al., 1988; Choi et al., 2020) |
| AtNPF8.4 | *At2G02020* | PTR4 | ND | ND | ND | ND | (Komarova et al., 2012) |
| AtNPF8.5 | *At1G62200* | NA | pericycle and xylem parenchyma cells | tonoplast | NO_3_^-^ | Transport of nitrate | (Lu et al., 2022) |
| ***Oryza sativa*** | | | | | | | |
| OsNPF2.2 | *Loc_Os12g44100* | OsPTR2 | Xylem parenchyma cells | Cell membrane | NO_3_^−^ | Root-to-shoot nitrate transport, Vascular development | (Ouyang et al., 2010; Li et al., 2015) |
| OsNPF2.4 | *Os03g48180* | NA | Root epidermis,  xylem parenchyma,  phloem companion  cells in roots and shoots | Plasma membrane | NO_3_^−^ | Absorption, long-distance transport and redistribution of nitrate | (Xia et al., 2015) |
| OsNPF4.1 | *Loc_Os11g12740* | SP1 | Phloem of young panicles | Plasma membrane | ND | Rice panicle size | (Li et al., 2009) |
| OsNPF4.5 | *Os01g0748950* | NA | mycorrhizal roots | Plasma membrane | NO_3_^-^ | Mycorrhizal symbiosis pathway nitrate uptake, promote mycorrhizal genesis | (Wang et al., 2020) |
| OsNPF5.5 | *LOC_Os10g33210* | *OsPTR3* | ND | ND | ND | ND | (Ouyang et al., 2010) |
| OsNPF5.16 | *LOC_Os01g65200* | NA | Root, tiller base and leaf sheath | ND | NO_3_^-^ | Regulate the number of tillers and yield of rice | (Wang et al., 2022) |
| OsNPF5.18 | *LOC_Os01g65120* | *qSBM1* | Root | Plasma membrane | ND | Regulation of plant height, grain number, biomass and yield in rice | (Xu et al., 2021) |
| OsNPF6.1 | *Os01g0103100* | NA | Lateral roots, root epidermal cells | Plasma membrane | NO_3_^−^ | Nitrate uptake and redistribution | (Tang et al., 2019) |
| OsNPF6.3 | *LOC_Os08g05910* | *OsNRT1.1A* | Epidermis and vascular tissues of roots, parenchyma cells of stems and leaf sheaths | vacuole membrane | NO_3_^−^ | Regulation of intracellular nitrate and ammonium | (Wang et al., 2018c) |
| OsNPF6.5 | *LOC_Os10g40600* | OsNRT1.3/  NRT1.1B | Root hairs, epidermis, vascular tissues | Cell membrane | NO_3_^−^ | Transport of nitrate | (Chen and Ma, 2015; Duan and Zhang, 2015; Hu et al., 2015; Fan et al., 2016; Gao et al., 2019; Hu et al., 2019; Zhang et al., 2019) |
| OsNPF7.1 | *LOC_Os07g41250* | OsPTR4 | Early stage to late stage during seed filling | ND | ND | Regulates tillering and grain yield | (Ouyang et al., 2010; Huang et al., 2019) |
| OsNPF7.2 | *LOC_Os02g47090* | NA | Root sclerenchyma, cortex, stele | Vacuolar membrane | NO_3_^−^ | Intracellular nitrate allocation in roots | (Hu et al., 2016; Wang et al., 2018a) |
| OsNPF7.3 | *LOC_Os04g50950* | OsPTR6 | Stems, lateral roots | Vacuolar membrane | Dipeptide | Nitrogen allocation and grain yield | (Fan et al., 2014; Fang et al., 2017) |
| OsNPF7.4 | *LOC_Os04g50940* | *OsPTR5* | ND | ND | ND | Regulates tillering and grain yield | (Huang et al., 2019) |
| OsNPF7.7 | *LOC_Os10g42870* | OsPTR10 | Panicle, stems and leaves at vegetative stage | Cell membrane and vacuolar membrane | NO_3_^−^ | Regulate tillering and nitrogen utilization positively | (Huang et al., 2018) |
| OsNPF7.9 | *LOC_Os02g46460* | NA | Primary root mature zone and lateral root; the junction of leaf and rhizome; anther; the shell of a seed | Plasma membrane | ND | Increase NO_3_^-^ transport from root to shoot | (Guan et al., 2022) |
| OsNPF8.1 | *LOC_Os01g04950* | OsPTR7 | Leaves, node, roots | Cell membrane | DMA | Dimethylarsenate accumulation in rice grain | (Tang et al., 2017) |
| OsNPF8.2 | *LOC_Os07g01070* | OsPTR1 | Drought and salt treatment induced OsPTR1 expression | ND | ND | ND | (Ouyang et al., 2010; Leran et al., 2014) |
| OsNPF8.5 | *Loc_Os03g51050* | OsPTR8 | Seeds at grain filling stage | ND | ND | ND | (Ouyang et al., 2010; Leran et al., 2014) |
| OsNPF8.9 | *Os03g13274* | OsNRT1 | Root epidermis, root hairs | ND | NO_3_^−^ | nitrate transport activity | (Lin et al., 2000) |
| OsNPF8.20 | *LOC_Os06g49250* | OsPTR9 | Leaves, panicles; young main root tips and cortical fiber cells of lateral roots | Plasma membrane | ND | NH_4_ ^+^ uptake; nitrogen assimilation, growth, and  grain yield | (Fang et al., 2013) |

**References listed in Table S1**

Almagro, A., Lin, S.H., and Tsay, Y.F. (2008). Characterization of the *Arabidopsis* nitrate transporter NRT1.6 reveals a role of nitrate in early embryo development. *Plant Cell* 20(12), 3289-3299. doi: 10.1105/tpc.107.056788

Andersen, T.G., Nour-Eldin, H.H., Fuller, V.L., Olsen, C.E., Burow, M., and Halkier, B.A. (2013). Integration of biosynthesis and long-distance transport establish organ-specific glucosinolate profiles in vegetative Arabidopsis. *Plant Cell* 25(8), 3133-3145. doi: 10.1105/tpc.113.110890

Babst, B.A., Gao, F., Acosta-Gamboa, L.M., Karve, A., Schueller, M.J., and Lorence, A. (2019). Three *NPF* genes in *Arabidopsis* are necessary for normal nitrogen cycling under low nitrogen stress. *Plant Physiol. Biochem.* 143, 1-10. doi: 10.1016/j.plaphy.2019.08.014

Chen, H.Y., Lin, S.H., Cheng, L.H., Wu, J.J., Lin, Y.C., and Tsay, Y.F. (2021). Potential transceptor AtNRT1.13 modulates shoot architecture and flowering time in a nitrate-dependent manner. *Plant Cell* 33(5), 1492-1505. doi: 10.1093/plcell/koab051

Chen, K.-E., Chen, H.-Y., Tseng, C.-S., and Tsay, Y.-F. (2020). Improving nitrogen use efficiency by manipulating nitrate remobilization in plants. *Nature Plants* 6(9), 1126-1135. doi: 10.1038/s41477-020-00758-0

Chen, Y.N., and Ho, C.H. (2022). Concept of fluorescent transport activity biosensor for the characterization of the *Arabidopsis* NPF1.3 activity of nitrate. *Sensors (Basel)* 22(3), 1198. doi: 10.3390/s22031198

Chen, Z.C., and Ma, J.F. (2015). Improving Nitrogen use efficiency in rice through enhancing root nitrate uptake mediated by a nitrate transporter, NRT1.1B. *J Genet Genomics* 42(9), 463-465. doi: 10.1016/j.jgg.2015.08.003

Cheng, C.L., Dewdney, J., Nam, H.G., den Boer, B.G., and Goodman, H.M. (1988). A new locus (NIA1) in *Arabidopsis thaliana* encoding nitrate reductase. *EMBO J* 7(11), 3309-3314. doi: 10.1002/j.1460-2075.1988.tb03201.x

Chiba, Y., Shimizu, T., Miyakawa, S., Kanno, Y., Koshiba, T., Kamiya, Y., et al. (2015). Identification of *Arabidopsis thaliana* NRT1/PTR FAMILY (NPF) proteins capable of transporting plant hormones. *J Plant Res* 128(4), 679-686. doi: 10.1007/s10265-015-0710-2

Chiu, C.C., Lin, C.S., Hsia, A.P., Su, R.C., Lin, H.L., and Tsay, Y.F. (2004). Mutation of a nitrate transporter, AtNRT1:4, results in a reduced petiole nitrate content and altered leaf development. *Plant Cell Physiol.* 45(9), 1139-1148. doi: 10.1093/pcp/pch143

Choi, M.G., Kim, E.J., Song, J.Y., Choi, S.B., Cho, S.W., Park, C.S., et al. (2020). Peptide transporter2 (PTR2) enhances water uptake during early seed germination in *Arabidopsis thaliana*. *Plant Mol Biol* 102(6), 615-624. doi: 10.1007/s11103-020-00967-3

Chung, Y.C., Cheng, H.Y., Wang, W.T., Chang, Y.J., and Lin, S.M. (2022). Transport efficiency of AtGTR1 dependents on the hydrophobicity of transported glucosinolates. *Sci Rep* 12(1), 5097. doi: 10.1038/s41598-022-09115-x

Cui, Y.N., Li, X.T., Yuan, J.Z., Wang, F.Z., Wang, S.M., and Ma, Q. (2019). Nitrate transporter NPF7.3/NRT1.5 plays an essential role in regulating phosphate deficiency responses in Arabidopsis. *Biochem Biophys Res Commun* 508(1), 314-319. doi: 10.1016/j.bbrc.2018.11.118

David, L.C., Berquin, P., Kanno, Y., Seo, M., Daniel-Vedele, F., and Ferrario-Mery, S. (2016). N availability modulates the role of NPF3.1, a gibberellin transporter, in GA-mediated phenotypes in Arabidopsis. *Planta* 244(6), 1315-1328. doi: 10.1007/s00425-016-2588-1

Dietrich, D., Hammes, U., Thor, K., Suter-Grotemeyer, M., Fluckiger, R., Slusarenko, A.J., et al. (2004). AtPTR1, a plasma membrane peptide transporter expressed during seed germination and in vascular tissue of *Arabidopsis*. *Plant J* 40(4), 488-499. doi: 10.1111/j.1365-313X.2004.02224.x

Du, X.Q., Wang, F.L., Li, H., Jing, S., Yu, M., Li, J., et al. (2019). The Transcription factor MYB59 regulates K(+)/NO3 (-) translocation in the *Arabidopsis* response to low K(+) stress. *Plant Cell* 31(3)**,** 699-714. doi: 10.1105/tpc.18.00674

Duan, D., and Zhang, H. (2015). A single SNP in *NRT1.1B* has a major impact on nitrogen use efficiency in rice. *Sci China Life Sci* 58(8), 827-828. doi: 10.1007/s11427-015-4907-3

Fan, X., Feng, H., Tan, Y., Xu, Y., Miao, Q., and Xu, G. (2016). A putative 6-transmembrane nitrate transporter OsNRT1.1b plays a key role in rice under low nitrogen. *J Integr Plant Biol* 58(6), 590-599. doi: 10.1111/jipb.12382

Fan, X., Xie, D., Chen, J., Lu, H., Xu, Y., Ma, C., et al. (2014). Over-expression of OsPTR6 in rice increased plant growth at different nitrogen supplies but decreased nitrogen use efficiency at high ammonium supply. *Plant Sci* 227, 1-11. doi: 10.1016/j.plantsci.2014.05.013

Fang, Z., Bai, G., Huang, W., Wang, Z., Wang, X., and Zhang, M. (2017). The rice peptide transporter OsNPF7.3 is induced by organic nitrogen, and contributes to nitrogen allocation and grain yield. *Front Plant Sci* 8, 1338. doi: 10.3389/fpls.2017.01338

Fang, Z., Xia, K., Yang, X., Grotemeyer, M.S., Meier, S., Rentsch, D., et al. (2013). Altered expression of the *PTR/NRT1* homologue *OsPTR9* affects nitrogen utilization efficiency, growth and grain yield in rice. *Plant Biotechnol J* 11(4), 446-458. doi: 10.1111/pbi.12031

Gao, Z., Wang, Y., Chen, G., Zhang, A., Yang, S., Shang, L., et al. (2019). The indica nitrate reductase gene *OsNR2* allele enhances rice yield potential and nitrogen use efficiency. *Nat Commun* 10(1), 5207. doi: 10.1038/s41467-019-13110-8

Grunewald, S., Marillonnet, S., Hause, G., Haferkamp, I., Neuhaus, H.E., Vess, A., et al. (2020). The Tapetal Major Facilitator NPF2.8 Is Required for Accumulation of Flavonol Glycosides on the Pollen Surface in Arabidopsis thaliana. *Plant Cell* 32(5), 1727-1748. doi: 10.1105/tpc.19.00801

Guan, Y., Liu, D.F., Qiu, J., Liu, Z.J., He, Y.N., Fang, Z.J., et al. (2022). The nitrate transporter OsNPF7.9 mediates nitrate allocation and the divergent nitrate use efficiency between indica and Japonica rice. *Plant Physiol.* 189(1), 215-229. doi: 10.1093/plphys/kiac044

Guo, F.Q., Wang, R., Chen, M., and Crawford, N.M. (2001). The *Arabidopsis* dual-affinity nitrate transporter gene *AtNRT1.1* (*CHL1*) is activated and functions in nascent organ development during vegetative and reproductive growth. *Plant Cell* 13(8), 1761-1777. doi: 10.11054/tpc.010126

Hammes, U.Z., Meier, S., Dietrich, D., Ward, J.M., and Rentsch, D. (2010). Functional properties of the *Arabidopsis* peptide transporters AtPTR1 and AtPTR5. *J Biol Chem* 285(51), 39710-39717. doi: 10.1074/jbc.M110.141457

He, Y.N., Peng, J.S., Cai, Y., Liu, D.F., Guan, Y., Yi, H.Y., et al. (2017). Tonoplast-localized nitrate uptake transporters involved in vacuolar nitrate efflux and reallocation in Arabidopsis. *Sci Rep* 7(1), 6417. doi: 10.1038/s41598-017-06744-5

Hsu, P.K., and Tsay, Y.F. (2013). Two phloem nitrate transporters, NRT1.11 and NRT1.12, are important for redistributing xylem-borne nitrate to enhance plant growth. *Plant Physiol.* 163(2), 844-856. doi: 10.1104/pp.113.226563

Hu, B., Jiang, Z., Wang, W., Qiu, Y., Zhang, Z., Liu, Y., et al. (2019). Nitrate-*NRT1.1B*-*SPX4* cascade integrates nitrogen and phosphorus signalling networks in plants. *Nat Plants* 5(4), 401-413. doi: 10.1038/s41477-019-0384-1

Hu, B., Wang, W., Ou, S., Tang, J., Li, H., Che, R., et al. (2015). Variation in *NRT1.1B* contributes to nitrate-use divergence between rice subspecies. *Nat. Genet.* 47(7), 834-838. doi: 10.1038/ng.3337

Hu, R., Qiu, D., Chen, Y., Miller, A.J., Fan, X., Pan, X., et al. (2016). Knock-down of a tonoplast localized low-affinity nitrate transporter OsNPF7.2 affects rice growth under high nitrate supply. *Front Plant Sci* 7, 1529. doi: 10.3389/fpls.2016.01529

Huang, N.C., Chiang, C.S., Crawford, N.M., and Tsay, Y.F. (1996). *CHL1* encodes a component of the low-affinity nitrate uptake system in *Arabidopsis* and shows cell type-specific expression in roots. *Plant Cell* 8(12), 2183-2191. doi: 10.1105/tpc.8.12.2183

Huang, N.C., Liu, K.H., Lo, H.J., and Tsay, Y.F. (1999). Cloning and functional characterization of an *Arabidopsis* nitrate transporter gene that encodes a constitutive component of low-affinity uptake. *Plant Cell* 11(8), 1381-1392. doi: 10.1105/tpc.11.8.1381

Huang, W., Bai, G., Wang, J., Zhu, W., Zeng, Q., Lu, K., et al. (2018). Two splicing variants of *OsNPF7.7* regulate shoot branching and nitrogen utilization efficiency in rice. *Front Plant Sci* 9, 300. doi: 10.3389/fpls.2018.00300

Huang, W., Nie, H., Feng, F., Wang, J., Lu, K., and Fang, Z. (2019). Altered expression of *OsNPF7.1* and *OsNPF7.4* differentially regulates tillering and grain yield in rice. *Plant Sci* 283**,** 23-31. doi: 10.1016/j.plantsci.2019.01.019

Hunziker, P., Ghareeb, H., Wagenknecht, L., Crocoll, C., Halkier, B.A., Lipka, V., et al. (2020). *De novo* indol-3-ylmethyl glucosinolate biosynthesis, and not long-distance transport, contributes to defence of Arabidopsis against powdery mildew. *Plant Cell Environ* 43(6), 1571-1583. doi: 10.1111/pce.13766

Ishimaru, Y., Oikawa, T., Suzuki, T., Takeishi, S., Matsuura, H., Takahashi, K., et al. (2017). GTR1 is a jasmonic acid and jasmonoyl-L-isoleucine transporter in *Arabidopsis thaliana*. *Biosci Biotechnol Biochem* 81(2), 249-255. doi: 10.1080/09168451.2016.1246174

Jian, S., Luo, J., Liao, Q., Liu, Q., Guan, C., and Zhang, Z. (2019). NRT1.1 regulates nitrate allocation and cadmium tolerance in *Arabidopsis*. *Front Plant Sci* 10, 384. doi: 10.3389/fpls.2019.00384

Kanno, Y., Hanada, A., Chiba, Y., Ichikawa, T., Nakazawa, M., Matsui, M., et al. (2012). Identification of an abscisic acid transporter by functional screening using the receptor complex as a sensor. *Proc Natl Acad Sci U S A* 109(24), 9653-9658. doi: 10.1073/pnas.1203567109

Karim, S., Holmstrom, K.O., Mandal, A., Dahl, P., Hohmann, S., Brader, G., et al. (2007). AtPTR3, a wound-induced peptide transporter needed for defence against virulent bacterial pathogens in *Arabidopsis*. *Planta* 225(6), 1431-1445. doi: 10.1007/s00425-006-0451-5

Karim, S., Lundh, D., Holmstrom, K.O., Mandal, A., and Pirhonen, M. (2005). Structural and functional characterization of AtPTR3, a stress-induced peptide transporter of *Arabidopsis*. *J Mol Model* 11(3), 226-236. doi: 10.1007/s00894-005-0257-6

Komarova, N.Y., Meier, S., Meier, A., Grotemeyer, M.S., and Rentsch, D. (2012). Determinants for Arabidopsis peptide transporter targeting to the tonoplast or plasma membrane. *Traffic* 13(8), 1090-1105. doi: 10.1111/j.1600-0854.2012.01370.x

Komarova, N.Y., Thor, K., Gubler, A., Meier, S., Dietrich, D., Weichert, A., et al. (2008). AtPTR1 and AtPTR5 transport dipeptides in planta. *Plant Physiol* 148(2)**,** 856-869. doi: 10.1104/pp.108.123844

Krouk, G., Lacombe, B., Bielach, A., Perrine-Walker, F., Malinska, K., Mounier, E., et al. (2010). Nitrate-regulated auxin transport by NRT1.1 defines a mechanism for nutrient sensing in plants. *Dev Cell* 18(6), 927-937. doi: 10.1016/j.devcel.2010.05.008

Leran, S., Garg, B., Boursiac, Y., Corratge-Faillie, C., Brachet, C., Tillard, P., et al. (2015). AtNPF5.5, a nitrate transporter affecting nitrogen accumulation in *Arabidopsis* embryo. *Sci Rep* 5**,** 7962. doi: 10.1038/srep07962

Leran, S., Varala, K., Boyer, J.-C., Chiurazzi, M., Crawford, N., Daniel-Vedele, F., et al. (2014). A unified nomenclature of NITRATE TRANSPORTER 1/PEPTIDE TRANSPORTER family members in plants. *Trends Plant Sci* 19(1), 5-9. doi: 10.1016/j.tplants.2013.08.008

Li, B., Byrt, C., Qiu, J., Baumann, U., Hrmova, M., Evrard, A., et al. (2016a). Identification of a stelar-localized transport protein that facilitates root-to-shoot transfer of chloride in *Arabidopsis*. *Plant Physiol* 170(2)**,** 1014-1029. doi: 10.1104/pp.15.01163

Li, B., Qiu, J., Jayakannan, M., Xu, B., Li, Y., Mayo, G.M., et al. (2016b). AtNPF2.5 modulates chloride (cl(-)) efflux from roots of Arabidopsis thaliana. *Front Plant Sci* 7, 2013. doi: 10.3389/fpls.2016.02013

Li, J.Y., Fu, Y.L., Pike, S.M., Bao, J., Tian, W., Zhang, Y., et al. (2010). The *Arabidopsis* nitrate transporter NRT1.8 functions in nitrate removal from the xylem sap and mediates cadmium tolerance. *Plant Cell* 22(5), 1633-1646. doi: 10.1105/tpc.110.075242

Li, S., Qian, Q., Fu, Z., Zeng, D., Meng, X., Kyozuka, J., et al. (2009). *Short panicle1* encodes a putative PTR family transporter and determines rice panicle size. *Plant J.* 58(4)**,** 592-605. doi: 10.1111/j.1365-313X.2009.03799.x

Li, Y., Ouyang, J., Wang, Y.Y., Hu, R., Xia, K., Duan, J., et al. (2015). Disruption of the rice nitrate transporter OsNPF2.2 hinders root-to-shoot nitrate transport and vascular development. *Sci Rep* 5, 9635. doi: 10.1038/srep09635

Lin, C.M., Koh, S., Stacey, G., Yu, S.M., Lin, T.Y., and Tsay, Y.F. (2000). Cloning and functional characterization of a constitutively expressed nitrate transporter gene, *OsNRT1*, from rice. *Plant Physiol* 122(2)**,** 379-388. doi: 10.1104/pp.122.2.379

Lin, S.H., Kuo, H.F., Canivenc, G., Lin, C.S., Lepetit, M., Hsu, P.K., et al. (2008). Mutation of the *Arabidopsis* NRT1.5 nitrate transporter causes defective root-to-shoot nitrate transport. *Plant Cell* 20(9), 2514-2528. doi: 10.1105/tpc.108.060244

Lu, Y.T., Liu, D.F., Wen, T.T., Fang, Z.J., Chen, S.Y., Li, H., et al. (2022). Vacuolar nitrate efflux requires multiple functional redundant nitrate transporter in *Arabidopsis thaliana*. *Front Plant Sci* 13**,** 926809. doi: 10.3389/fpls.2022.926809

Nambiar, D.M., Kumari, J., Arya, G.C., Singh, A.K., and Bisht, N.C. (2020). A cell suspension based uptake method to study high affinity glucosinolate transporters. *Plant Methods* 16**,** 75. doi: 10.1186/s13007-020-00618-0

Nour-Eldin, H.H., Andersen, T.G., Burow, M., Madsen, S.R., Jorgensen, M.E., Olsen, C.E., et al. (2012). NRT/PTR transporters are essential for translocation of glucosinolate defence compounds to seeds. *Nature* 488(7412)**,** 531-534. doi: 10.1038/nature11285

Ouyang, J., Cai, Z., Xia, K., Wang, Y., Duan, J., and Zhang, M. (2010). Identification and analysis of eight peptide transporter homologs in rice. *Plant Sci* 179(4), 374-382. doi: 10.1016/j.plantsci.2010.06.013

Pike, S., Gao, F., Kim, M.J., Kim, S.H., Schachtman, D.P., and Gassmann, W. (2014). Members of the NPF3 transporter subfamily encode pathogen-inducible nitrate/nitrite transporters in *grapevine* and *Arabidopsis*. *Plant Cell Physiol* 55(1), 162-170. doi: 10.1093/pcp/pct167

Saito, H., Oikawa, T., Hamamoto, S., Ishimaru, Y., Kanamori-Sato, M., Sasaki-Sekimoto, Y., et al. (2015). The jasmonate-responsive GTR1 transporter is required for gibberellin-mediated stamen development in *Arabidopsis*. *Nat. Commun.* 6, 6095. doi: 10.1038/ncomms7095

Segonzac, C., Boyer, J.-C., Ipotesi, E., Szponarski, W., Tillard, P., Touraine, B., et al. (2007). Nitrate efflux at the root plasma membrane: identification of an *Arabidopsis* excretion transporter. *Plant Cell* 19(11), 3760-3777. doi: 10.1105/tpc.106.048173

Shimizu, T., Kanno, Y., Watanabe, S., and Seo, M. (2022). Arabidopsis NPF5.1 regulates ABA homeostasis and seed germination by mediating ABA uptake into the seed coat. *Plant Signal Behav* 17(1), 2095488. doi: 10.1080/15592324.2022.2095488

Tang, W., Ye, J., Yao, X., Zhao, P., Xuan, W., Tian, Y., et al. (2019). Genome-wide associated study identifies NAC42-activated nitrate transporter conferring high nitrogen use efficiency in rice. *Nat. Commun.* 10(1), 5279. doi: 10.1038/s41467-019-13187-1

Tang, Z., Chen, Y., Chen, F., Ji, Y., and Zhao, F.J. (2017). OsPTR7 (OsNPF8.1), a Putative Peptide Transporter in Rice, is Involved in Dimethylarsenate Accumulation in Rice Grain. *Plant Cell Physiol* 58(5)**,** 904-913. doi: 10.1093/pcp/pcx029

Taochy, C., Gaillard, I., Ipotesi, E., Oomen, R., Leonhardt, N., Zimmermann, S., et al. (2015). The Arabidopsis root stele transporter NPF2.3 contributes to nitrate translocation to shoots under salt stress. *Plant J* 83(3), 466-479. doi: 10.1111/tpj.12901

Tong, W., Imai, A., Tabata, R., Shigenobu, S., Yamaguchi, K., Yamada, M., et al. (2016). Polyamine Resistance Is Increased by Mutations in a Nitrate Transporter Gene NRT1.3 (AtNPF6.4) in *Arabidopsis thaliana*. *Front Plant Sci* 7. doi: 10.3389/fpls.2016.00834

Tsay, Y.F., Chiu, C.C., Tsai, C.B., Ho, C.H., and Hsu, P.K. (2007). Nitrate transporters and peptide transporters. *FEBS Lett* 581(12), 2290-2300. doi: 10.1016/j.febslet.2007.04.047

Wang, J., Lu, K., Nie, H., Zeng, Q., Wu, B., Qian, J., et al. (2018a). Rice nitrate transporter OsNPF7.2 positively regulates tiller number and grain yield. *Rice (N Y)* 11(1), 12. doi: 10.1186/s12284-018-0205-6

Wang, J., Wan, R., Nie, H., Xue, S., and Fang, Z. (2022). OsNPF5.16, a nitrate transporter gene with natural variation, is essential for rice growth and yield. *Crop J* 10(2), 397-406. doi: https://doi.org/10.1016/j.cj.2021.08.005

Wang, S., Chen, A., Xie, K., Yang, X., Luo, Z., Chen, J., et al. (2020). Functional analysis of the OsNPF4.5 nitrate transporter reveals a conserved mycorrhizal pathway of nitrogen acquisition in plants. *Proc Natl Acad Sci U S A* 117(28)**,** 16649-16659. doi: 10.1073/pnas.2000926117

Wang, T., Hua, Y., Chen, M., Zhang, J., Guan, C., and Zhang, Z. (2018b). Mechanism enhancing *Arabidopsis* resistance to cadmium: the role of NRT1.5 and proton pump. *Front Plant Sci* 9, 1892. doi: 10.3389/fpls.2018.01892

Wang, W., Hu, B., Yuan, D., Liu, Y., Che, R., Hu, Y., et al. (2018c). Expression of the nitrate transporter gene *OsNRT1.1A*/*OsNPF6.3* confers high yield and early maturation in rice. *Plant Cell* 30(3), 638-651. doi: 10.1105/tpc.17.00809

Wang, Y.-Y., and Tsay, Y.-F. (2011). *Arabidopsis* Nitrate Transporter NRT1.9 Is important in phloem nitrate transport. *Plant Cell* 23(5), 1945-1957. doi: 10.1105/tpc.111.083618

Watanabe, S., Takahashi, N., Kanno, Y., Suzuki, H., Aoi, Y., Takeda-Kamiya, N., et al. (2020). The *Arabidopsis* NRT1/PTR FAMILY protein NPF7.3/NRT1.5 is an indole-3-butyric acid transporter involved in root gravitropism. *Proc Natl Acad Sci U S A* 117(49)**,** 31500-31509. doi: 10.1073/pnas.2013305117

Xia, X., Fan, X., Wei, J., Feng, H., Qu, H., Xie, D., et al. (2015). Rice nitrate transporter OsNPF2.4 functions in low-affinity acquisition and long-distance transport. *J Exp Bot* 66(1), 317-331. doi: 10.1093/jxb/eru425

Xu, D., Hunziker, P., Koroleva, O., Blennow, A., Crocoll, C., Schulz, A., et al. (2019). GTR-mediated radial import directs accumulation of defensive glucosinolates to sulfur-rich cells in the phloem cap of Arabidopsis inflorescence stem. *Mol Plant* 12(11)**,** 1474-1484. doi: 10.1016/j.molp.2019.06.008

Xu, J., Shang, L., Wang, J., Chen, M., Fu, X., He, H., et al. (2021). The *SEEDLING BIOMASS 1* allele from indica rice enhances yield performance under low-nitrogen environments. *Plant Biotechnol J* 19(9)**,** 1681-1683. doi: 10.1111/pbi.13642

Zhang, J., Liu, Y.-X., Zhang, N., Hu, B., Jin, T., Xu, H., et al. (2019). NRT1.1B is associated with root microbiota composition and nitrogen use in field-grown rice. *Nat Biotechnol* 37(6)**,** 676-+. doi: 10.1038/s41587-019-0104-4

Zhang, L., Yu, Z., Xu, Y., Yu, M., Ren, Y., Zhang, S., et al. (2021). Regulation of the stability and ABA import activity of NRT1.2/NPF4.6 by CEPR2-mediated phosphorylation in *Arabidopsis*. *Mol Plant* 14(4), 633-646. doi: 10.1016/j.molp.2021.01.009
