# Supplementary material for: Genome-wide identification and characterization of the NPF genes provide new insight into low nitrogen tolerance in Setaria
Source: Front Plant Sci. 2022 Dec 14;13:1043832. doi: 10.3389/fpls.2022.1043832 (PMC9795848; doi:10.3389/fpls.2022.1043832)
Supplement: Supplementary Figure 1 — Chromosome location and distribution analysis of the SiNPF genes. Tandem duplicated genes are linked by a red curve. [file DataSheet_1.zip › Supplementary Table 2.docx]

**Supplementary Table| 2 Primers used in this study**

| Primer | Sequences (5'-3') | Experiments |
| --- | --- | --- |
| P1 | GACGGAYGCCTGGGACTACAAG | *SiNRT1.1B* copy number analysis |
| P2 | CGTCATCCGCTCGAACAGCTC | *SiNRT1.1B* copy number analysis |
| NRT1.1B1F1 | ccgGAATTCACACACTCACACAACCTC | Construction of the pC1300-SiNRT1.1B1 vector |
| NRT1.1B1R1 | ccgcaCTCGAGTTCGAACCATGGTTAATTAACTAATTGCACAGATGGGGTC | Construction of the pC1300-SiNRT1.1B1 vector |
| NRT1.1B1F2 | GACAATGTGGTGCTAATGATGG | Construction of the pC1300-SiNRT1.1B1 vector |
| NRT1.1B1R2 | cgagTTCGAACCATGGCCATATATACGGATCTATATCGTC | Construction of the pC1300-SiNRT1.1B1 vector |
| NRT1.1B2F1 | ttgaattcgggcccagaTCTTGGAGCATTTGGTGTCTG | Construction of the pC1300-SiNRT1.1B2 vector |
| NRT1.1B2R1 | acctctagattaattaaccaTGGGGATGCGAAATGTTGCGG | Construction of the pC1300-SiNRT1.1B2 vector |
| NRT1.1B2F2 | AATACATCCACCTAGGCGTGTC | Construction of the pC1300-SiNRT1.1B2 vector |
| NRT1.1B2R2 | ctcgacccatggCTACATGTACACTGCATGCTAACTC | Construction of the pC1300-SiNRT1.1B2 vector |
| AtACT7F | CCATGTATGTTGCCATTCAGG | Internal reference for RT-PCR of *atnrt1.1* mutant harboring the SiNRT1.1B1/2 gene |
| AtACT7B | CACATCTGTTGGAAGGTGCTG | Internal reference for RT-PCR of *atnrt1.1* mutant harboring the SiNRT1.1B1/2 gene |
| SiNRT1.1B1F | CGCATCCTTCTCTATTCTTCTTG | RT-PCR of SiNRT1.1B1 |
| SiNRT1.1B1R | TATCTACATTTCTTTACCGCCAC | RT-PCR of SiNRT1.1B1 |
| SiNRT1.1B2F | CCTTACATGCATGTTGGAAGTTG | RT-PCR of SiNRT1.1B2 |
| SiNRT1.1B2R | CTACATGTACACTGCATGCTAACTC | RT-PCR of SiNRT1.1B2 |
