# Supplementary figures and images for: Genome-wide identification and characterization of the NPF genes provide new insight into low nitrogen tolerance in Setaria
Source: Front Plant Sci. 2022 Dec 14;13:1043832. doi: 10.3389/fpls.2022.1043832 (PMC9795848; doi:10.3389/fpls.2022.1043832)

chr1

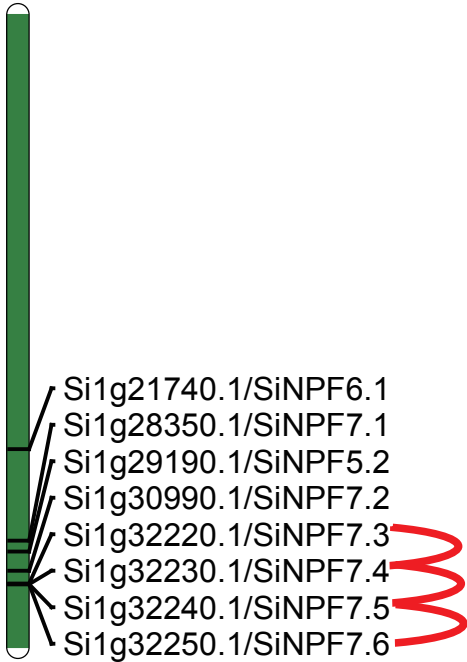

chr2

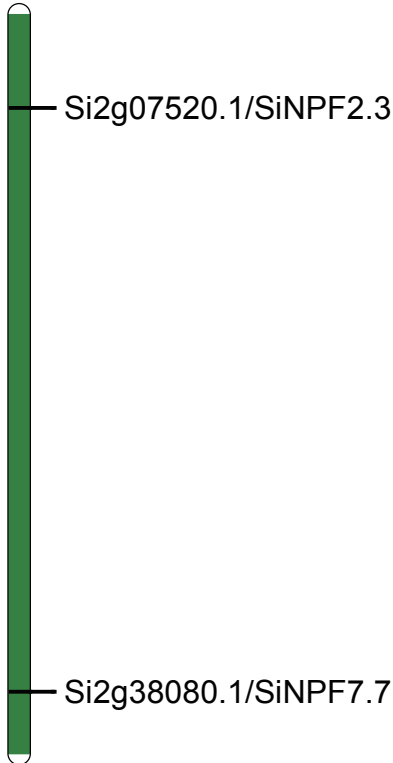

chr3

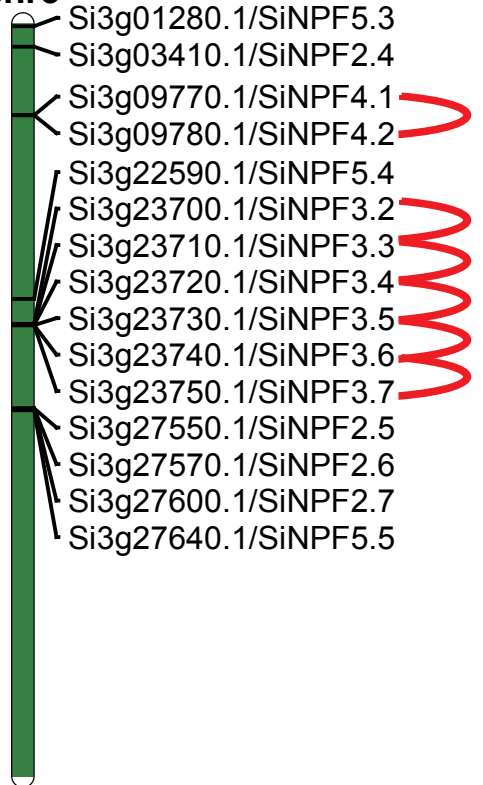

chr4

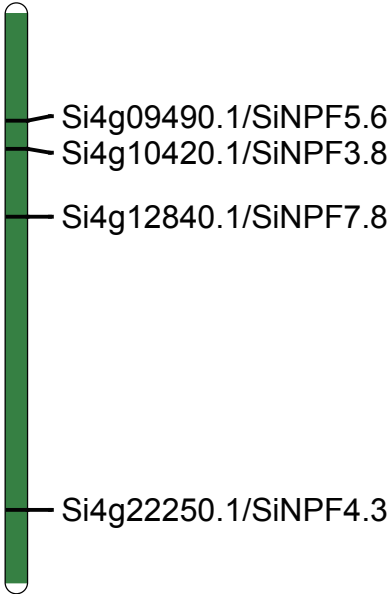

chr5

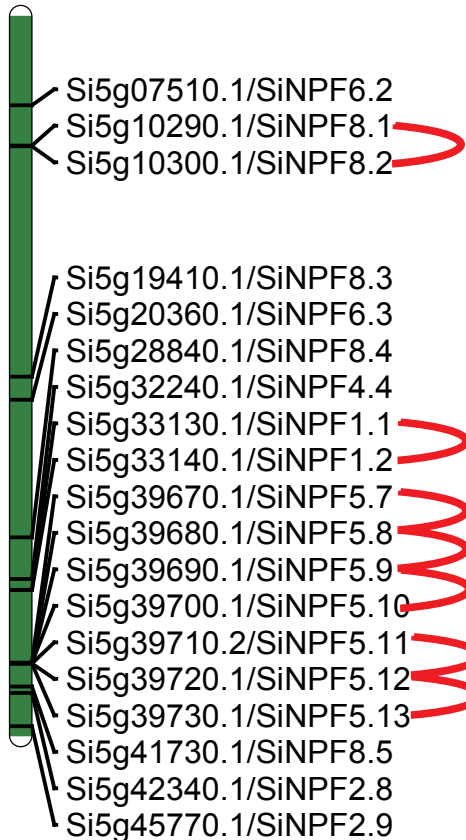

chr6

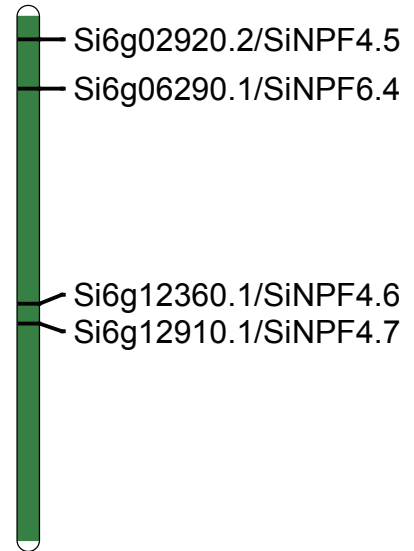

chr7

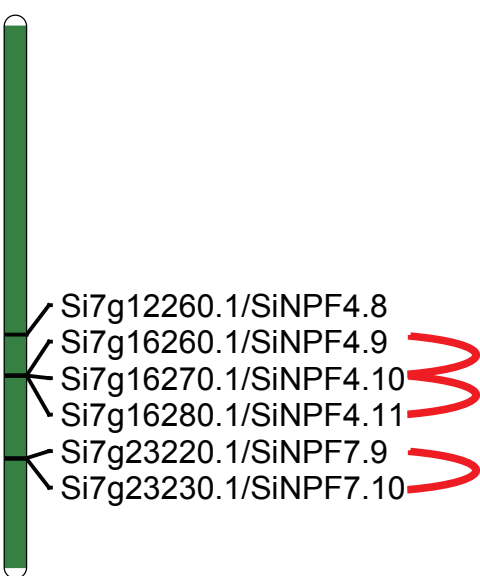

chr8

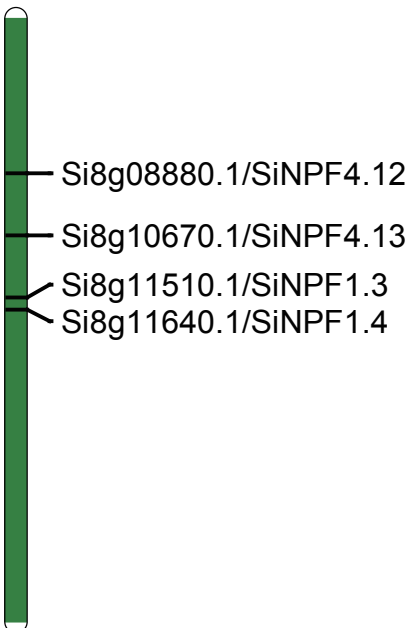

chr9

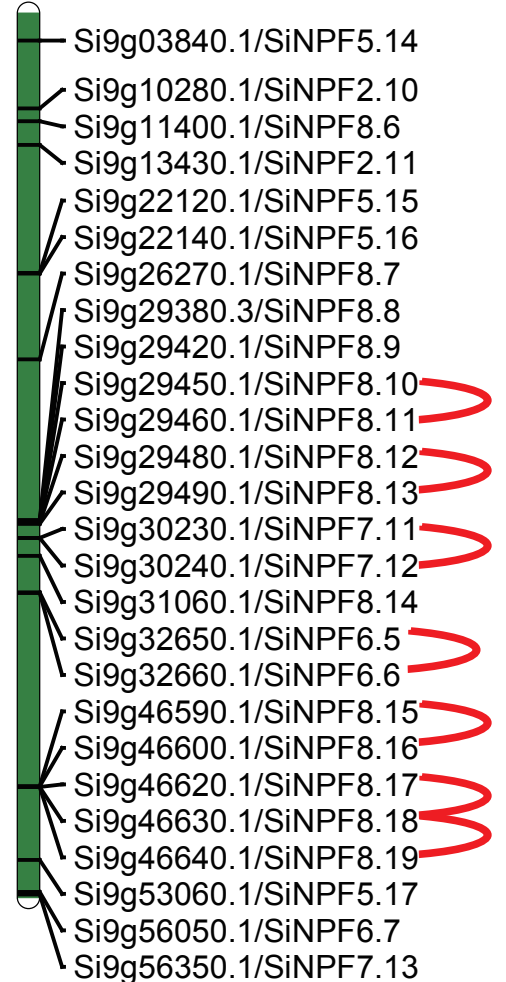

Supplement: Supplementary Figure 1 — Chromosome location and distribution analysis of the SiNPF genes. Tandem duplicated genes are linked by a red curve. [file DataSheet_1.zip › Supplementary Figure 1.pdf]

chr1

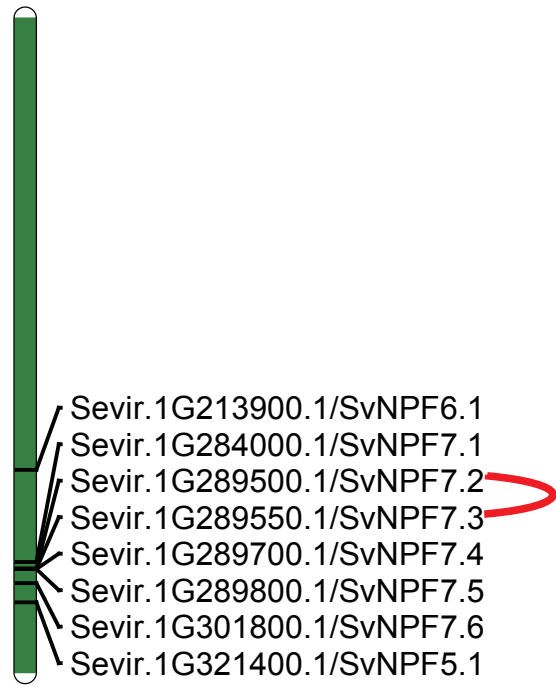

chr2

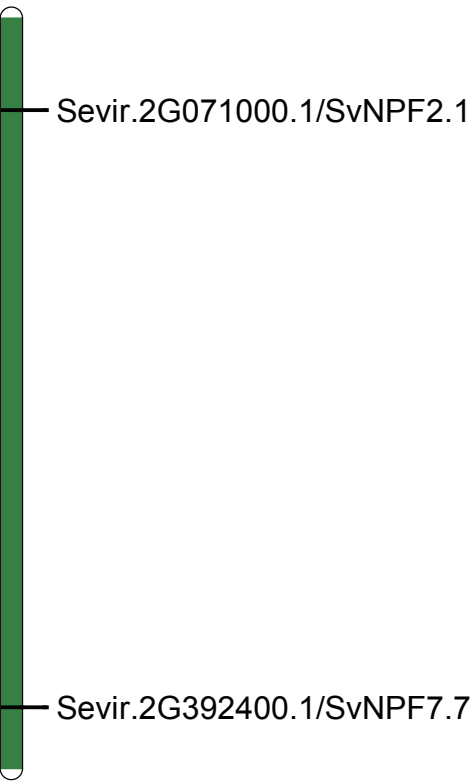

chr3

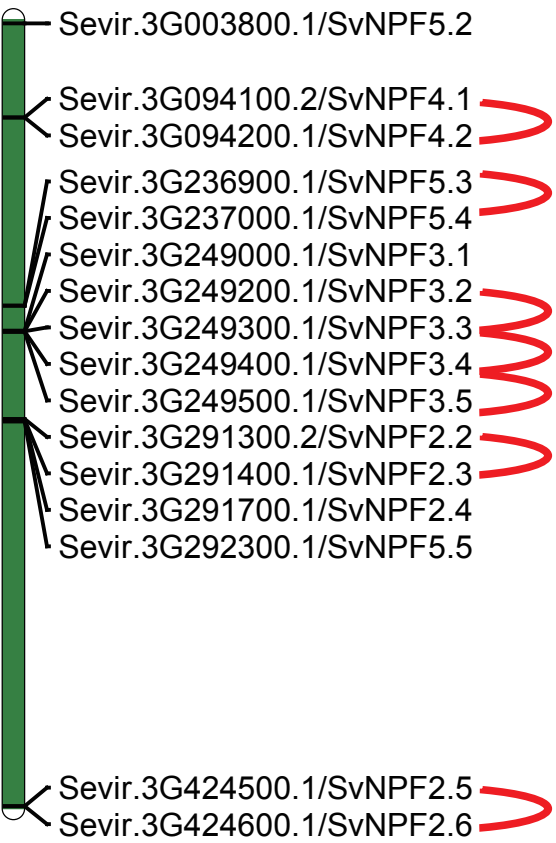

chr4

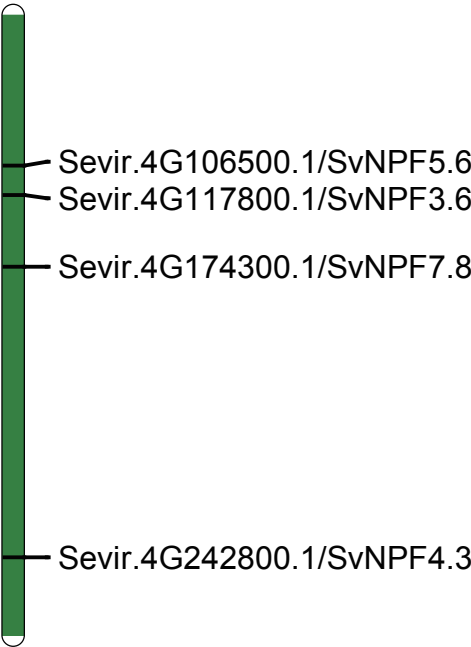

chr5

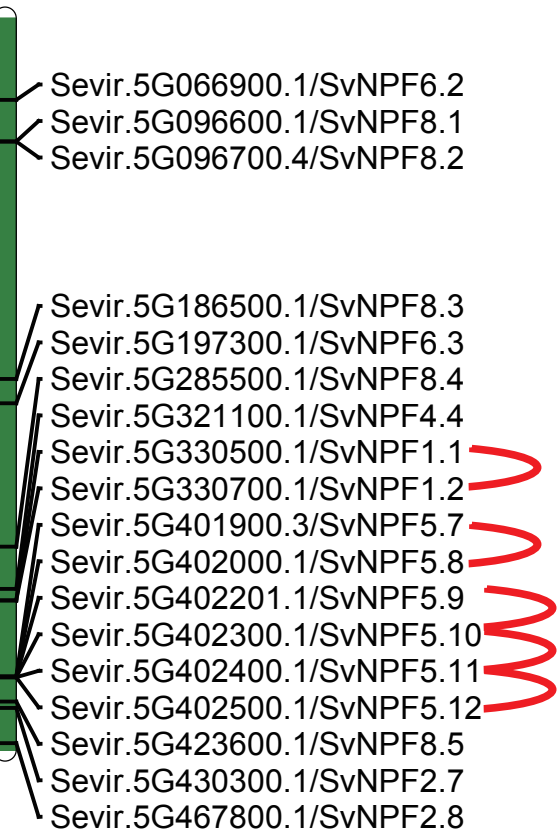

chr6

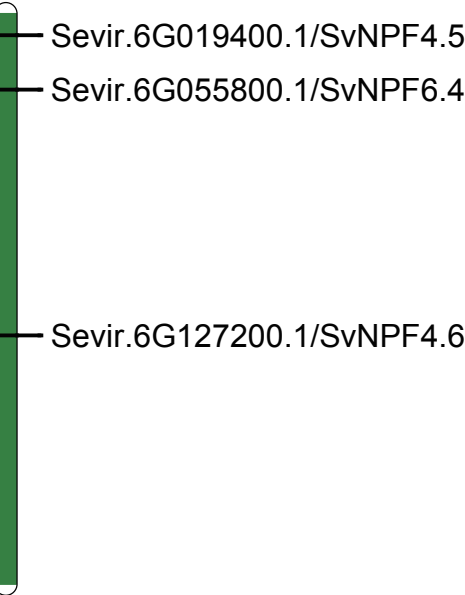

chr7

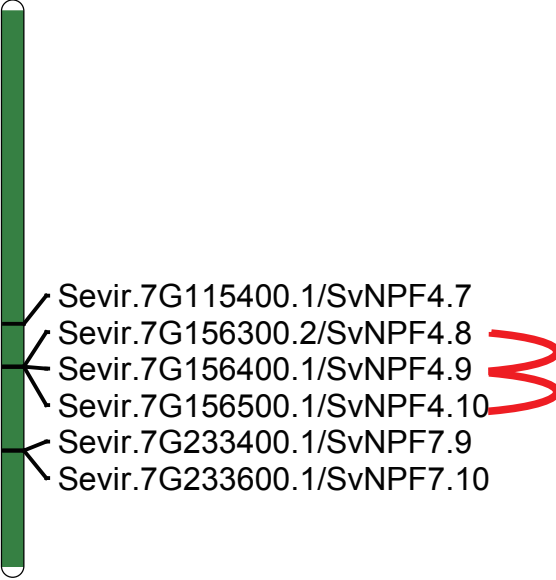

chr8

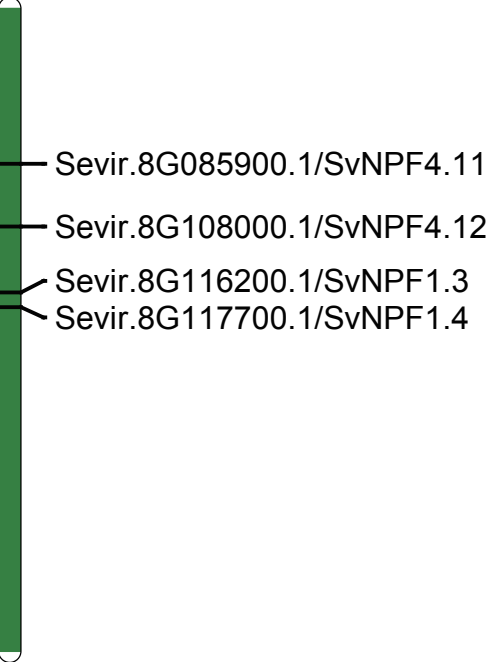

chr9

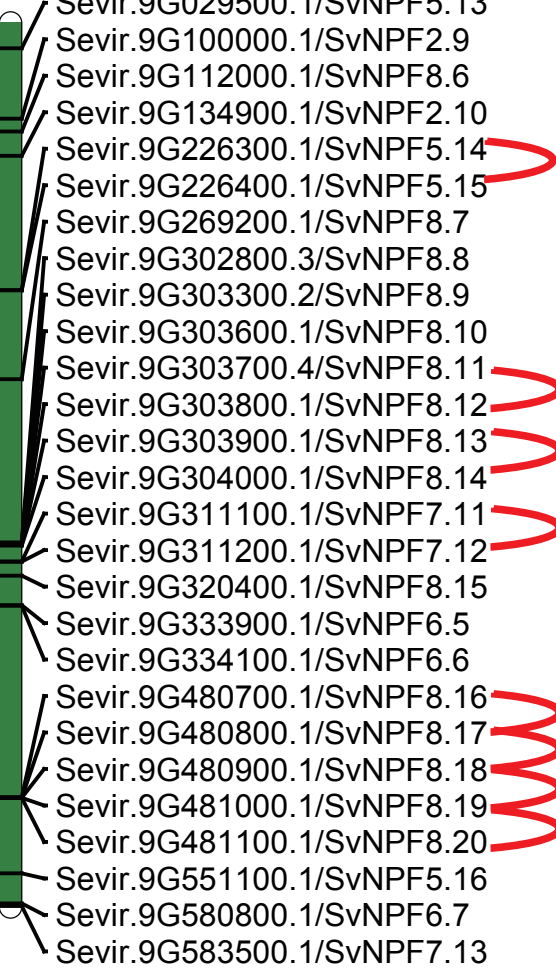

Supplement: Supplementary Figure 1 — Chromosome location and distribution analysis of the SiNPF genes. Tandem duplicated genes are linked by a red curve. [file DataSheet_1.zip › Supplementary Figure 2.pdf]

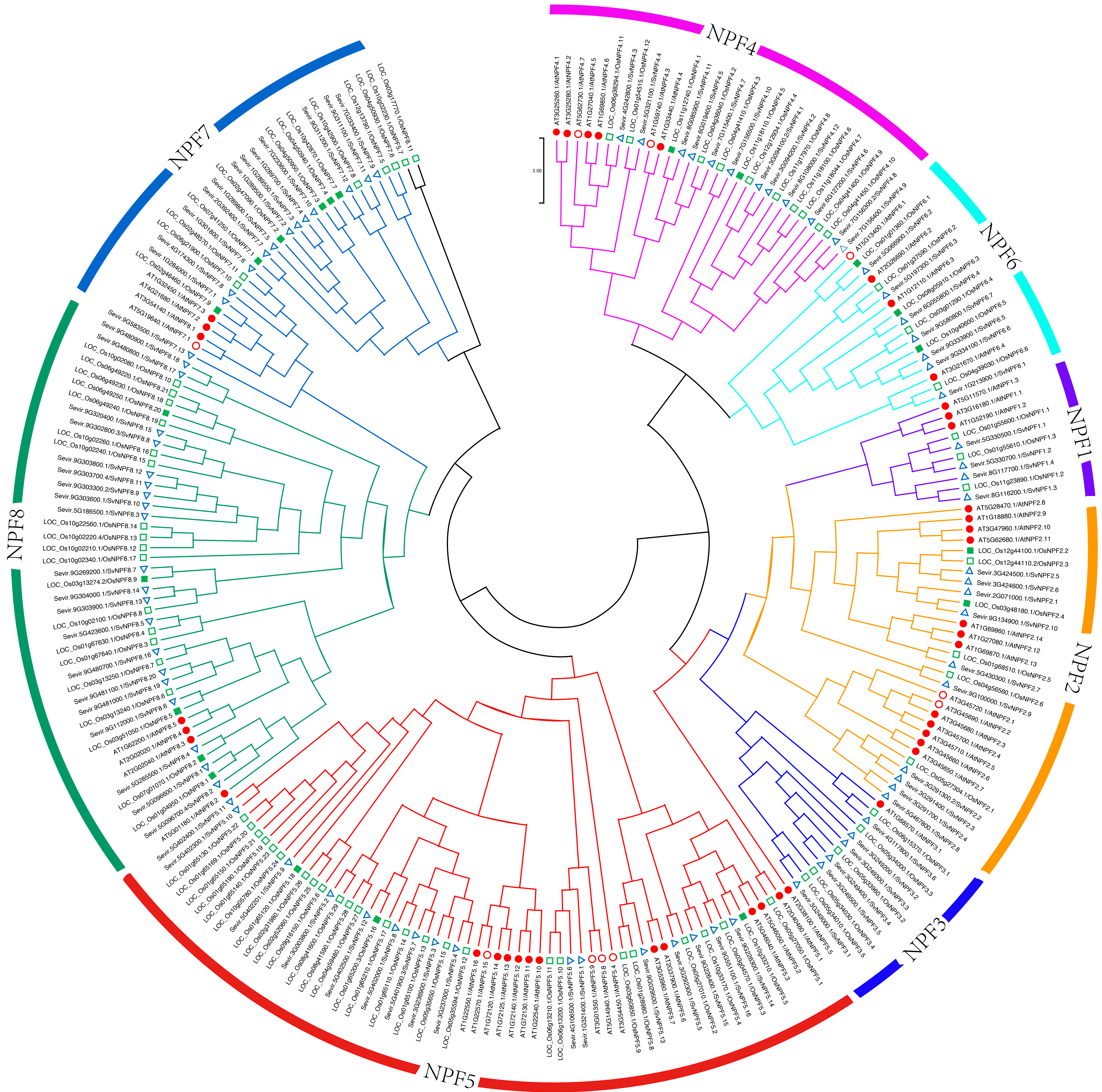

Supplement: Supplementary Figure 1 — Chromosome location and distribution analysis of the SiNPF genes. Tandem duplicated genes are linked by a red curve. [file DataSheet_1.zip › Supplementary Figure 3.pdf]

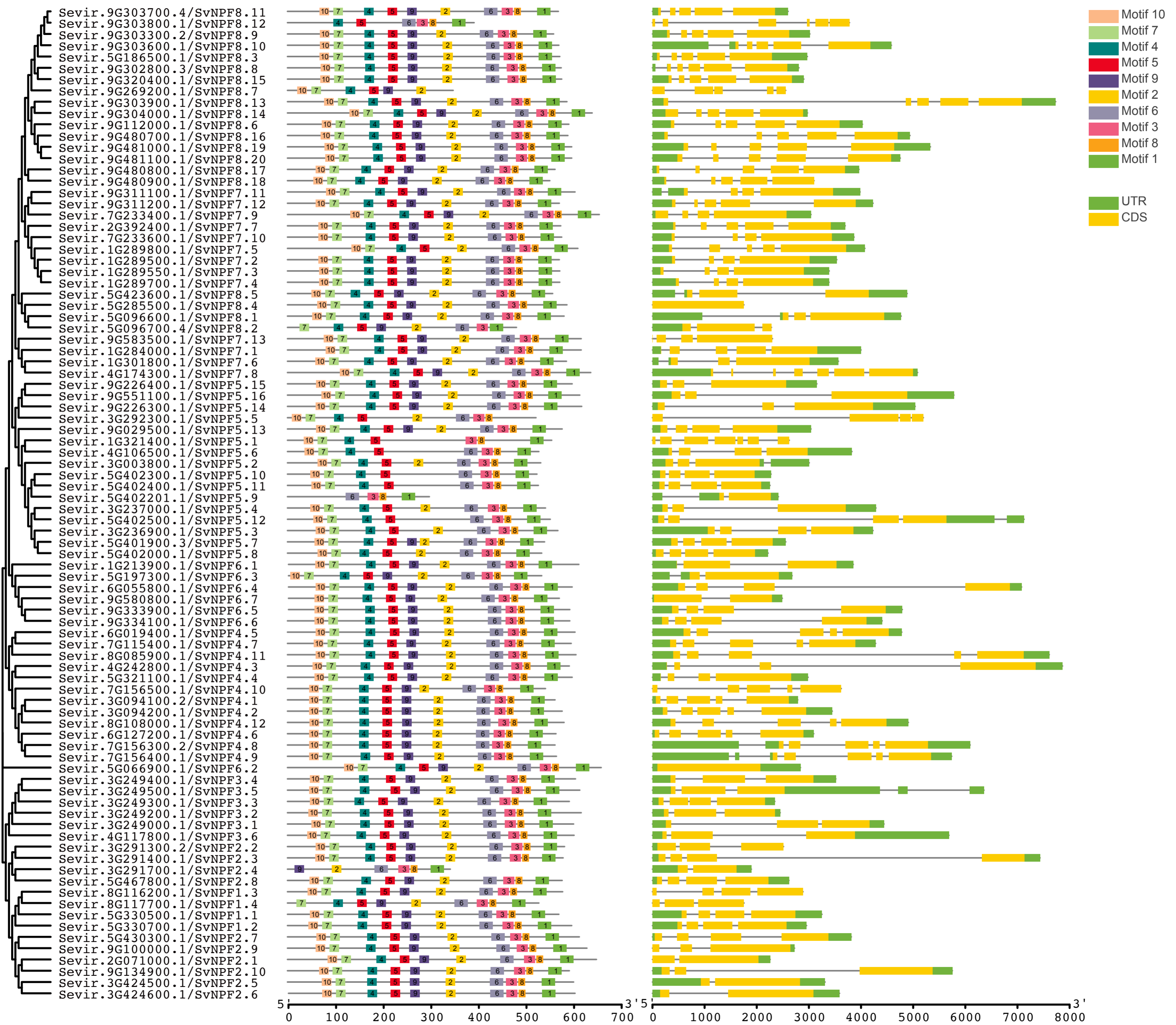

Supplement: Supplementary Figure 1 — Chromosome location and distribution analysis of the SiNPF genes. Tandem duplicated genes are linked by a red curve. [file DataSheet_1.zip › Supplementary Figure 4.pdf]
