# Supplementary material for: Genome-wide identification and characterization of the NPF genes provide new insight into low nitrogen tolerance in Setaria
Source: Front Plant Sci. 2022 Dec 14;13:1043832. doi: 10.3389/fpls.2022.1043832 (PMC9795848; doi:10.3389/fpls.2022.1043832)
Supplement: Supplementary Figure 1 — Chromosome location and distribution analysis of the SiNPF genes. Tandem duplicated genes are linked by a red curve. [file DataSheet_1.zip › Supplementary Data.zip!/Supplementary Data 3/Si0g05510.1.pdf]

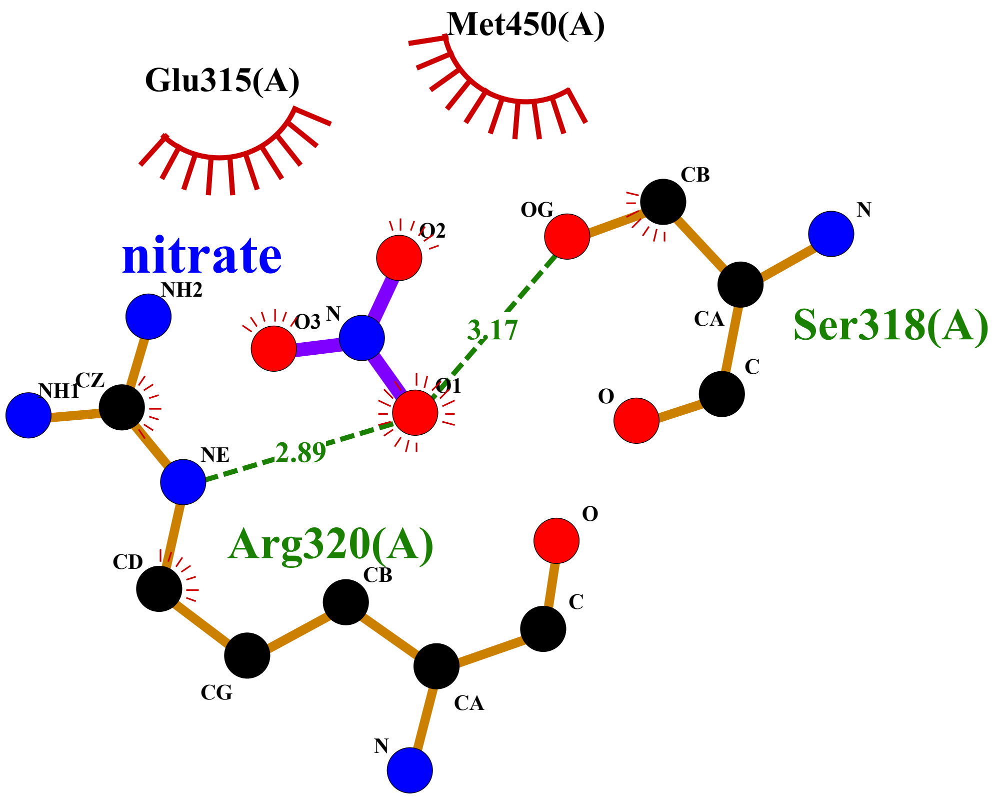

Met450(A)
Glu315(A)
nitrate
NH2
O2
OG
CB
N
O3
N
3.17
CA
Ser318(A)
NH1
CZ
O1
C
O
NE
2.89
Arg320(A)
O
CD
CB
C
CG
CA
N

Si0g05510.1
