# Supplementary material for: Genome-wide identification and characterization of the NPF genes provide new insight into low nitrogen tolerance in Setaria
Source: Front Plant Sci. 2022 Dec 14;13:1043832. doi: 10.3389/fpls.2022.1043832 (PMC9795848; doi:10.3389/fpls.2022.1043832)
Supplement: Supplementary Figure 1 — Chromosome location and distribution analysis of the SiNPF genes. Tandem duplicated genes are linked by a red curve. [file DataSheet_1.zip › Supplementary Data.zip!/Supplementary Data 3/Si0g08150.1.pdf]

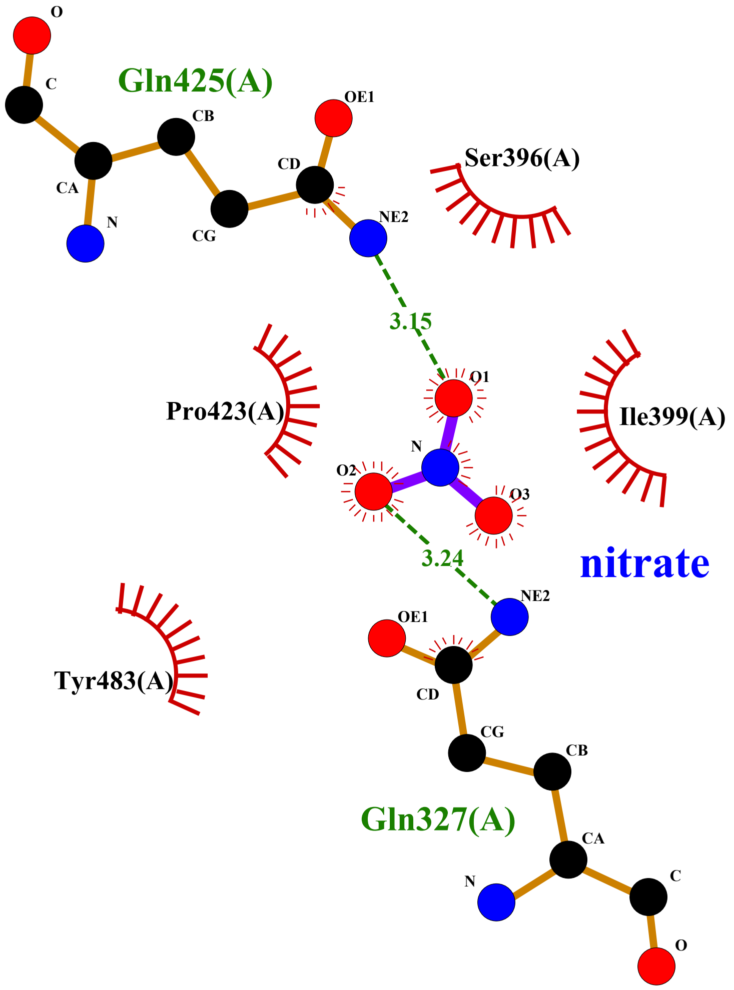

Gln425(A)
O
C
CA
N
CB
CG
CD
OE1
NE2
Ser396(A)
3.15
Pro423(A)
Ile399(A)
O1
N
O2
O3
3.24
nitrate
NE2
OE1
CD
Tyr483(A)
CG
CB
Gln327(A)
CA
N
C
O

Si0g08150.1
