# Supplementary material for: Genome-wide identification and characterization of the NPF genes provide new insight into low nitrogen tolerance in Setaria
Source: Front Plant Sci. 2022 Dec 14;13:1043832. doi: 10.3389/fpls.2022.1043832 (PMC9795848; doi:10.3389/fpls.2022.1043832)
Supplement: Supplementary Figure 1 — Chromosome location and distribution analysis of the SiNPF genes. Tandem duplicated genes are linked by a red curve. [file DataSheet_1.zip › Supplementary Data.zip!/Supplementary Data 3/Si0g08180.1.pdf]

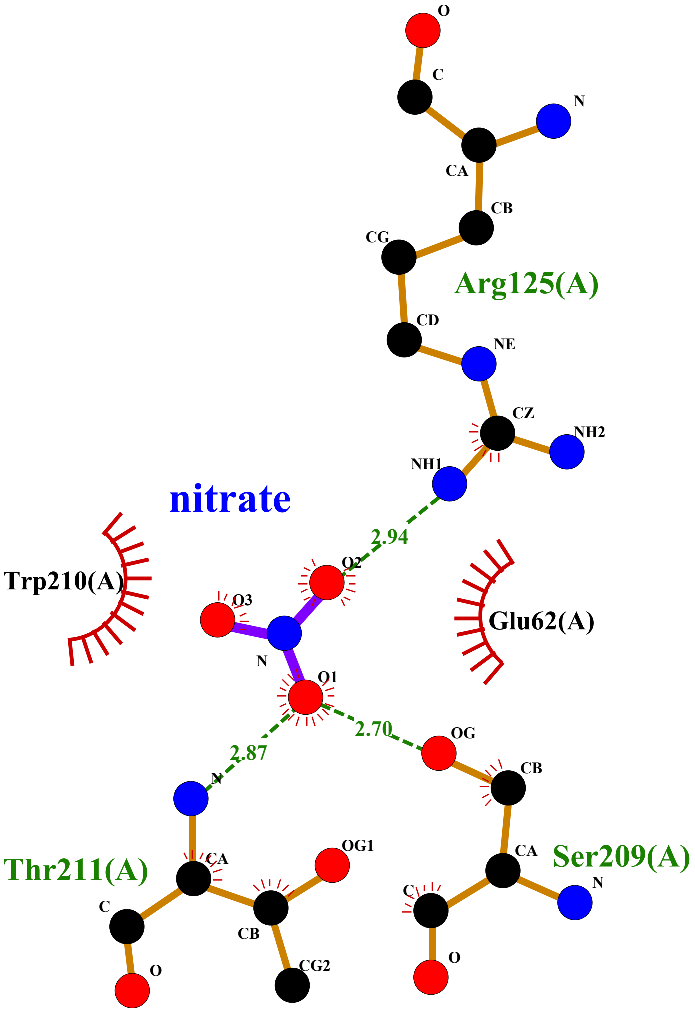
O
C
N
CA
CB
CG
Arg125(A)
CD
NE
CZ
NH2
NH1
nitrate
2.94
Trp210(A)
O2
O3
Glu62(A)
N
O1
2.70
OG
2.87
CB
N
OG1
CA
Ser209(A)
Thr211(A)
CA
C
N
C
CB
CG2
O
O

Si0g08180.1
