# Supplementary material for: Genome-wide identification and characterization of the NPF genes provide new insight into low nitrogen tolerance in Setaria
Source: Front Plant Sci. 2022 Dec 14;13:1043832. doi: 10.3389/fpls.2022.1043832 (PMC9795848; doi:10.3389/fpls.2022.1043832)
Supplement: Supplementary Figure 1 — Chromosome location and distribution analysis of the SiNPF genes. Tandem duplicated genes are linked by a red curve. [file DataSheet_1.zip › Supplementary Data.zip!/Supplementary Data 3/Si0g08230.1.pdf]

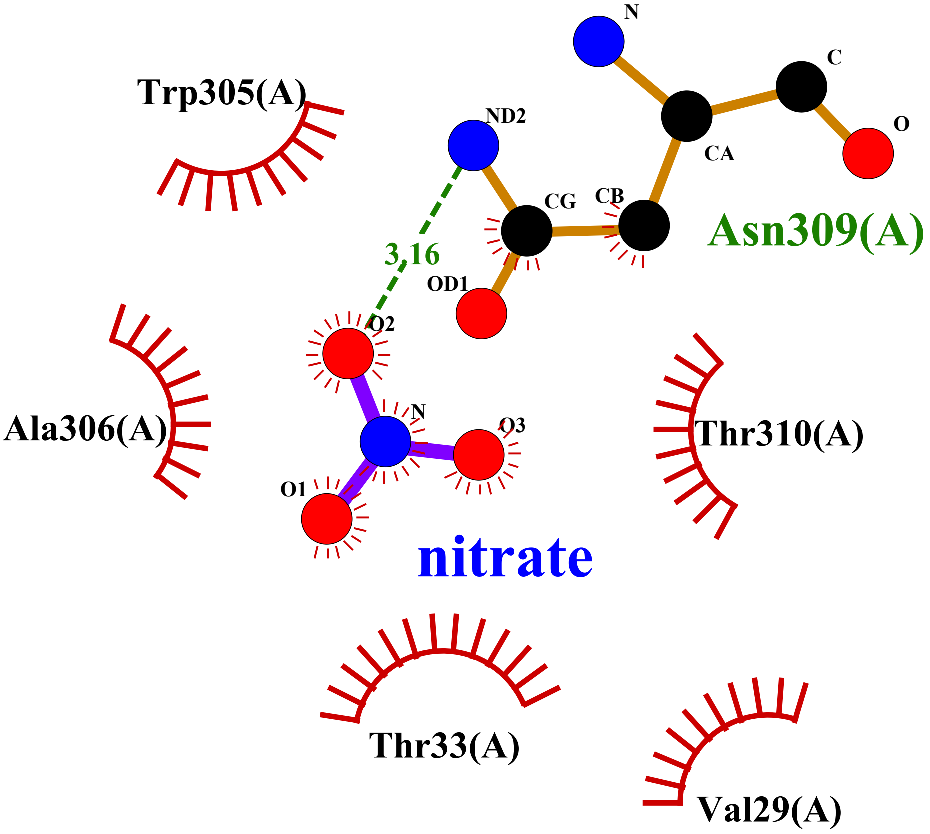

N
C
Trp305(A)
ND2
O
CA
CG
CB
Asn309(A)
3.16
OD1
O2
Ala306(A)
N
O3
Thr310(A)
O1
nitrate
Thr33(A)
Val29(A)

Si0g08230.1
