# Supplementary material for: Genome-wide identification and characterization of the NPF genes provide new insight into low nitrogen tolerance in Setaria
Source: Front Plant Sci. 2022 Dec 14;13:1043832. doi: 10.3389/fpls.2022.1043832 (PMC9795848; doi:10.3389/fpls.2022.1043832)
Supplement: Supplementary Figure 1 — Chromosome location and distribution analysis of the SiNPF genes. Tandem duplicated genes are linked by a red curve. [file DataSheet_1.zip › Supplementary Data.zip!/Supplementary Data 3/Si1g21740.1.pdf]

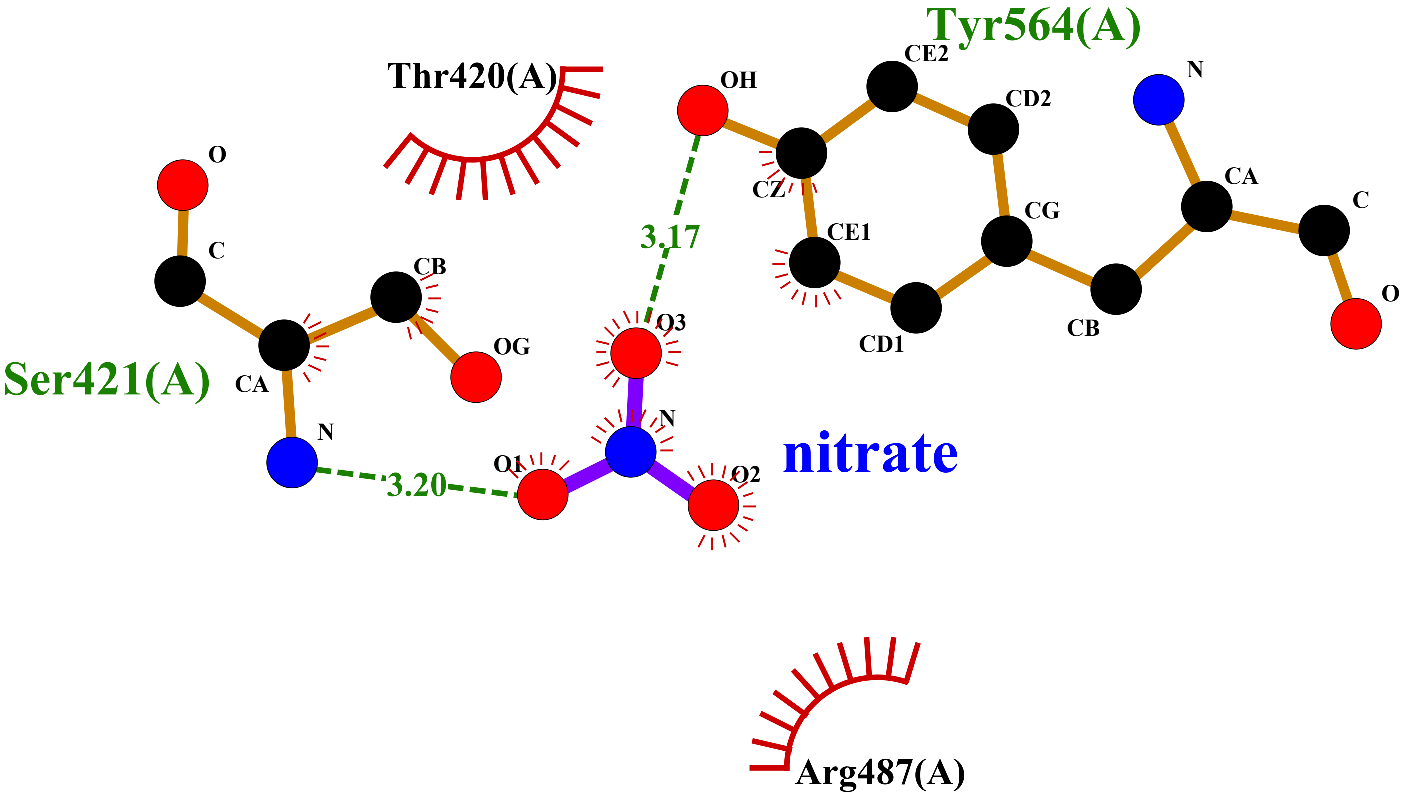
Thr420(A)
Tyr564(A)
OH
CE2
CD2
N
CA
C
O
CZ
CE1
CG
CD1
CB
Ser421(A)
O
C
CB
OG
CA
N
3.17
3.20
O3
N
O1
O2
nitrate
Arg487(A)

Si1g21740.1
