# Supplementary material for: Genome-wide identification and characterization of the NPF genes provide new insight into low nitrogen tolerance in Setaria
Source: Front Plant Sci. 2022 Dec 14;13:1043832. doi: 10.3389/fpls.2022.1043832 (PMC9795848; doi:10.3389/fpls.2022.1043832)
Supplement: Supplementary Figure 1 — Chromosome location and distribution analysis of the SiNPF genes. Tandem duplicated genes are linked by a red curve. [file DataSheet_1.zip › Supplementary Data.zip!/Supplementary Data 3/Si1g28350.1.pdf]

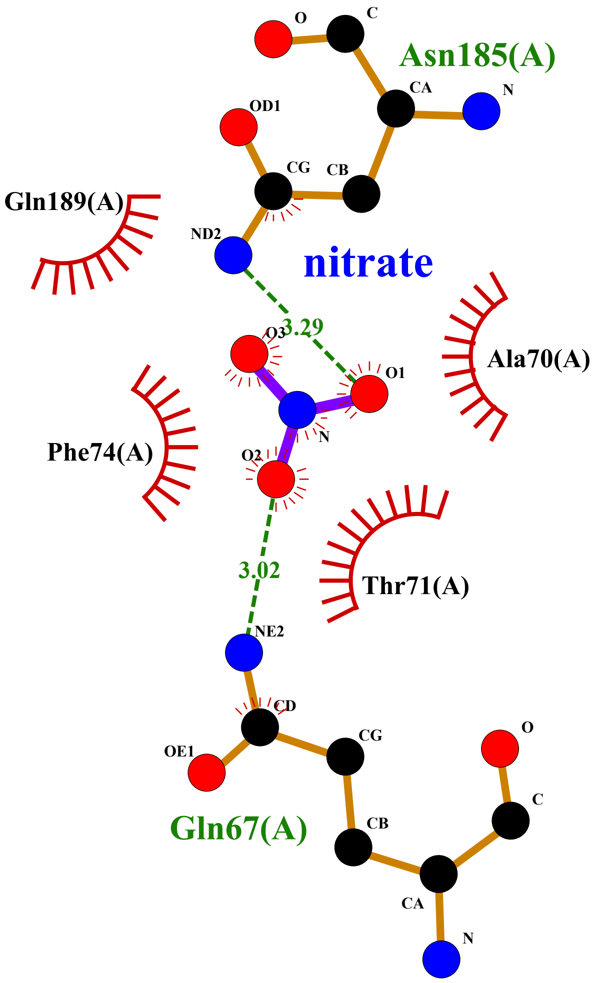

O
C
Asn185(A)
CA
N
OD1
CG
CB
Gln189(A)
ND2
nitrate
3.29
O3
O1
Ala70(A)
N
Phe74(A)
O2
3.02
Thr71(A)
NE2
CD
OE1
CG
O
C
Gln67(A)
CB
CA
N

Si1g28350.1
