# Supplementary material for: Genome-wide identification and characterization of the NPF genes provide new insight into low nitrogen tolerance in Setaria
Source: Front Plant Sci. 2022 Dec 14;13:1043832. doi: 10.3389/fpls.2022.1043832 (PMC9795848; doi:10.3389/fpls.2022.1043832)
Supplement: Supplementary Figure 1 — Chromosome location and distribution analysis of the SiNPF genes. Tandem duplicated genes are linked by a red curve. [file DataSheet_1.zip › Supplementary Data.zip!/Supplementary Data 3/Si1g29190.1.pdf]

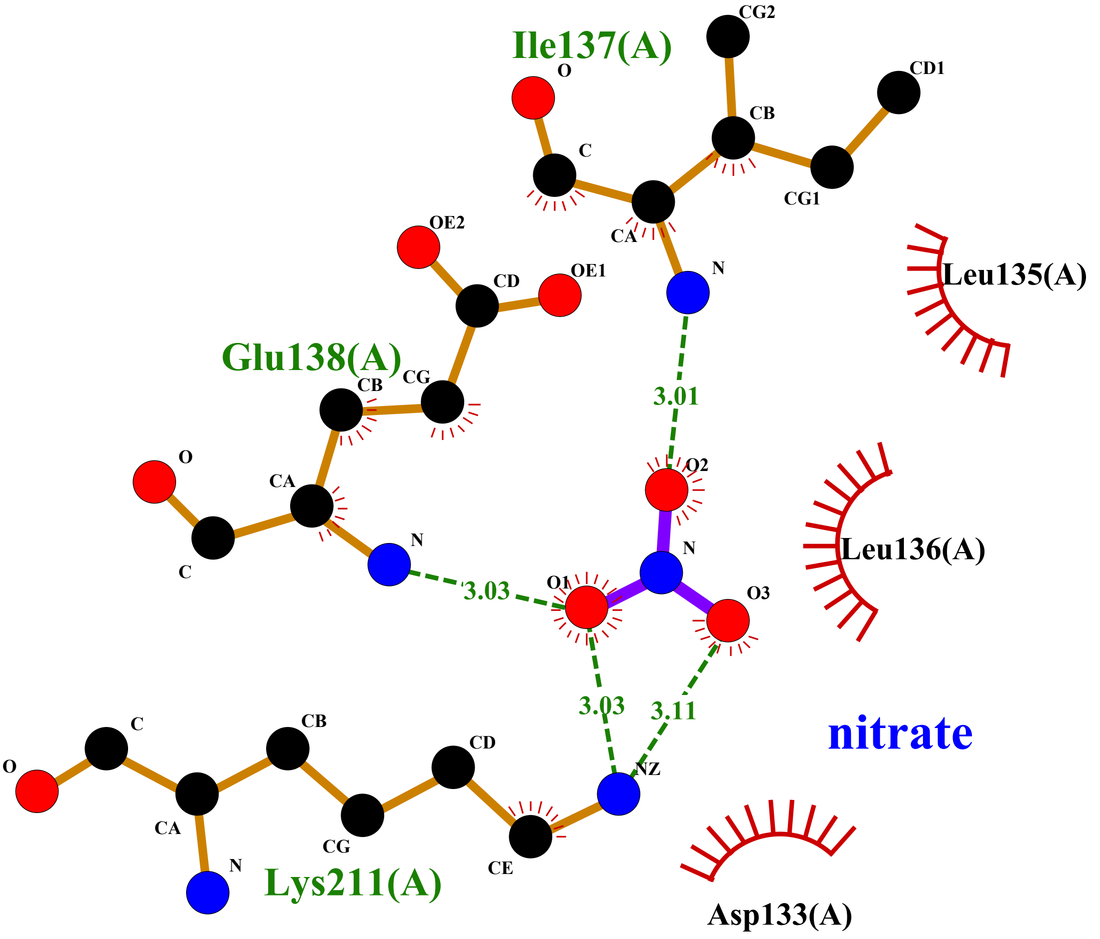
Ile137(A)
O
C
CA
N
CB
CG2
CG1
CD1
Leu135(A)
Glu138(A)
OE2
CD
OE1
CG
CB
CA
O
C
N
3.01
3.03
O2
N
O1
O3
Leu136(A)
3.03
3.11
nitrate
O
C
CA
CB
CG
CD
CE
NZ
N
Lys211(A)
Asp133(A)

Si1g29190.1
