# Supplementary material for: Genome-wide identification and characterization of the NPF genes provide new insight into low nitrogen tolerance in Setaria
Source: Front Plant Sci. 2022 Dec 14;13:1043832. doi: 10.3389/fpls.2022.1043832 (PMC9795848; doi:10.3389/fpls.2022.1043832)
Supplement: Supplementary Figure 1 — Chromosome location and distribution analysis of the SiNPF genes. Tandem duplicated genes are linked by a red curve. [file DataSheet_1.zip › Supplementary Data.zip!/Supplementary Data 3/Si1g30990.1.pdf]

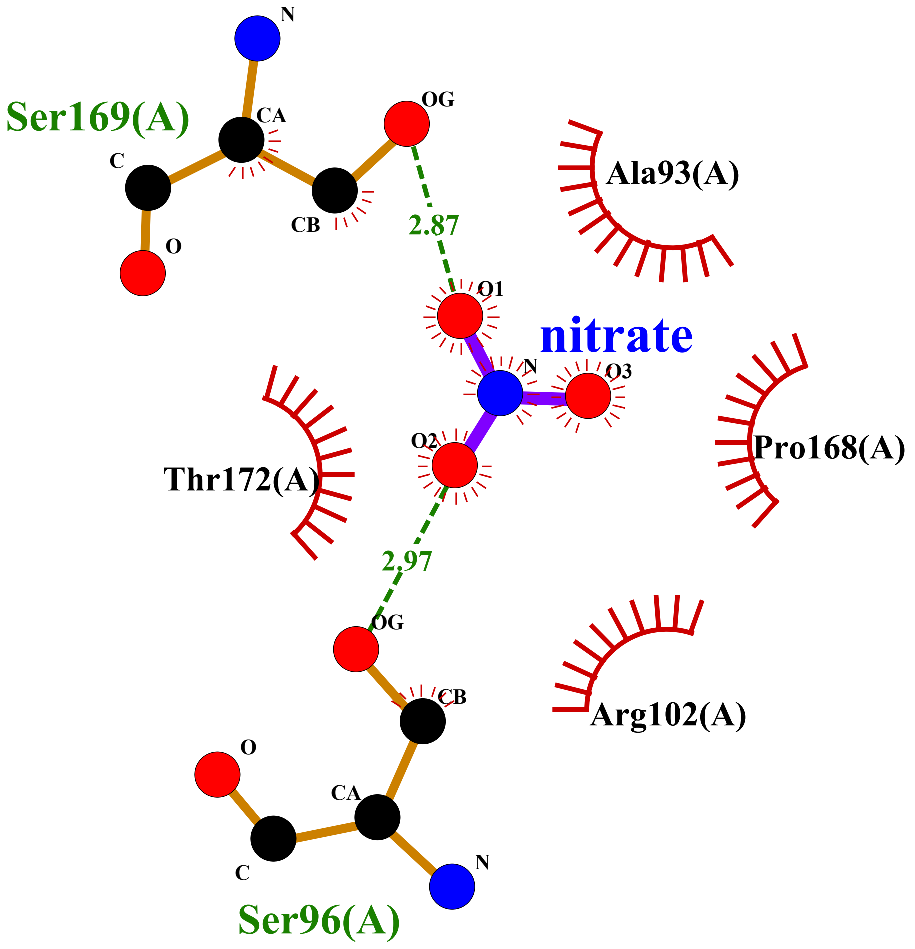
Ser169(A)
N
CA
C
O
OG
CB
2.87
Ala93(A)
O1
nitrate
N
O3
O2
Thr172(A)
Pro168(A)
2.97
OG
CB
Arg102(A)
O
CA
C
N
Ser96(A)

Si1g30990.1
