# Supplementary material for: Genome-wide identification and characterization of the NPF genes provide new insight into low nitrogen tolerance in Setaria
Source: Front Plant Sci. 2022 Dec 14;13:1043832. doi: 10.3389/fpls.2022.1043832 (PMC9795848; doi:10.3389/fpls.2022.1043832)
Supplement: Supplementary Figure 1 — Chromosome location and distribution analysis of the SiNPF genes. Tandem duplicated genes are linked by a red curve. [file DataSheet_1.zip › Supplementary Data.zip!/Supplementary Data 3/Si1g32220.1.pdf]

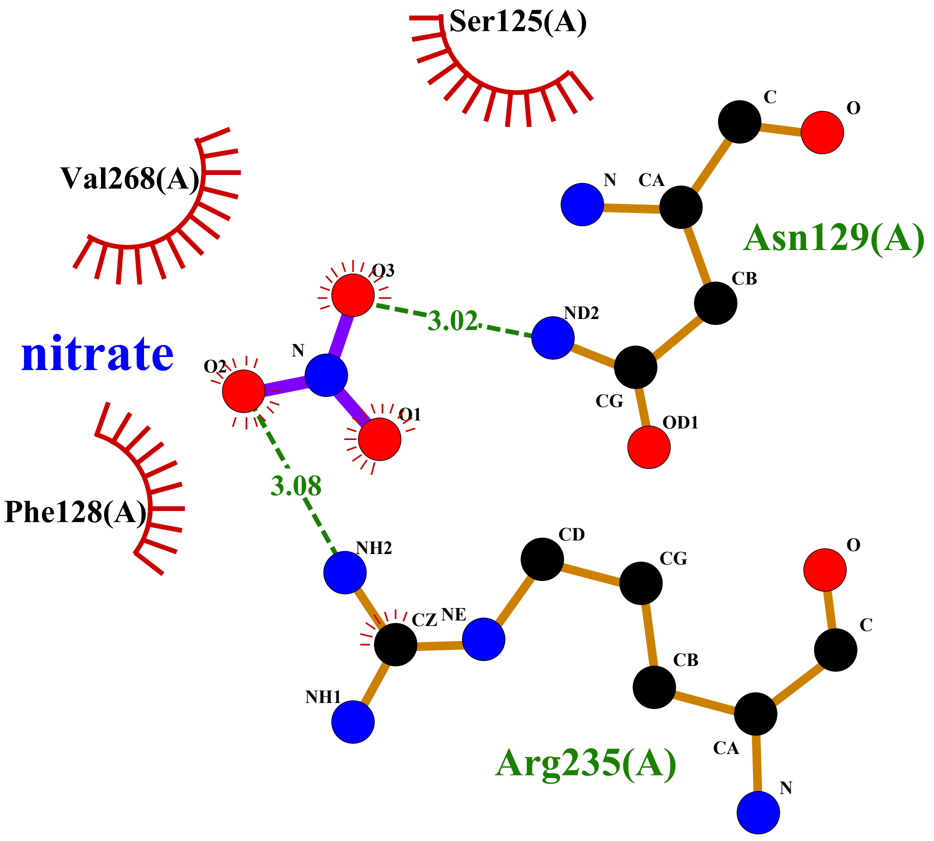
Ser125(A)
C
O
Val268(A)
N
CA
Asn129(A)
O3
CB
3.02
ND2
nitrate
O2
N
O1
CG
OD1
3.08
Phe128(A)
NH2
CD
CG
O
CZ
NE
C
CB
NH1
CA
Arg235(A)
N

Si1g32220.1
