# Supplementary material for: Genome-wide identification and characterization of the NPF genes provide new insight into low nitrogen tolerance in Setaria
Source: Front Plant Sci. 2022 Dec 14;13:1043832. doi: 10.3389/fpls.2022.1043832 (PMC9795848; doi:10.3389/fpls.2022.1043832)
Supplement: Supplementary Figure 1 — Chromosome location and distribution analysis of the SiNPF genes. Tandem duplicated genes are linked by a red curve. [file DataSheet_1.zip › Supplementary Data.zip!/Supplementary Data 3/Si1g32240.1.pdf]

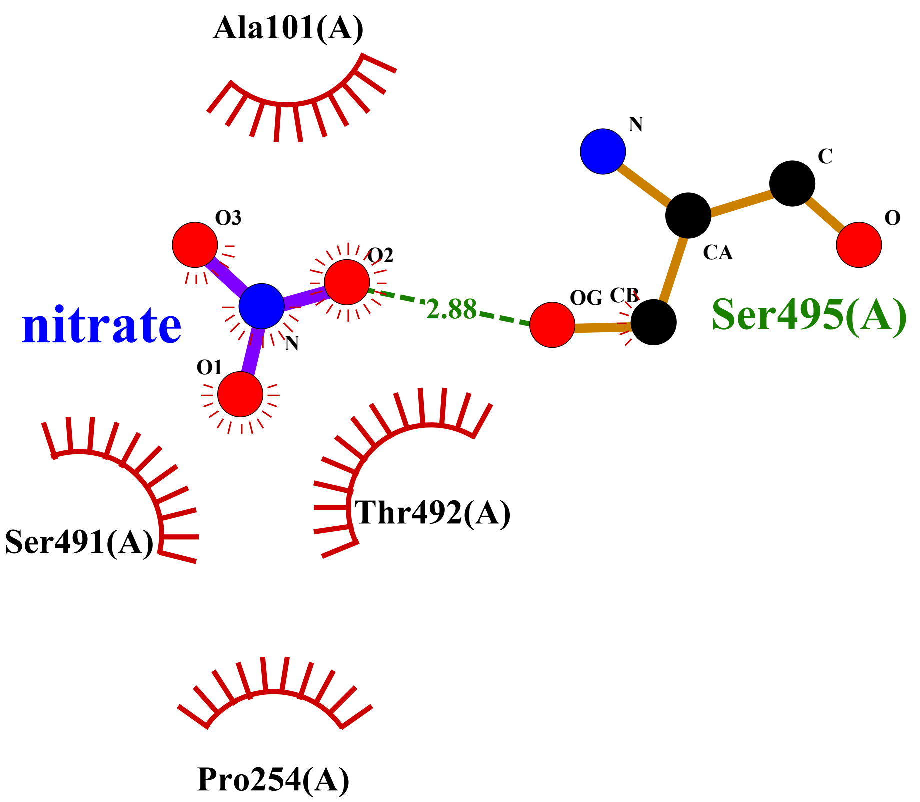
Ala101(A)
N
C
O3
O
O2
CA
OG CB
2.88
nitrate
Ser495(A)
N
O1
Thr492(A)
Ser491(A)
Pro254(A)

Si1g32240.1
