# Supplementary material for: Genome-wide identification and characterization of the NPF genes provide new insight into low nitrogen tolerance in Setaria
Source: Front Plant Sci. 2022 Dec 14;13:1043832. doi: 10.3389/fpls.2022.1043832 (PMC9795848; doi:10.3389/fpls.2022.1043832)
Supplement: Supplementary Figure 1 — Chromosome location and distribution analysis of the SiNPF genes. Tandem duplicated genes are linked by a red curve. [file DataSheet_1.zip › Supplementary Data.zip!/Supplementary Data 3/Si2g07520.1.pdf]

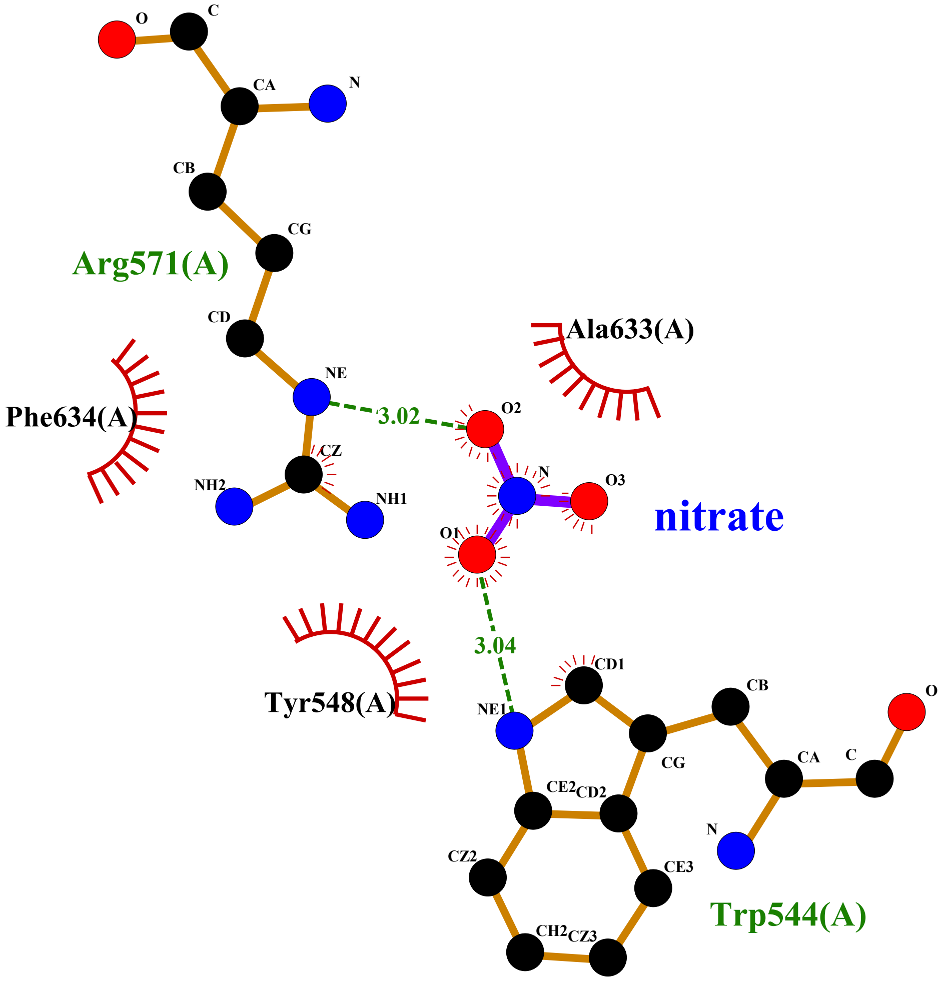

Arg571(A)
O
C
CA
N
CB
CG
CD
NE
CZ
NH2
NH1
Phe634(A)
Ala633(A)
3.02
O2
N
O3
O1
nitrate
Tyr548(A)
3.04
CD1
NE1
CB
O
CG
CA
C
CE2
CD2
N
CZ2
CE3
CH2
CZ3
Trp544(A)

Si2g07520.1
