# Supplementary material for: Genome-wide identification and characterization of the NPF genes provide new insight into low nitrogen tolerance in Setaria
Source: Front Plant Sci. 2022 Dec 14;13:1043832. doi: 10.3389/fpls.2022.1043832 (PMC9795848; doi:10.3389/fpls.2022.1043832)
Supplement: Supplementary Figure 1 — Chromosome location and distribution analysis of the SiNPF genes. Tandem duplicated genes are linked by a red curve. [file DataSheet_1.zip › Supplementary Data.zip!/Supplementary Data 3/Si2g38080.1.pdf]

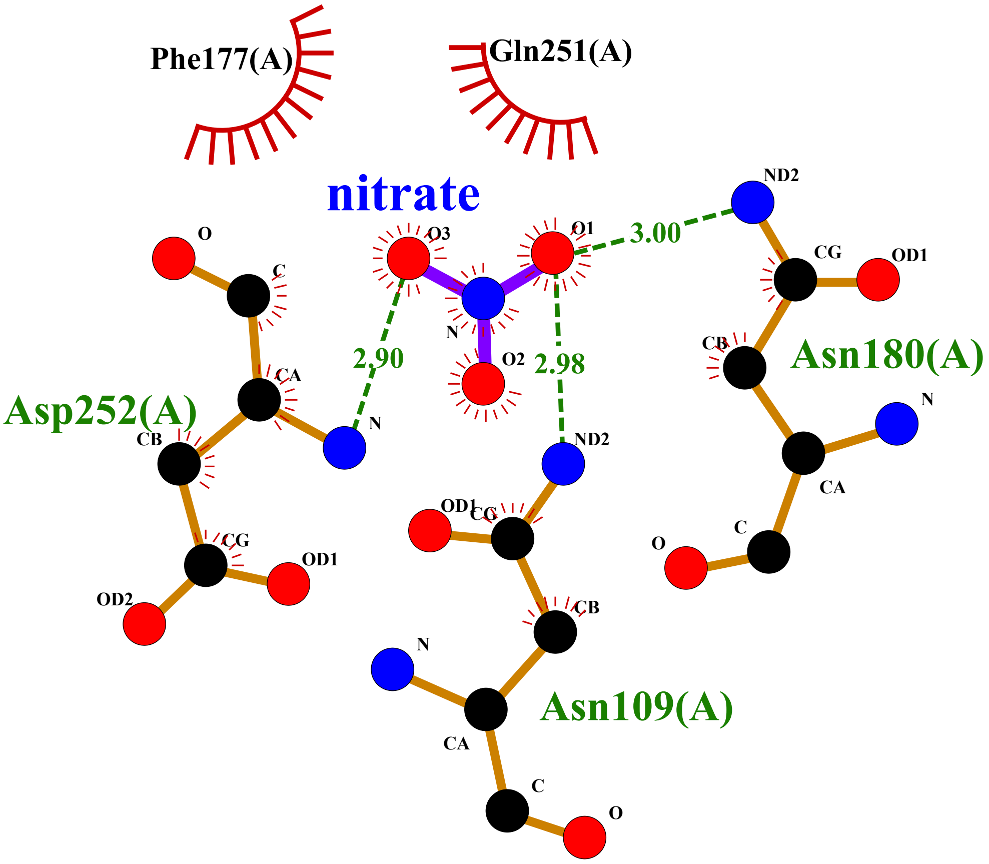

Phe177(A)
Gln251(A)
nitrate
O3
O1
N
O2
3.00
2.90
2.98
ND2
CG
OD1
CB
Asn180(A)
N
CA
C
O
O
C
CA
Asp252(A)
CB
N
CG
OD1
OD2
ND2
OD1
CG
CB
N
Asn109(A)
CA
C
O

Si2g38080.1
