# Supplementary material for: Genome-wide identification and characterization of the NPF genes provide new insight into low nitrogen tolerance in Setaria
Source: Front Plant Sci. 2022 Dec 14;13:1043832. doi: 10.3389/fpls.2022.1043832 (PMC9795848; doi:10.3389/fpls.2022.1043832)
Supplement: Supplementary Figure 1 — Chromosome location and distribution analysis of the SiNPF genes. Tandem duplicated genes are linked by a red curve. [file DataSheet_1.zip › Supplementary Data.zip!/Supplementary Data 3/Si3g01280.1.pdf]

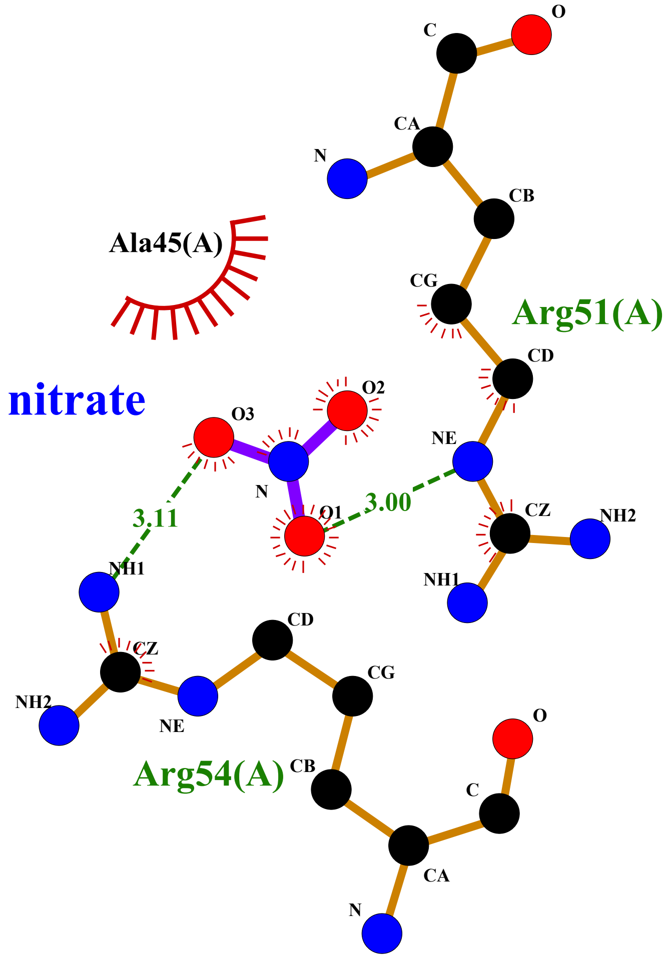
Ala45(A)
Arg51(A)
nitrate
Arg54(A)
3.11
3.00
O3
O2
O1
N
NE
CZ
NH1
NH2
CD
CG
CB
CA
C
O

Si3g01280.1
