# Supplementary material for: Genome-wide identification and characterization of the NPF genes provide new insight into low nitrogen tolerance in Setaria
Source: Front Plant Sci. 2022 Dec 14;13:1043832. doi: 10.3389/fpls.2022.1043832 (PMC9795848; doi:10.3389/fpls.2022.1043832)
Supplement: Supplementary Figure 1 — Chromosome location and distribution analysis of the SiNPF genes. Tandem duplicated genes are linked by a red curve. [file DataSheet_1.zip › Supplementary Data.zip!/Supplementary Data 3/Si3g09770.1.pdf]

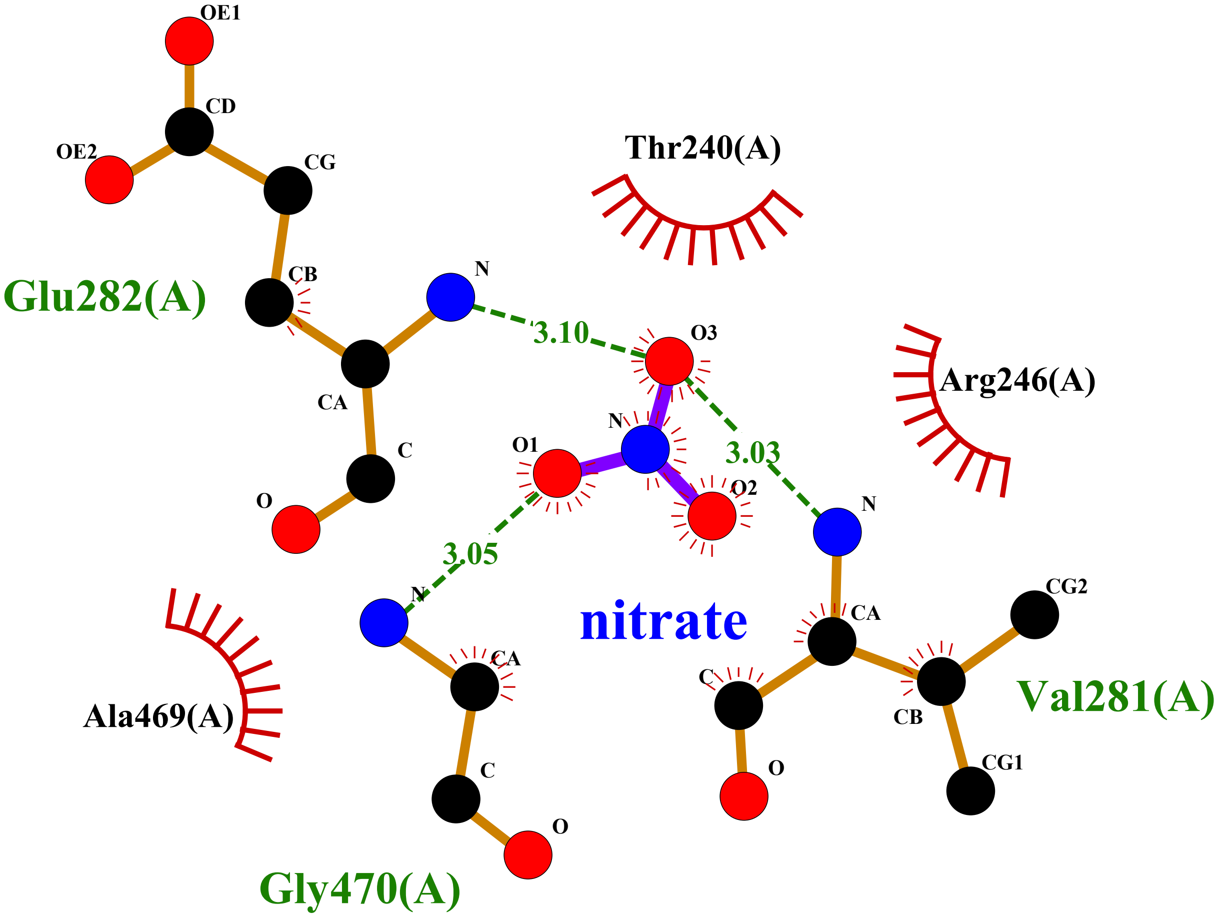
OE1
CD
OE2
CG
Thr240(A)
Glu282(A)
CB
N
3.10
O3
Arg246(A)
CA
N
O1
3.03
C
O2
O
N
3.05
N
nitrate
CA
CG2
Ala469(A)
CA
C
Val281(A)
CB
C
O
CG1
O
Gly470(A)

Si3g09770.1
