# Supplementary material for: Genome-wide identification and characterization of the NPF genes provide new insight into low nitrogen tolerance in Setaria
Source: Front Plant Sci. 2022 Dec 14;13:1043832. doi: 10.3389/fpls.2022.1043832 (PMC9795848; doi:10.3389/fpls.2022.1043832)
Supplement: Supplementary Figure 1 — Chromosome location and distribution analysis of the SiNPF genes. Tandem duplicated genes are linked by a red curve. [file DataSheet_1.zip › Supplementary Data.zip!/Supplementary Data 3/Si3g09780.1.pdf]

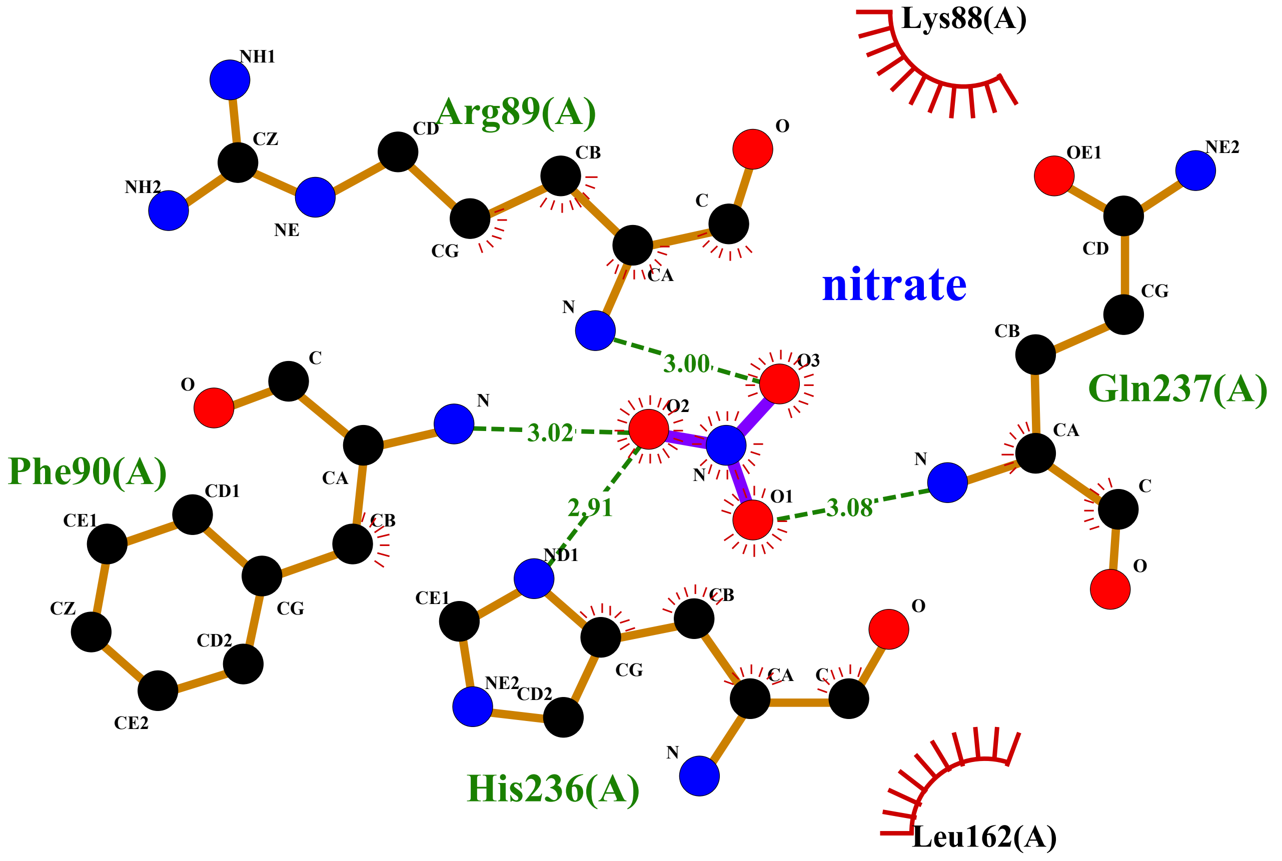
Lys88(A)
Arg89(A)
NH1
CZ
NH2
NE
CD
CG
CB
CA
C
O
N
nitrate
O3
O2
N
O1
3.00
3.02
2.91
3.08
OE1
NE2
CD
CG
CB
Gln237(A)
CA
N
C
O
Phe90(A)
O
C
N
CA
CB
CD1
CE1
CZ
CG
CD2
CE2
ND1
CE1
NE2
CD2
CG
CB
CA
C
O
N
His236(A)
Leu162(A)

Si3g09780.1
