# Supplementary material for: Genome-wide identification and characterization of the NPF genes provide new insight into low nitrogen tolerance in Setaria
Source: Front Plant Sci. 2022 Dec 14;13:1043832. doi: 10.3389/fpls.2022.1043832 (PMC9795848; doi:10.3389/fpls.2022.1043832)
Supplement: Supplementary Figure 1 — Chromosome location and distribution analysis of the SiNPF genes. Tandem duplicated genes are linked by a red curve. [file DataSheet_1.zip › Supplementary Data.zip!/Supplementary Data 3/Si3g23710.1.pdf]

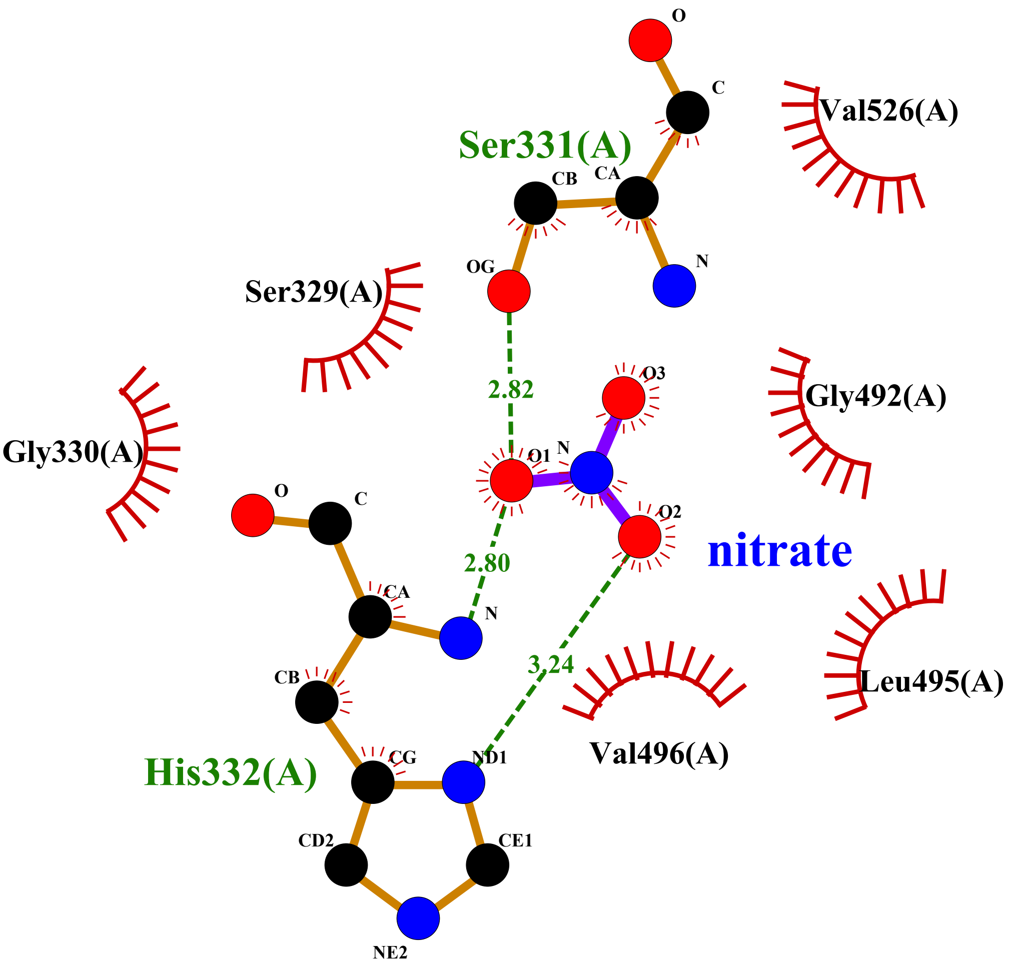

O
C
Val526(A)
Ser331(A)
CB
CA
OG
N
Ser329(A)
2.82
O3
Gly492(A)
Gly330(A)
O1
N
O
C
O2
nitrate
2.80
CA
N
3.24
Leu495(A)
CB
Val496(A)
His332(A)
CG
ND1
CD2
CE1
NE2

Si3g23710.1
