# Supplementary material for: Genome-wide identification and characterization of the NPF genes provide new insight into low nitrogen tolerance in Setaria
Source: Front Plant Sci. 2022 Dec 14;13:1043832. doi: 10.3389/fpls.2022.1043832 (PMC9795848; doi:10.3389/fpls.2022.1043832)
Supplement: Supplementary Figure 1 — Chromosome location and distribution analysis of the SiNPF genes. Tandem duplicated genes are linked by a red curve. [file DataSheet_1.zip › Supplementary Data.zip!/Supplementary Data 3/Si3g23720.1.pdf]

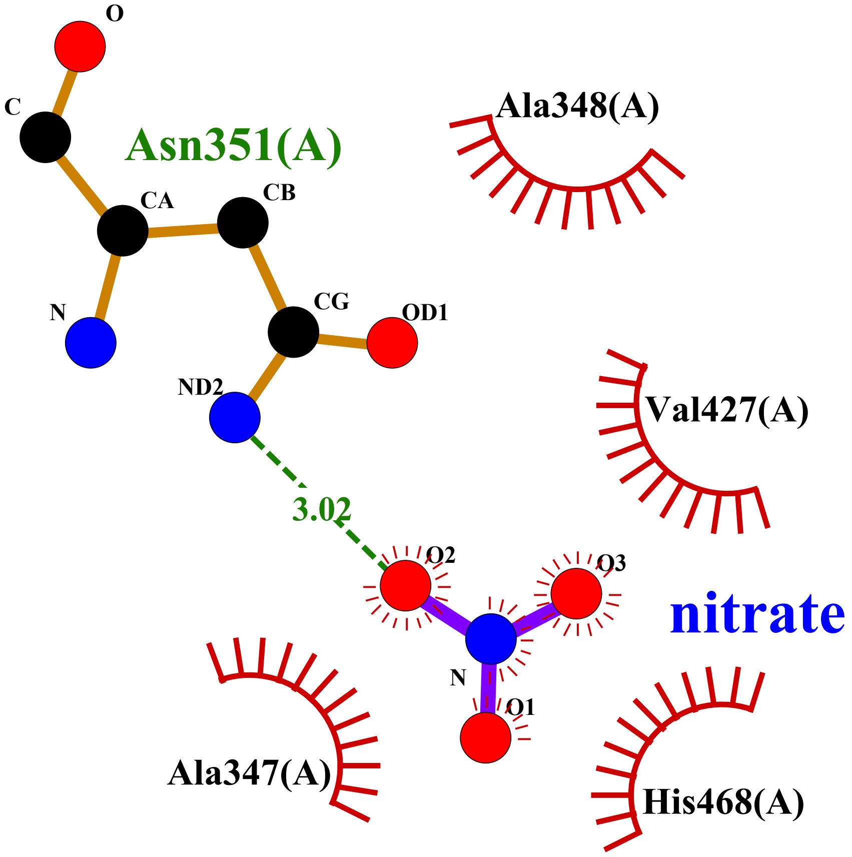
O
C
Asn351(A)
Ala348(A)
CA
CB
CG
OD1
N
ND2
Val427(A)
3.02
O2
O3
nitrate
N
O1
Ala347(A)
His468(A)

Si3g23720.1
