# Supplementary material for: Genome-wide identification and characterization of the NPF genes provide new insight into low nitrogen tolerance in Setaria
Source: Front Plant Sci. 2022 Dec 14;13:1043832. doi: 10.3389/fpls.2022.1043832 (PMC9795848; doi:10.3389/fpls.2022.1043832)
Supplement: Supplementary Figure 1 — Chromosome location and distribution analysis of the SiNPF genes. Tandem duplicated genes are linked by a red curve. [file DataSheet_1.zip › Supplementary Data.zip!/Supplementary Data 3/Si3g23730.1.pdf]

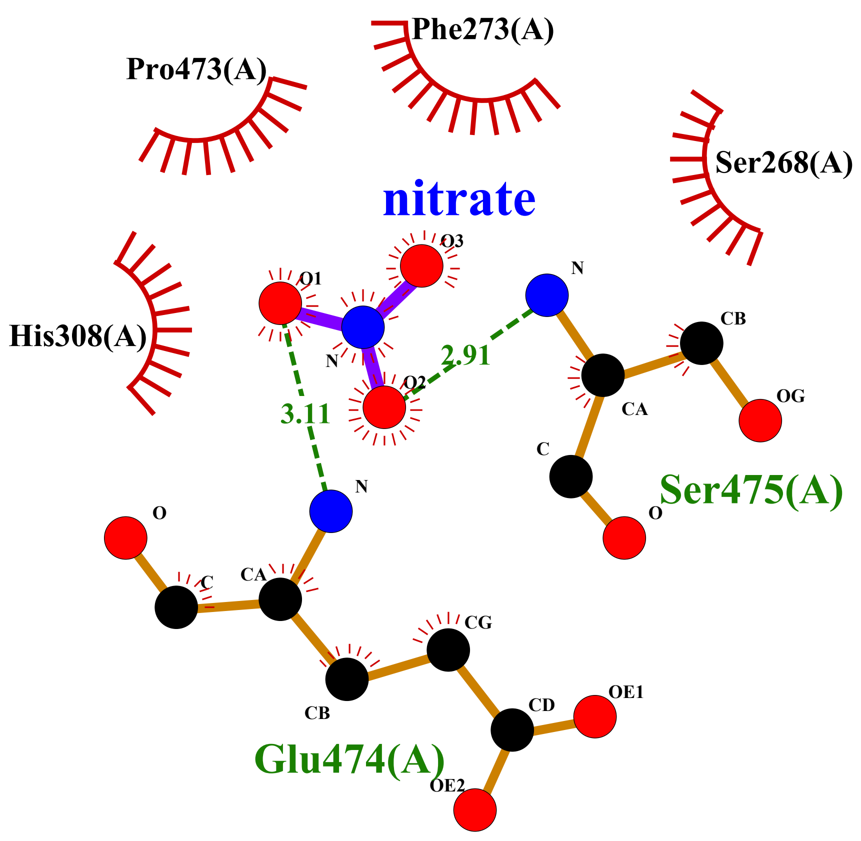
Phe273(A)
Pro473(A)
Ser268(A)
nitrate
O3
O1
N
His308(A)
N
O2
2.91
CB
CA
OG
3.11
C
Ser475(A)
N
O
O
C
CA
CG
CB
CD
OE1
Glu474(A)
OE2

Si3g23730.1
