# Supplementary material for: Genome-wide identification and characterization of the NPF genes provide new insight into low nitrogen tolerance in Setaria
Source: Front Plant Sci. 2022 Dec 14;13:1043832. doi: 10.3389/fpls.2022.1043832 (PMC9795848; doi:10.3389/fpls.2022.1043832)
Supplement: Supplementary Figure 1 — Chromosome location and distribution analysis of the SiNPF genes. Tandem duplicated genes are linked by a red curve. [file DataSheet_1.zip › Supplementary Data.zip!/Supplementary Data 3/Si3g23740.1.pdf]

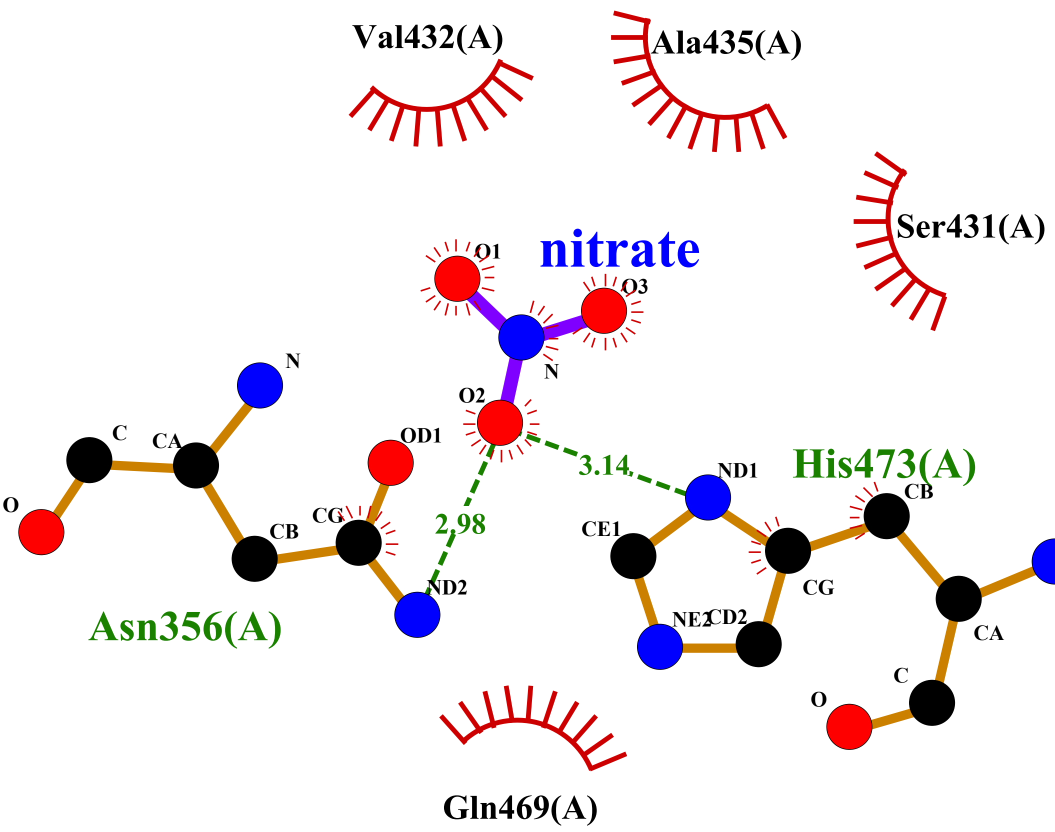
Val432(A)
Ala435(A)
Ser431(A)
nitrate
O1
O3
N
O2
N
C
CA
OD1
O
CB
CG
2.98
ND2
3.14
ND1
His473(A)
CB
CE1
CG
N
NE2
CD2
CA
Asn356(A)
C
O
Gln469(A)

Si3g23740.1
