# Supplementary material for: Genome-wide identification and characterization of the NPF genes provide new insight into low nitrogen tolerance in Setaria
Source: Front Plant Sci. 2022 Dec 14;13:1043832. doi: 10.3389/fpls.2022.1043832 (PMC9795848; doi:10.3389/fpls.2022.1043832)
Supplement: Supplementary Figure 1 — Chromosome location and distribution analysis of the SiNPF genes. Tandem duplicated genes are linked by a red curve. [file DataSheet_1.zip › Supplementary Data.zip!/Supplementary Data 3/Si3g23750.1.pdf]

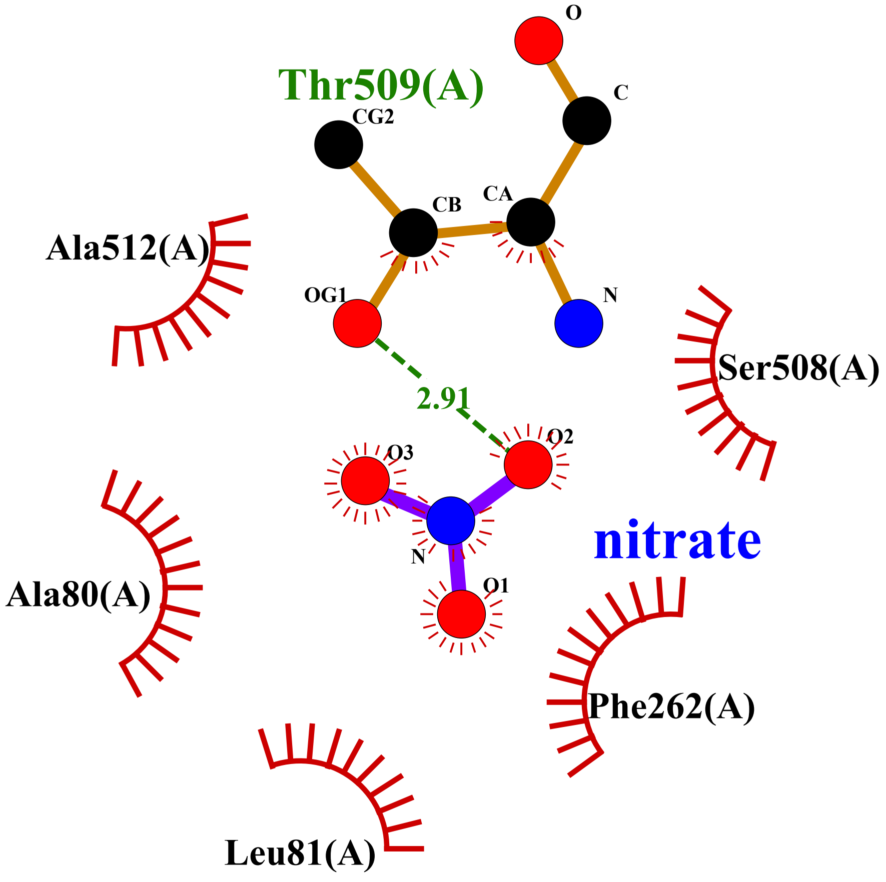

Thr509(A)
O
C
CG2
CB
CA
Ala512(A)
OG1
N
Ser508(A)
2.91
O3
O2
N
nitrate
Ala80(A)
O1
Phe262(A)
Leu81(A)

Si3g23750.1
