# Supplementary material for: Genome-wide identification and characterization of the NPF genes provide new insight into low nitrogen tolerance in Setaria
Source: Front Plant Sci. 2022 Dec 14;13:1043832. doi: 10.3389/fpls.2022.1043832 (PMC9795848; doi:10.3389/fpls.2022.1043832)
Supplement: Supplementary Figure 1 — Chromosome location and distribution analysis of the SiNPF genes. Tandem duplicated genes are linked by a red curve. [file DataSheet_1.zip › Supplementary Data.zip!/Supplementary Data 3/Si3g27640.1.pdf]

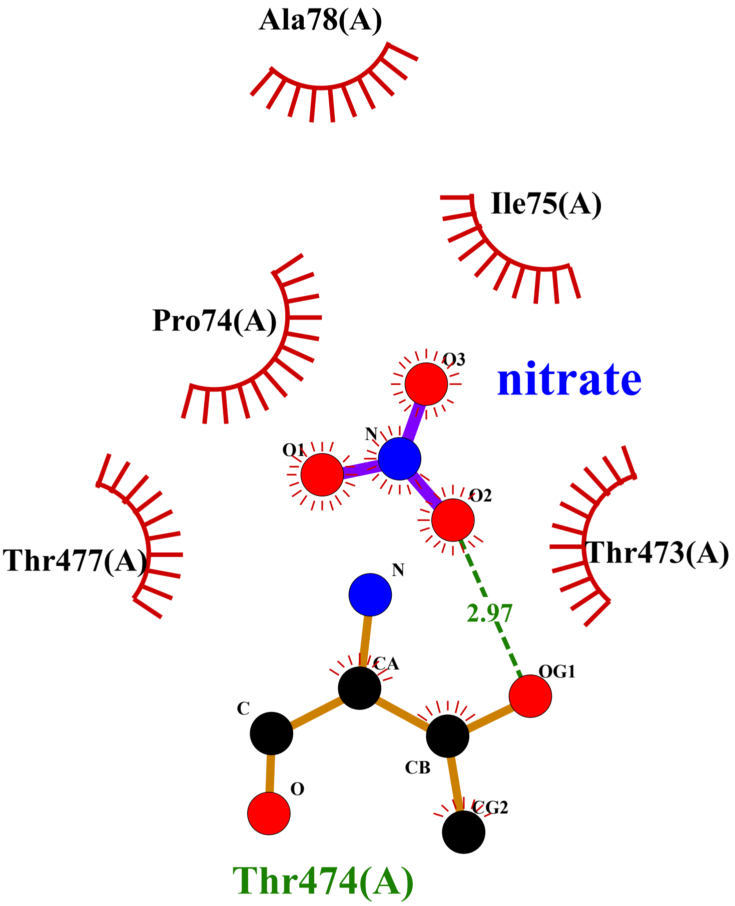

Ala78(A)
Ile75(A)
Pro74(A)
O3
nitrate
O1
N
O2
Thr477(A)
Thr473(A)
N
2.97
CA
OG1
C
CB
O
CG2
Thr474(A)

Si3g27640.1
