# Supplementary material for: Genome-wide identification and characterization of the NPF genes provide new insight into low nitrogen tolerance in Setaria
Source: Front Plant Sci. 2022 Dec 14;13:1043832. doi: 10.3389/fpls.2022.1043832 (PMC9795848; doi:10.3389/fpls.2022.1043832)
Supplement: Supplementary Figure 1 — Chromosome location and distribution analysis of the SiNPF genes. Tandem duplicated genes are linked by a red curve. [file DataSheet_1.zip › Supplementary Data.zip!/Supplementary Data 3/Si4g09490.1.pdf]

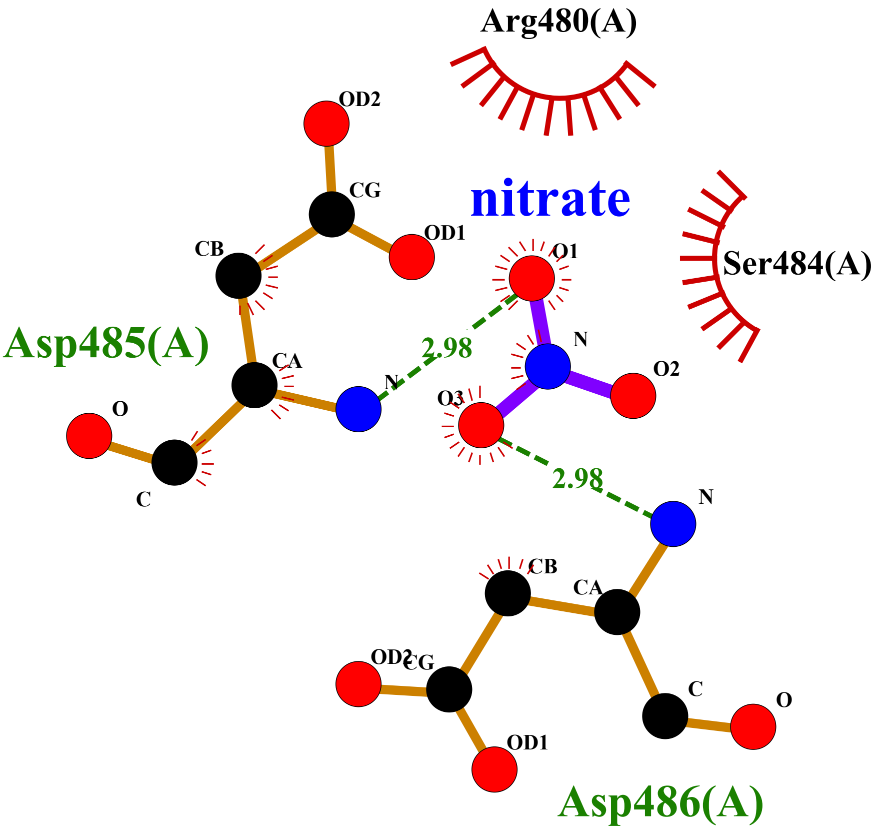
Arg480(A)
nitrate
Ser484(A)
Asp485(A)
Asp486(A)
OD2
CG
OD1
CB
CA
N
O
C
O1
N
O2
O3
2.98
2.98
N
CB
CA
OD2
CG
OD1
C
O

# Si4g09490.1
