# Supplementary material for: Genome-wide identification and characterization of the NPF genes provide new insight into low nitrogen tolerance in Setaria
Source: Front Plant Sci. 2022 Dec 14;13:1043832. doi: 10.3389/fpls.2022.1043832 (PMC9795848; doi:10.3389/fpls.2022.1043832)
Supplement: Supplementary Figure 1 — Chromosome location and distribution analysis of the SiNPF genes. Tandem duplicated genes are linked by a red curve. [file DataSheet_1.zip › Supplementary Data.zip!/Supplementary Data 3/Si4g10420.1.pdf]

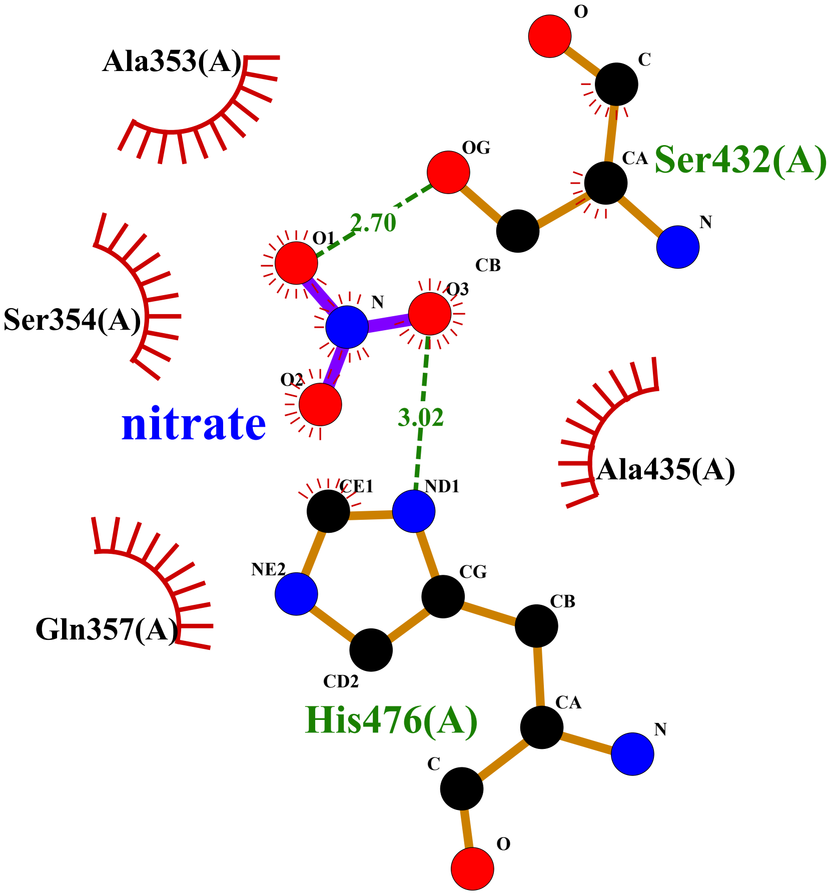
Ala353(A)
Ser354(A)
nitrate
Gln357(A)
O
C
OG
CA
Ser432(A)
N
2.70
O1
CB
N
O3
O2
3.02
Ala435(A)
CE1
ND1
NE2
CG
CB
CD2
CA
His476(A)
N
C
O

Si4g10420.1
