# Supplementary material for: Genome-wide identification and characterization of the NPF genes provide new insight into low nitrogen tolerance in Setaria
Source: Front Plant Sci. 2022 Dec 14;13:1043832. doi: 10.3389/fpls.2022.1043832 (PMC9795848; doi:10.3389/fpls.2022.1043832)
Supplement: Supplementary Figure 1 — Chromosome location and distribution analysis of the SiNPF genes. Tandem duplicated genes are linked by a red curve. [file DataSheet_1.zip › Supplementary Data.zip!/Supplementary Data 3/Si4g22250.1.pdf]

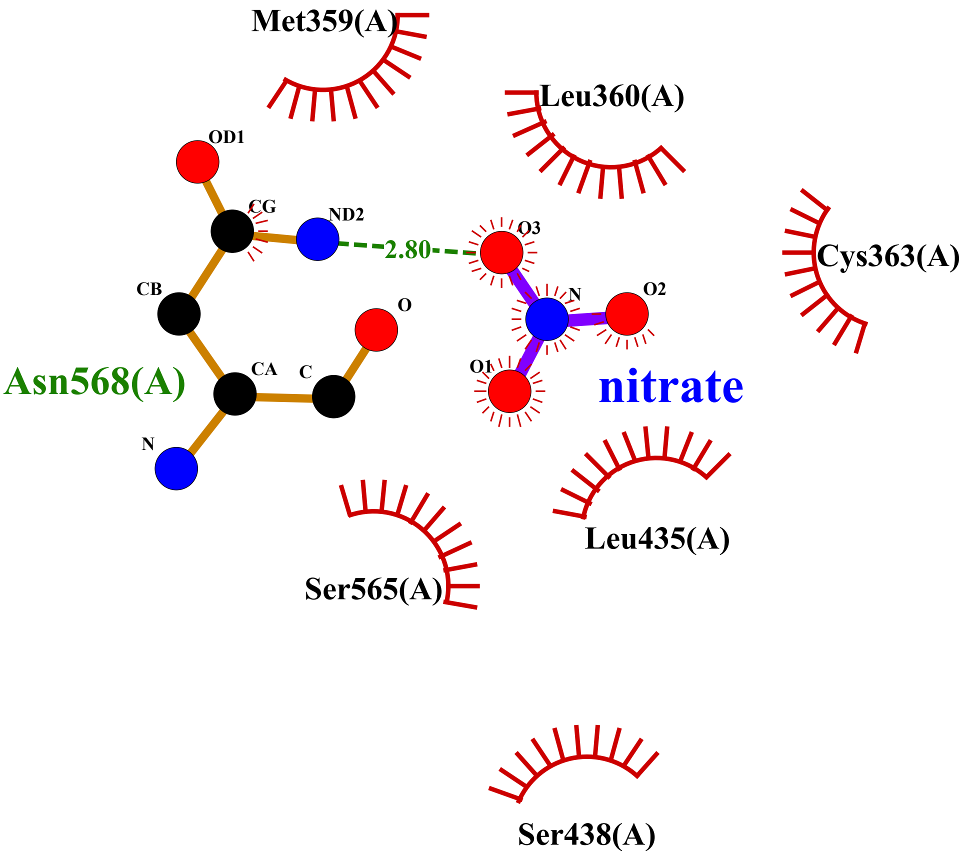
Met359(A)
Leu360(A)
OD1
CG
ND2
2.80
O3
Cys363(A)
CB
N
O2
O
CA
C
O1
Asn568(A)
nitrate
N
Leu435(A)
Ser565(A)
Ser438(A)

Si4g22250.1
