# Supplementary material for: Genome-wide identification and characterization of the NPF genes provide new insight into low nitrogen tolerance in Setaria
Source: Front Plant Sci. 2022 Dec 14;13:1043832. doi: 10.3389/fpls.2022.1043832 (PMC9795848; doi:10.3389/fpls.2022.1043832)
Supplement: Supplementary Figure 1 — Chromosome location and distribution analysis of the SiNPF genes. Tandem duplicated genes are linked by a red curve. [file DataSheet_1.zip › Supplementary Data.zip!/Supplementary Data 3/Si5g07510.1.pdf]

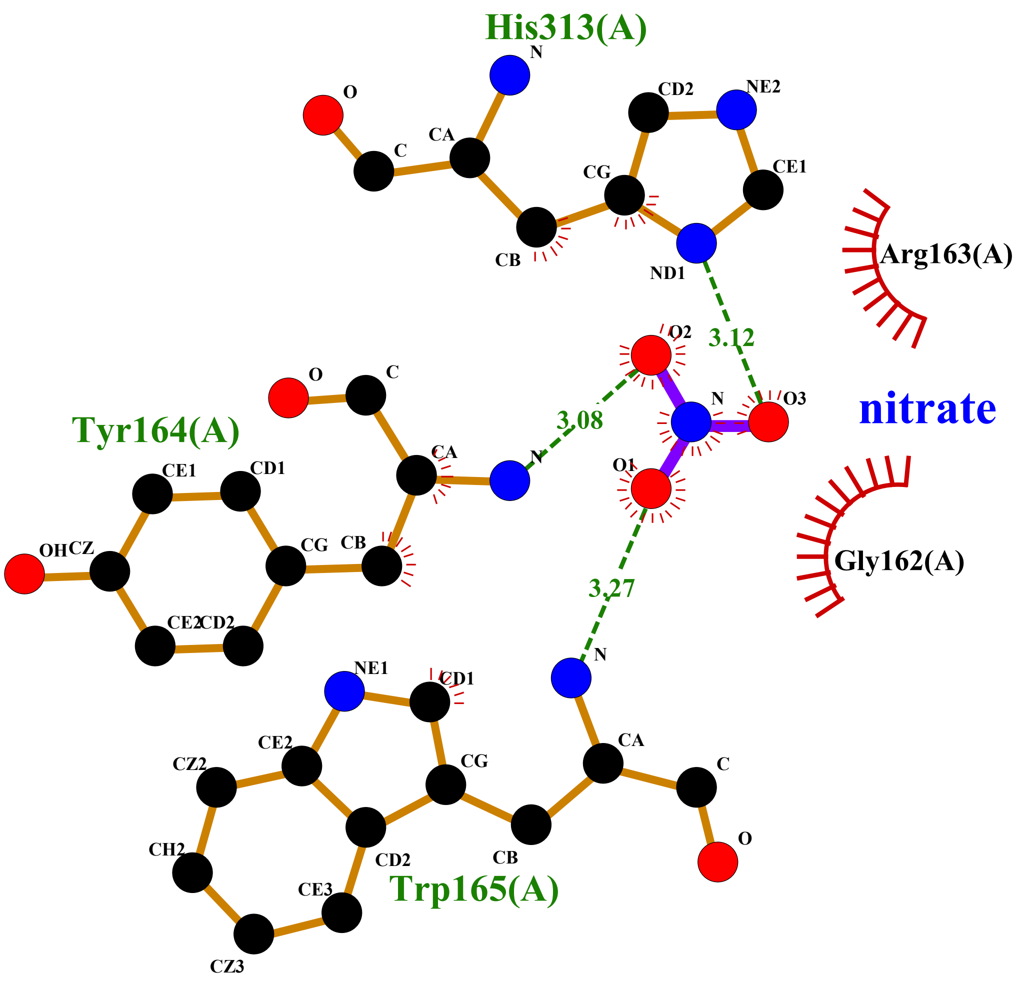
His313(A)
N
O
C
CA
CD2
NE2
CE1
CG
CB
ND1
Arg163(A)
3.12
O2
O
C
N
O3
nitrate
Tyr164(A)
3.08
CE1
CD1
CA
N
O1
OH
CZ
CG
CB
Gly162(A)
3.27
CE2
CD2
N
NE1
CD1
CE2
CA
CZ2
CG
C
O
CH2
CD2
CB
CE3
Trp165(A)
CZ3

Si5g07510.1
