# Supplementary material for: Genome-wide identification and characterization of the NPF genes provide new insight into low nitrogen tolerance in Setaria
Source: Front Plant Sci. 2022 Dec 14;13:1043832. doi: 10.3389/fpls.2022.1043832 (PMC9795848; doi:10.3389/fpls.2022.1043832)
Supplement: Supplementary Figure 1 — Chromosome location and distribution analysis of the SiNPF genes. Tandem duplicated genes are linked by a red curve. [file DataSheet_1.zip › Supplementary Data.zip!/Supplementary Data 3/Si5g10290.1.pdf]

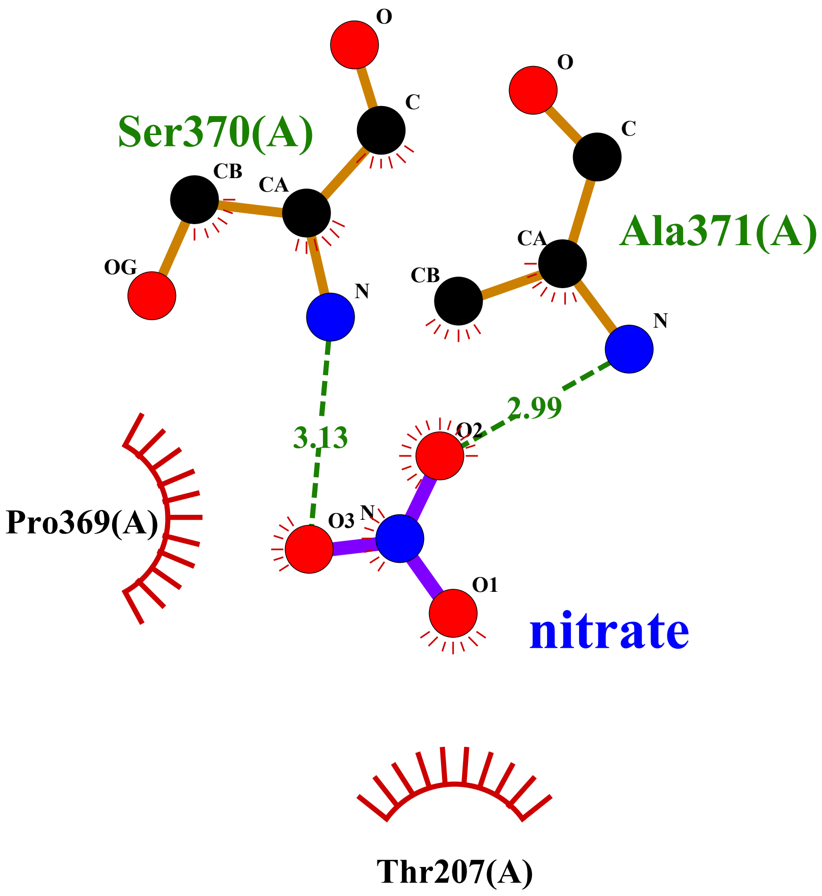
Ser370(A)
O
C
CB
CA
OG
N
O
C
Ala371(A)
CA
CB
N
2.99
3.13
O2
Pro369(A)
O3
N
O1
nitrate
Thr207(A)

Si5g10290.1
