# Supplementary material for: Genome-wide identification and characterization of the NPF genes provide new insight into low nitrogen tolerance in Setaria
Source: Front Plant Sci. 2022 Dec 14;13:1043832. doi: 10.3389/fpls.2022.1043832 (PMC9795848; doi:10.3389/fpls.2022.1043832)
Supplement: Supplementary Figure 1 — Chromosome location and distribution analysis of the SiNPF genes. Tandem duplicated genes are linked by a red curve. [file DataSheet_1.zip › Supplementary Data.zip!/Supplementary Data 3/Si5g10300.1.pdf]

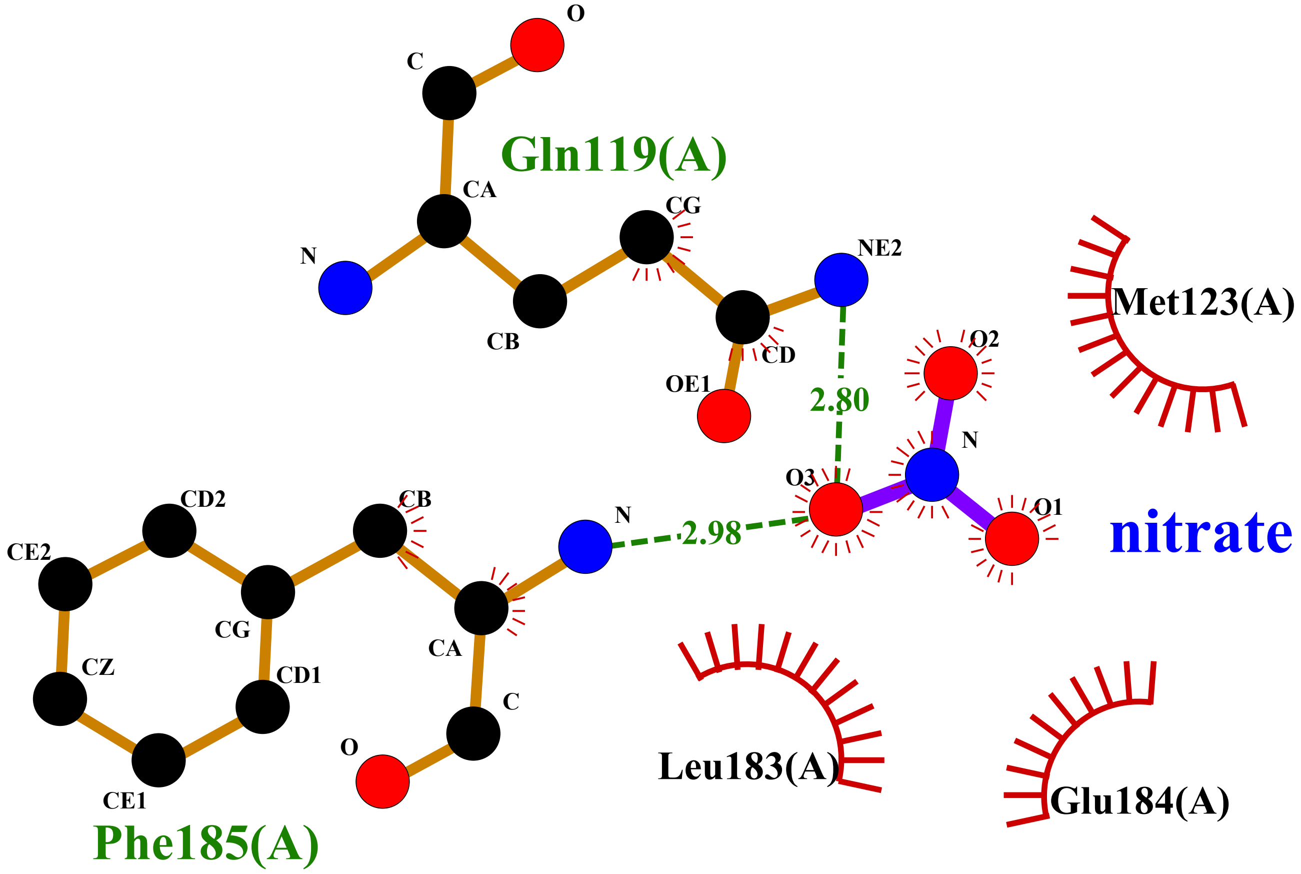
O
C
Gln119(A)
CA
CG
N
NE2
CB
CD
OE1
2.80
O2
Met123(A)
N
O3
CB
CD2
N
2.98
O1
nitrate
CE2
CG
CA
CZ
CD1
C
O
CE1
Phe185(A)
Leu183(A)
Glu184(A)

Si5g10300.1
