# Supplementary material for: Genome-wide identification and characterization of the NPF genes provide new insight into low nitrogen tolerance in Setaria
Source: Front Plant Sci. 2022 Dec 14;13:1043832. doi: 10.3389/fpls.2022.1043832 (PMC9795848; doi:10.3389/fpls.2022.1043832)
Supplement: Supplementary Figure 1 — Chromosome location and distribution analysis of the SiNPF genes. Tandem duplicated genes are linked by a red curve. [file DataSheet_1.zip › Supplementary Data.zip!/Supplementary Data 3/Si5g20360.1.pdf]

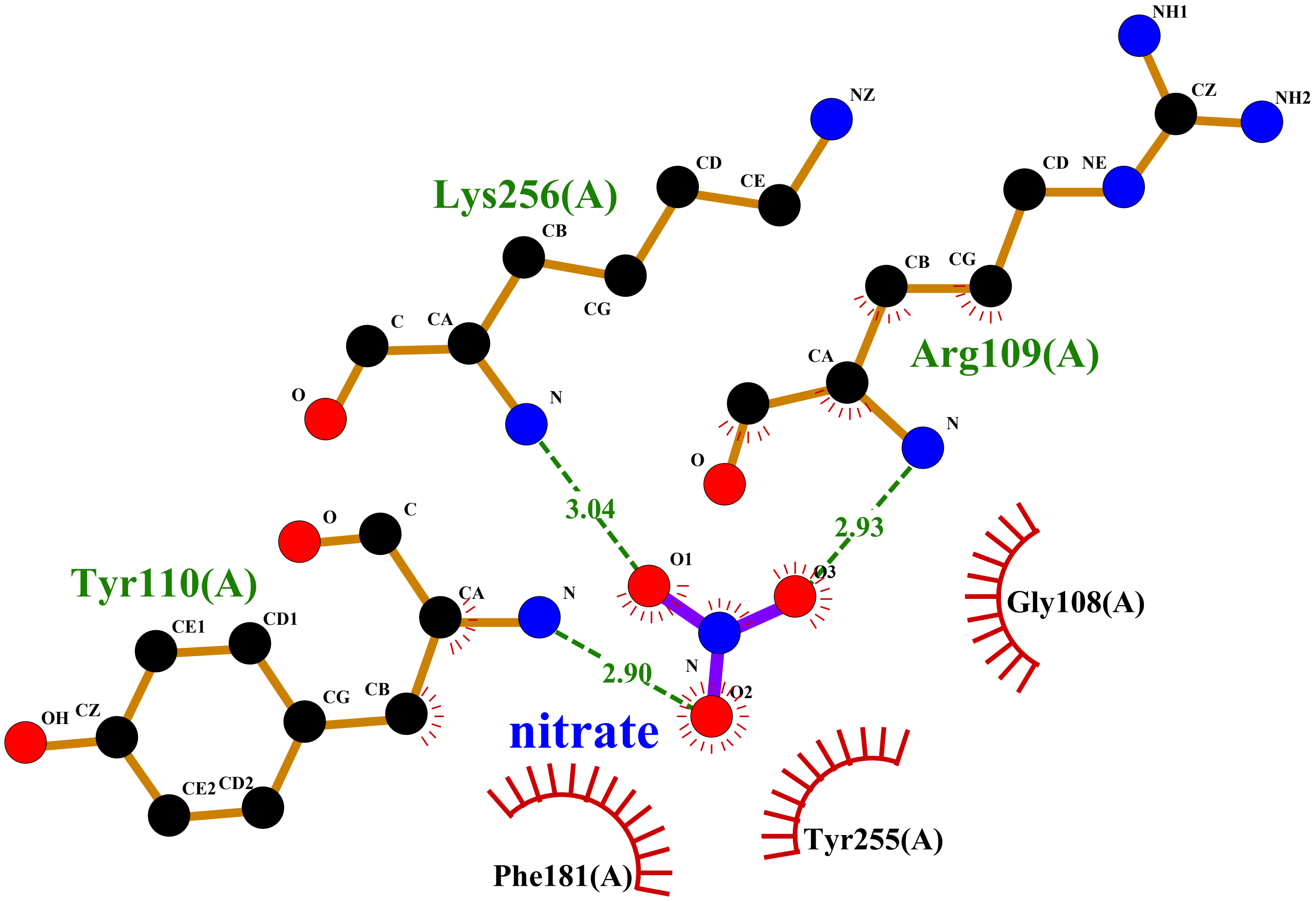
NH1
CZ
NH2
NZ
CD
CE
CD NE
Lys256(A)
CB
CB CG
CG
C CA
CA
Arg109(A)
O
N
N
O
3.04
2.93
O C
O1
O3
Tyr110(A)
CA
N
Gly108(A)
CE1 CD1
2.90
N
O2
OH CZ
CG CB
nitrate
CE2 CD2
Tyr255(A)
Phe181(A)

Si5g20360.1
