# Supplementary material for: Genome-wide identification and characterization of the NPF genes provide new insight into low nitrogen tolerance in Setaria
Source: Front Plant Sci. 2022 Dec 14;13:1043832. doi: 10.3389/fpls.2022.1043832 (PMC9795848; doi:10.3389/fpls.2022.1043832)
Supplement: Supplementary Figure 1 — Chromosome location and distribution analysis of the SiNPF genes. Tandem duplicated genes are linked by a red curve. [file DataSheet_1.zip › Supplementary Data.zip!/Supplementary Data 3/Si5g28840.1.pdf]

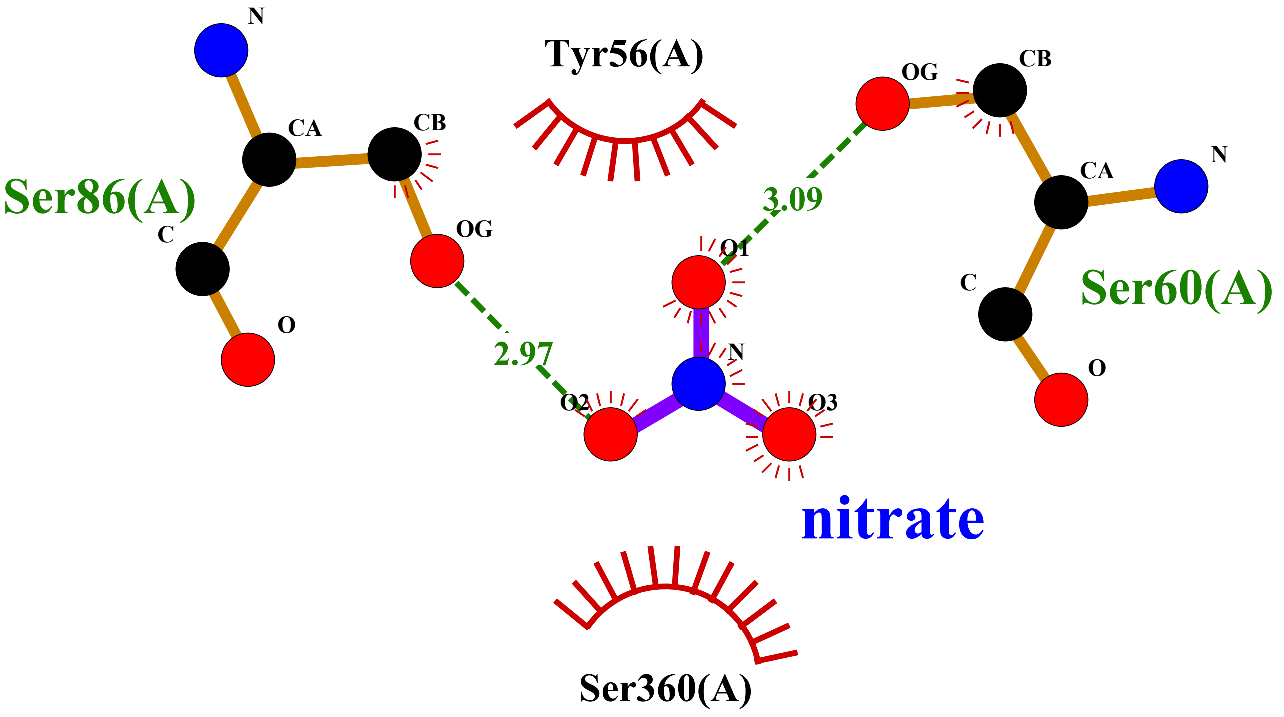
Tyr56(A)
Ser86(A)
N
CA
CB
C
OG
O
2.97
3.09
OG
CB
CA
N
C
O
Ser60(A)
O1
N
O2
O3
nitrate
Ser360(A)

# Si5g28840.1
