# Supplementary material for: Genome-wide identification and characterization of the NPF genes provide new insight into low nitrogen tolerance in Setaria
Source: Front Plant Sci. 2022 Dec 14;13:1043832. doi: 10.3389/fpls.2022.1043832 (PMC9795848; doi:10.3389/fpls.2022.1043832)
Supplement: Supplementary Figure 1 — Chromosome location and distribution analysis of the SiNPF genes. Tandem duplicated genes are linked by a red curve. [file DataSheet_1.zip › Supplementary Data.zip!/Supplementary Data 3/Si5g33140.1.pdf]

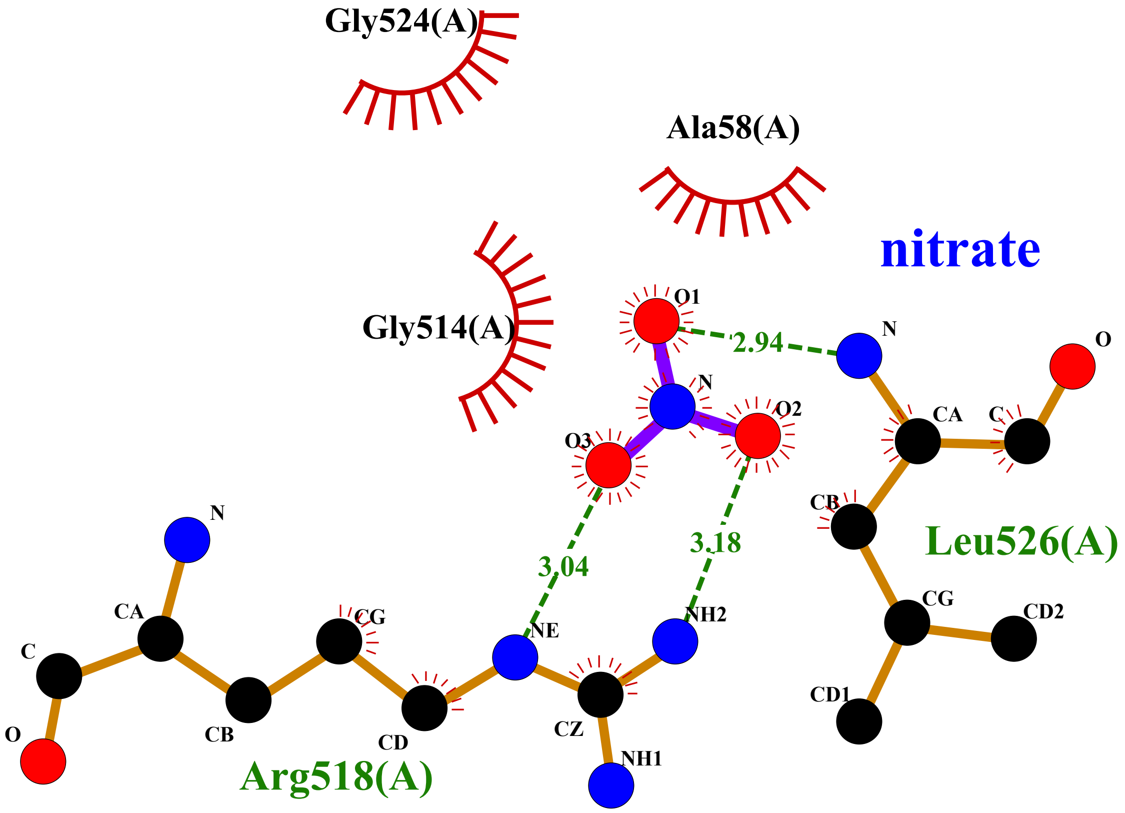
Gly524(A)
Ala58(A)
nitrate
Gly514(A)
O1
2.94
N
O
N
O2
O3
CA
C
CB
N
Leu526(A)
3.04
3.18
CA
CG
NH2
CG
CD2
C
NE
CD1
O
CB
CD
CZ
NH1
Arg518(A)

Si5g33140.1
