# Supplementary material for: Genome-wide identification and characterization of the NPF genes provide new insight into low nitrogen tolerance in Setaria
Source: Front Plant Sci. 2022 Dec 14;13:1043832. doi: 10.3389/fpls.2022.1043832 (PMC9795848; doi:10.3389/fpls.2022.1043832)
Supplement: Supplementary Figure 1 — Chromosome location and distribution analysis of the SiNPF genes. Tandem duplicated genes are linked by a red curve. [file DataSheet_1.zip › Supplementary Data.zip!/Supplementary Data 3/Si5g39680.1.pdf]

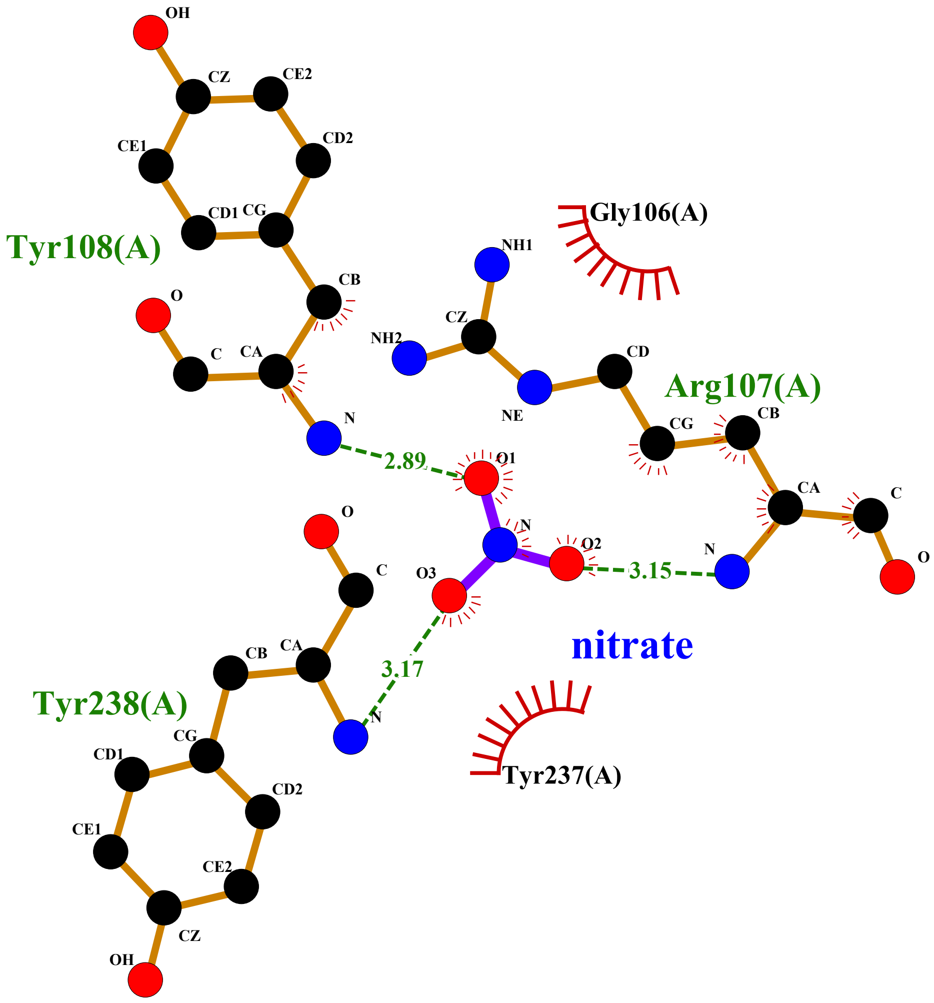
OH
CZ
CE2
CE1
CD2
CD1
CG
Gly106(A)
Tyr108(A)
NH1
CB
O
CZ
NH2
C
CA
CD
Arg107(A)
NE
N
CG
CB
2.89
O1
CA
C
O
N
C
O2
O3
3.15
N
O
nitrate
CB
CA
3.17
Tyr238(A)
N
CG
CD1
Tyr237(A)
CD2
CE1
CE2
CZ
OH

# Si5g39680.1
