# Supplementary material for: Genome-wide identification and characterization of the NPF genes provide new insight into low nitrogen tolerance in Setaria
Source: Front Plant Sci. 2022 Dec 14;13:1043832. doi: 10.3389/fpls.2022.1043832 (PMC9795848; doi:10.3389/fpls.2022.1043832)
Supplement: Supplementary Figure 1 — Chromosome location and distribution analysis of the SiNPF genes. Tandem duplicated genes are linked by a red curve. [file DataSheet_1.zip › Supplementary Data.zip!/Supplementary Data 3/Si5g39690.1.pdf]

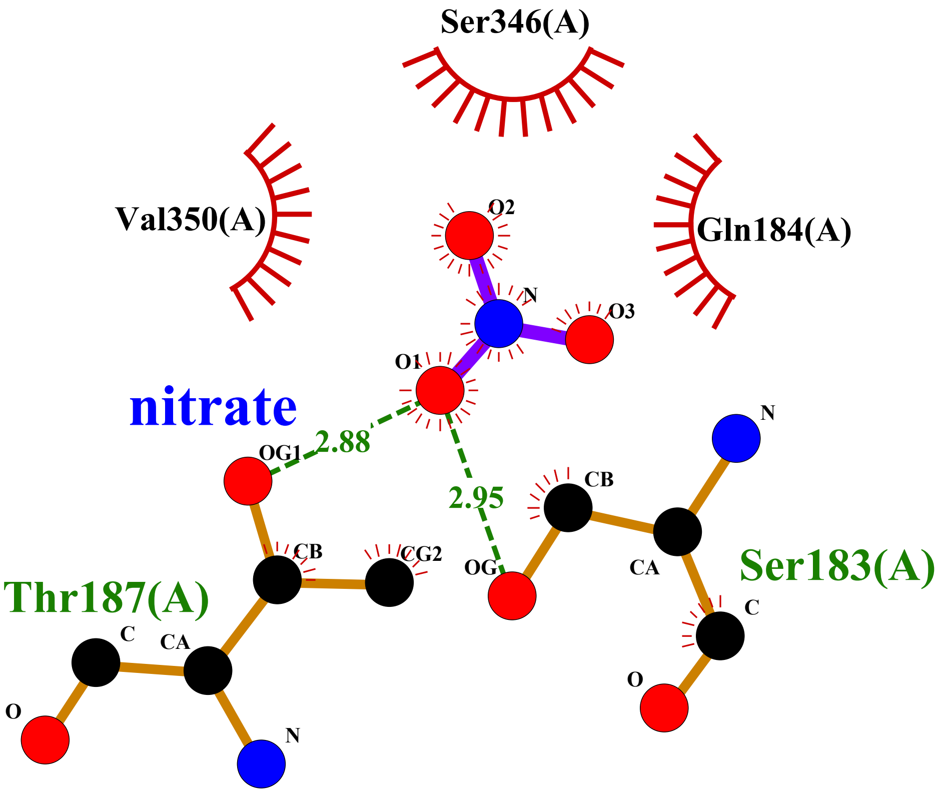
Ser346(A)
Val350(A)
Gln184(A)
O2
N
O3
O1
nitrate
OG1
2.88
2.95
CB
N
CB
CG2
OG
CA
Ser183(A)
Thr187(A)
C
C
CA
O
O
N

# Si5g39690.1
