# Supplementary material for: Genome-wide identification and characterization of the NPF genes provide new insight into low nitrogen tolerance in Setaria
Source: Front Plant Sci. 2022 Dec 14;13:1043832. doi: 10.3389/fpls.2022.1043832 (PMC9795848; doi:10.3389/fpls.2022.1043832)
Supplement: Supplementary Figure 1 — Chromosome location and distribution analysis of the SiNPF genes. Tandem duplicated genes are linked by a red curve. [file DataSheet_1.zip › Supplementary Data.zip!/Supplementary Data 3/Si5g39700.1.pdf]

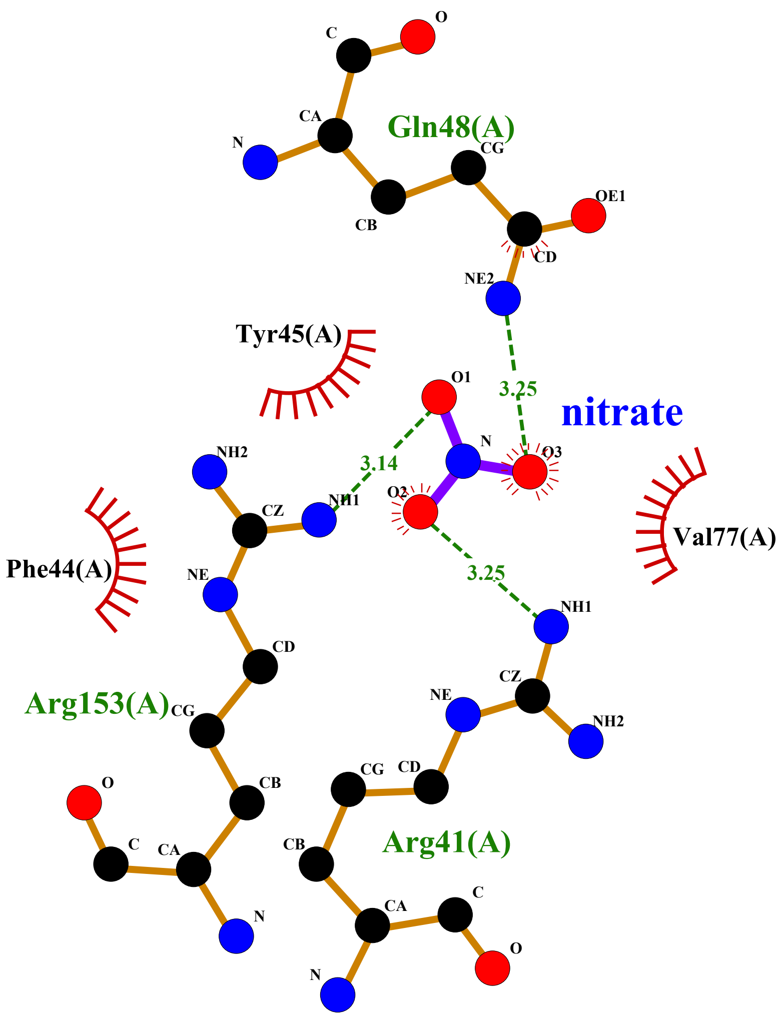

Gln48(A)
C
O
CA
N
CB
CG
OE1
CD
NE2
Tyr45(A)
O1
3.25
nitrate
NH2
3.14
N
O3
CZ
NH1
O2
Val77(A)
Phe44(A)
NE
3.25
NH1
CD
CZ
Arg153(A)
CG
NE
NH2
O
CB
CG
CD
C
CA
CB
Arg41(A)
N
CA
C
O
N

Si5g39700.1
