# Supplementary material for: Genome-wide identification and characterization of the NPF genes provide new insight into low nitrogen tolerance in Setaria
Source: Front Plant Sci. 2022 Dec 14;13:1043832. doi: 10.3389/fpls.2022.1043832 (PMC9795848; doi:10.3389/fpls.2022.1043832)
Supplement: Supplementary Figure 1 — Chromosome location and distribution analysis of the SiNPF genes. Tandem duplicated genes are linked by a red curve. [file DataSheet_1.zip › Supplementary Data.zip!/Supplementary Data 3/Si5g39710.2.pdf]

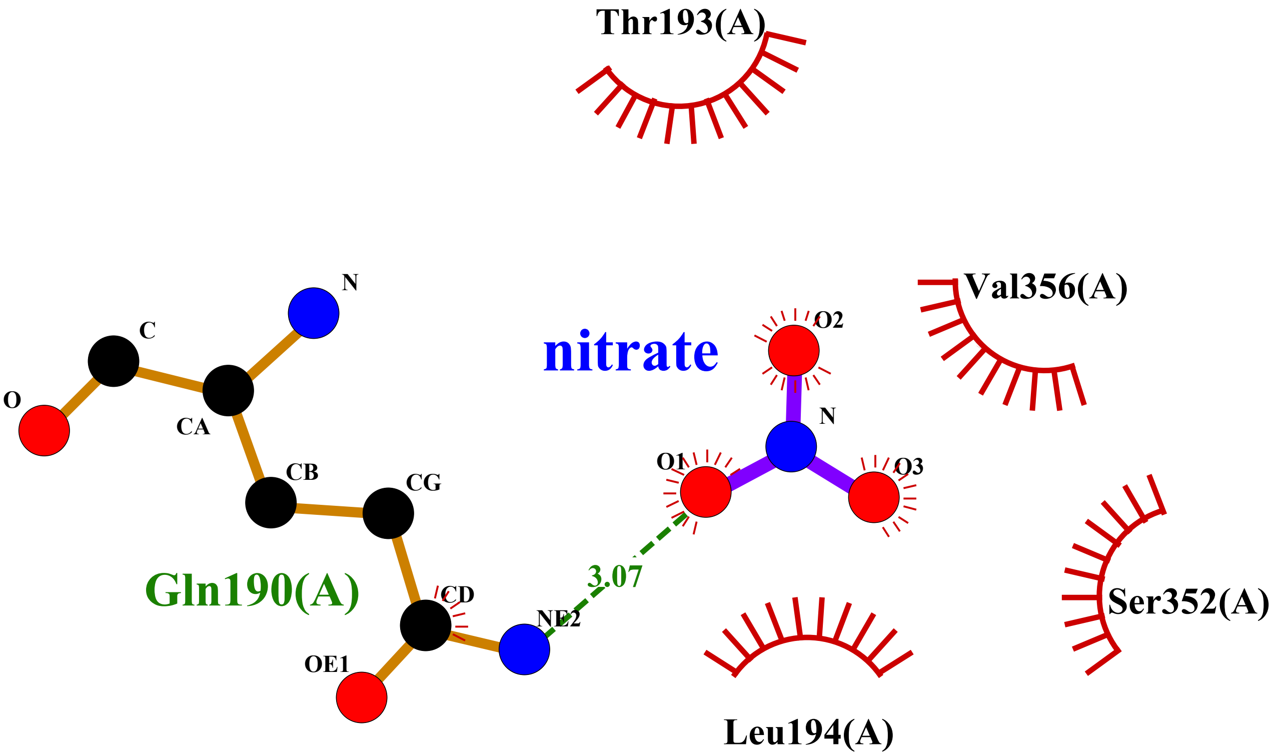
Thr193(A)
Val356(A)
nitrate
O2
N
O1
O3
3.07
C
O
CA
CB
CG
CD
NE2
OE1
Gln190(A)
Ser352(A)
Leu194(A)

Si5g39710.2
