# Supplementary material for: Genome-wide identification and characterization of the NPF genes provide new insight into low nitrogen tolerance in Setaria
Source: Front Plant Sci. 2022 Dec 14;13:1043832. doi: 10.3389/fpls.2022.1043832 (PMC9795848; doi:10.3389/fpls.2022.1043832)
Supplement: Supplementary Figure 1 — Chromosome location and distribution analysis of the SiNPF genes. Tandem duplicated genes are linked by a red curve. [file DataSheet_1.zip › Supplementary Data.zip!/Supplementary Data 3/Si5g39720.1.pdf]

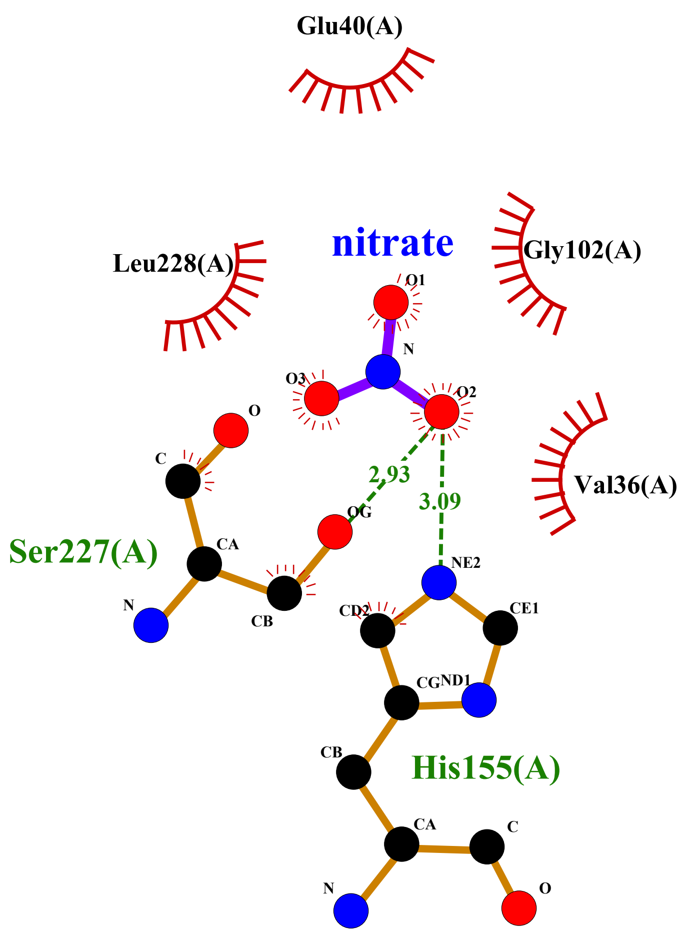
Glu40(A)
nitrate
Leu228(A)
Gly102(A)
O1
N
O3
O2
O
C
2.93
3.09
Val36(A)
OG
Ser227(A)
CA
NE2
N
CB
CD2
CE1
CG
ND1
CB
His155(A)
CA
C
N
O

Si5g39720.1
