# Supplementary material for: Genome-wide identification and characterization of the NPF genes provide new insight into low nitrogen tolerance in Setaria
Source: Front Plant Sci. 2022 Dec 14;13:1043832. doi: 10.3389/fpls.2022.1043832 (PMC9795848; doi:10.3389/fpls.2022.1043832)
Supplement: Supplementary Figure 1 — Chromosome location and distribution analysis of the SiNPF genes. Tandem duplicated genes are linked by a red curve. [file DataSheet_1.zip › Supplementary Data.zip!/Supplementary Data 3/Si5g39730.1.pdf]

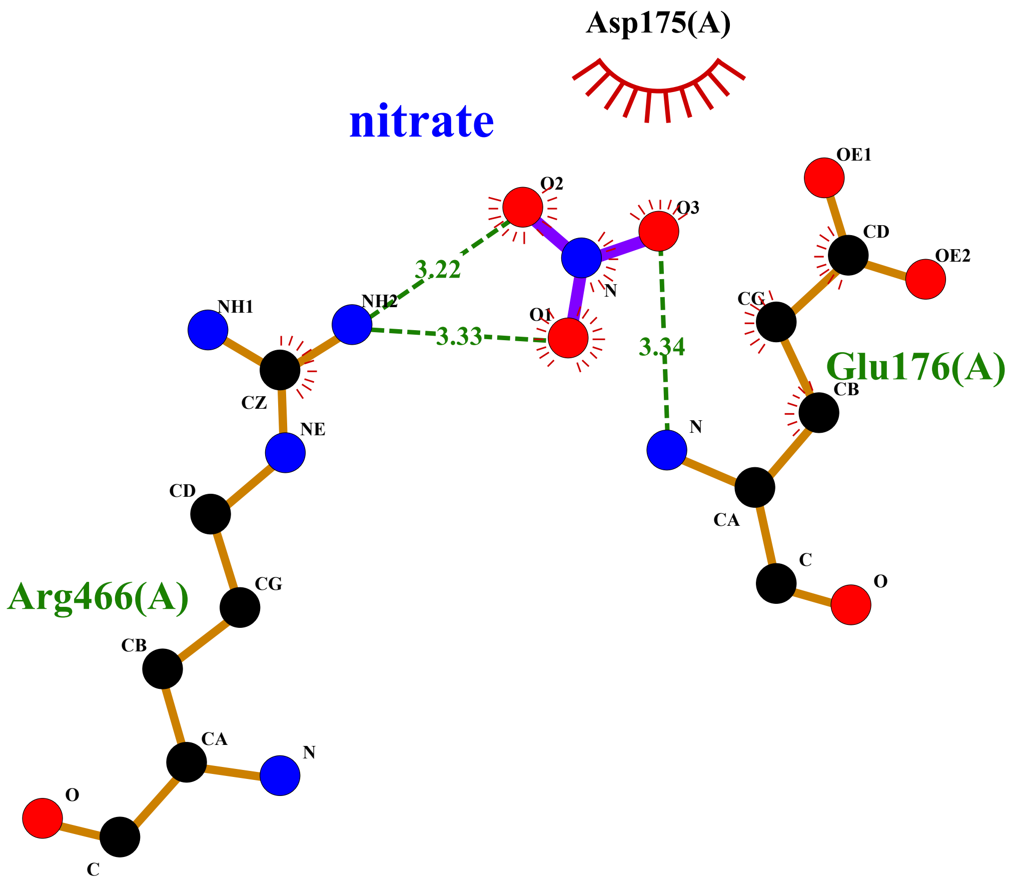
Asp175(A)
nitrate
O2
O3
N
O1
3.22
3.33
3.34
NH1
NH2
CZ
NE
CD
CG
CB
CA
N
O
C
Arg466(A)
OE1
CD
OE2
CG
Glu176(A)
CB
N
CA
C
O

Si5g39730.1
