# Supplementary material for: Genome-wide identification and characterization of the NPF genes provide new insight into low nitrogen tolerance in Setaria
Source: Front Plant Sci. 2022 Dec 14;13:1043832. doi: 10.3389/fpls.2022.1043832 (PMC9795848; doi:10.3389/fpls.2022.1043832)
Supplement: Supplementary Figure 1 — Chromosome location and distribution analysis of the SiNPF genes. Tandem duplicated genes are linked by a red curve. [file DataSheet_1.zip › Supplementary Data.zip!/Supplementary Data 3/Si5g41730.1.pdf]

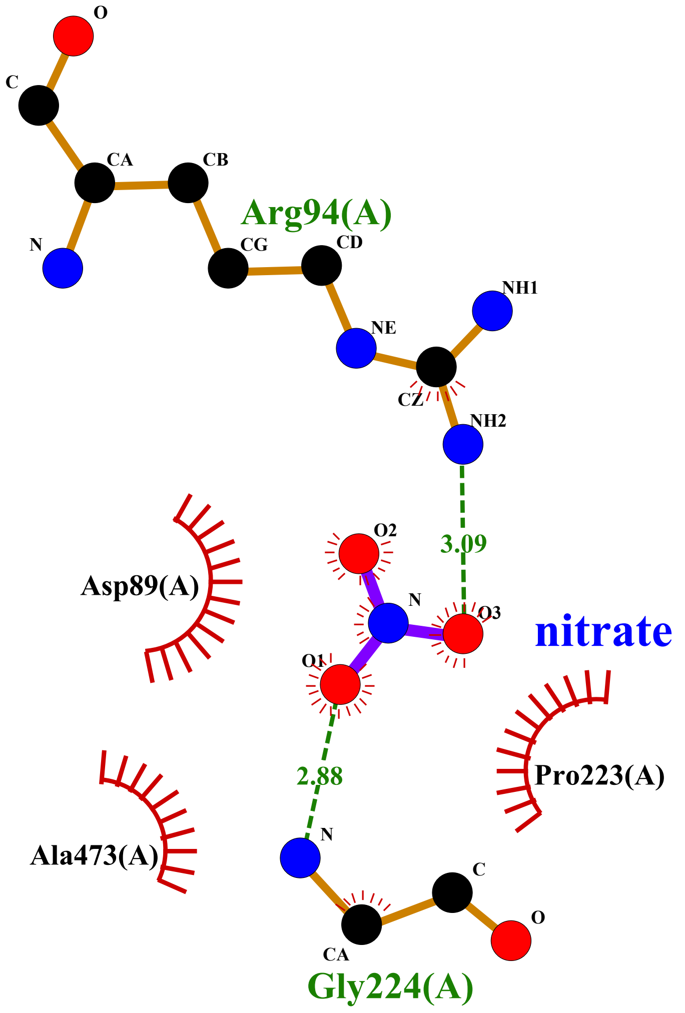

O
C
CA
CB
Arg94(A)
N
CG
CD
NH1
NE
CZ
NH2
3.09
O2
Asp89(A)
N
O3
nitrate
O1
2.88
Pro223(A)
N
Ala473(A)
C
O
CA
Gly224(A)

Si5g41730.1
