# Supplementary material for: Genome-wide identification and characterization of the NPF genes provide new insight into low nitrogen tolerance in Setaria
Source: Front Plant Sci. 2022 Dec 14;13:1043832. doi: 10.3389/fpls.2022.1043832 (PMC9795848; doi:10.3389/fpls.2022.1043832)
Supplement: Supplementary Figure 1 — Chromosome location and distribution analysis of the SiNPF genes. Tandem duplicated genes are linked by a red curve. [file DataSheet_1.zip › Supplementary Data.zip!/Supplementary Data 3/Si5g42340.1.pdf]

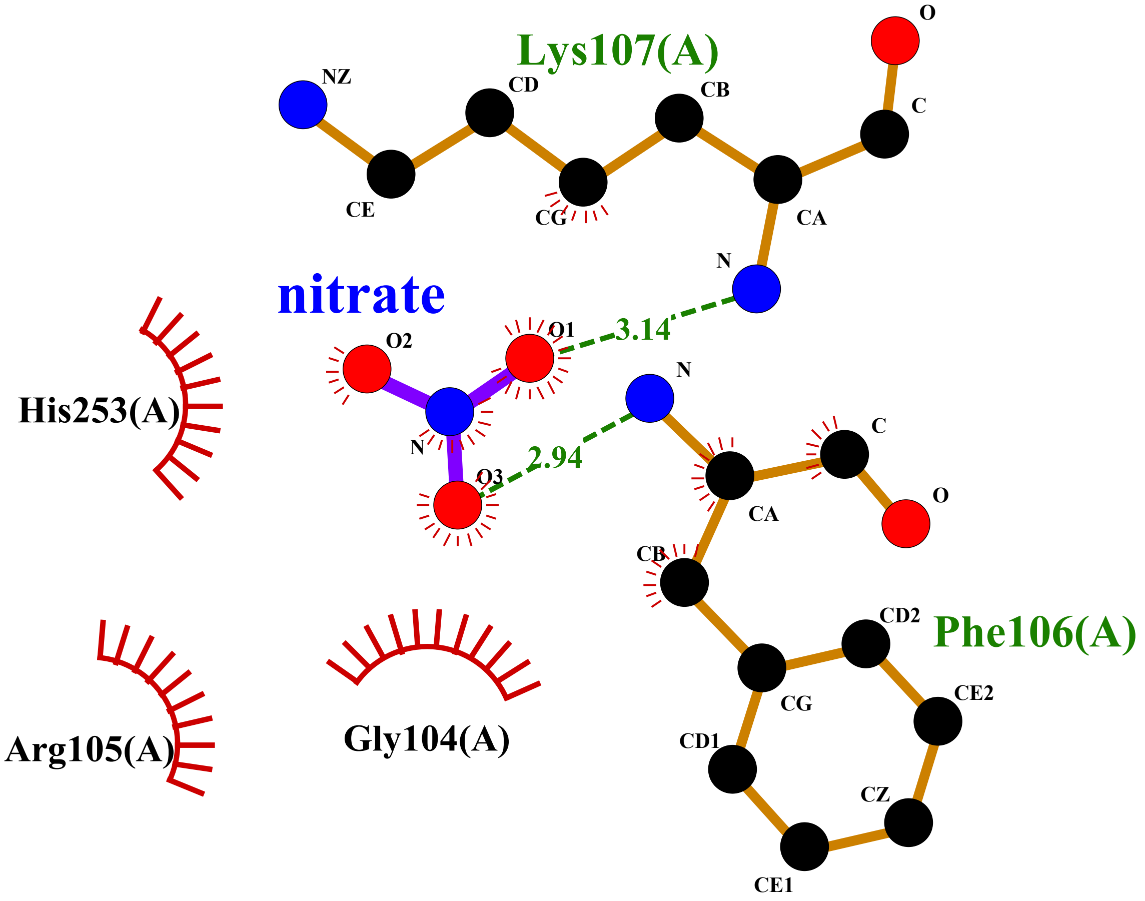
Lys107(A)
NZ
CD
CB
O
C
CE
CG
CA
N
nitrate
O2
O1
3.14
N
His253(A)
N
O3
2.94
C
CA
O
CB
CD2
Phe106(A)
CG
CE2
CD1
CZ
CE1
Arg105(A)
Gly104(A)

Si5g42340.1
