# Supplementary material for: Genome-wide identification and characterization of the NPF genes provide new insight into low nitrogen tolerance in Setaria
Source: Front Plant Sci. 2022 Dec 14;13:1043832. doi: 10.3389/fpls.2022.1043832 (PMC9795848; doi:10.3389/fpls.2022.1043832)
Supplement: Supplementary Figure 1 — Chromosome location and distribution analysis of the SiNPF genes. Tandem duplicated genes are linked by a red curve. [file DataSheet_1.zip › Supplementary Data.zip!/Supplementary Data 3/Si6g02920.2.pdf]

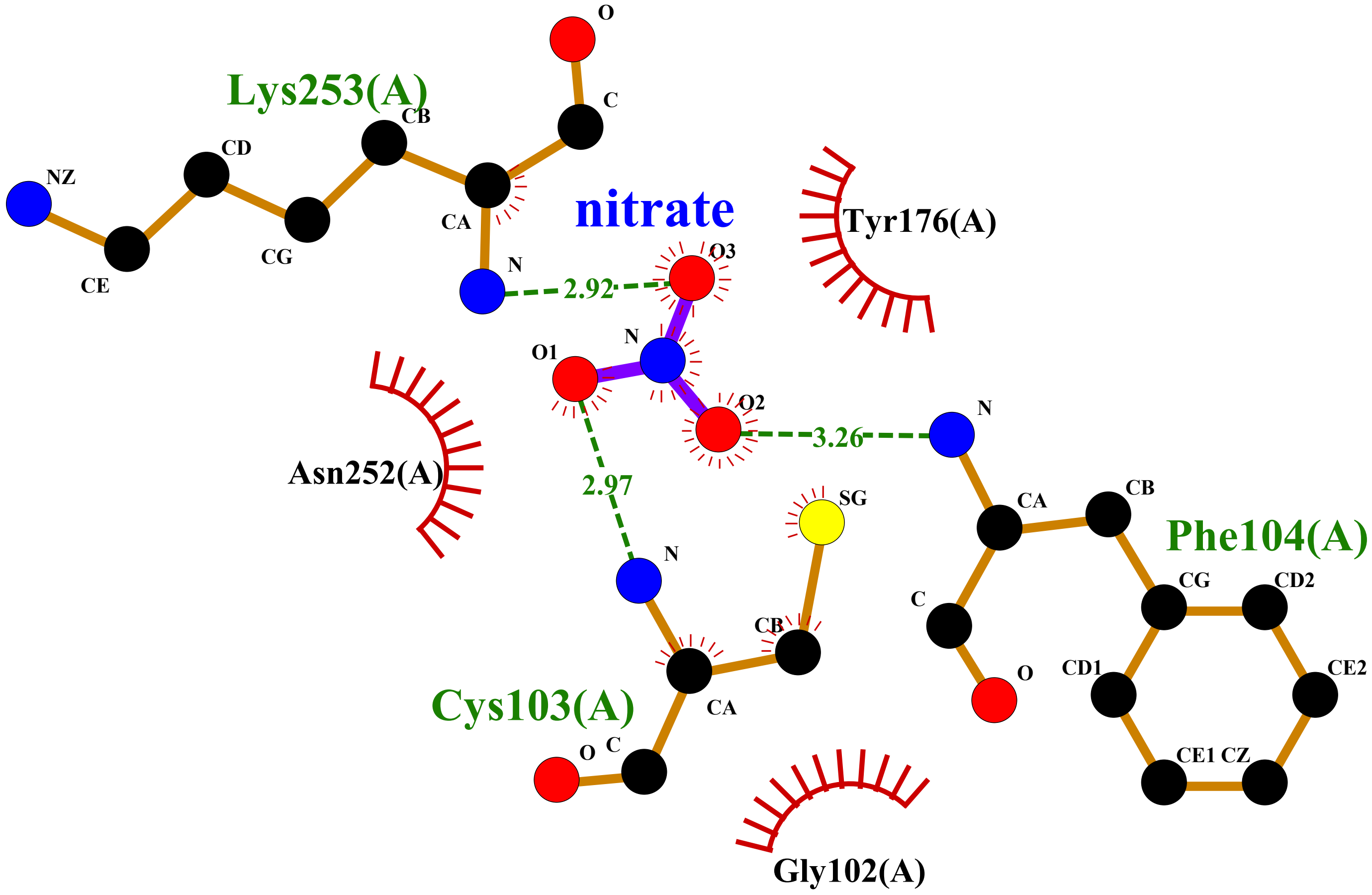
O
C
Lys253(A)
CB
CD
NZ
CA
nitrate
Tyr176(A)
CG
CE
N
2.92
O3
N
O1
O2
N
3.26
Asn252(A)
2.97
SG
CA
CB
Phe104(A)
N
CG
CD2
C
CB
O
CD1
CE2
Cys103(A)
CA
O
C
CE1 CZ
Gly102(A)

Si6g02920.2
