# Supplementary material for: Genome-wide identification and characterization of the NPF genes provide new insight into low nitrogen tolerance in Setaria
Source: Front Plant Sci. 2022 Dec 14;13:1043832. doi: 10.3389/fpls.2022.1043832 (PMC9795848; doi:10.3389/fpls.2022.1043832)
Supplement: Supplementary Figure 1 — Chromosome location and distribution analysis of the SiNPF genes. Tandem duplicated genes are linked by a red curve. [file DataSheet_1.zip › Supplementary Data.zip!/Supplementary Data 3/Si6g06290.1.pdf]

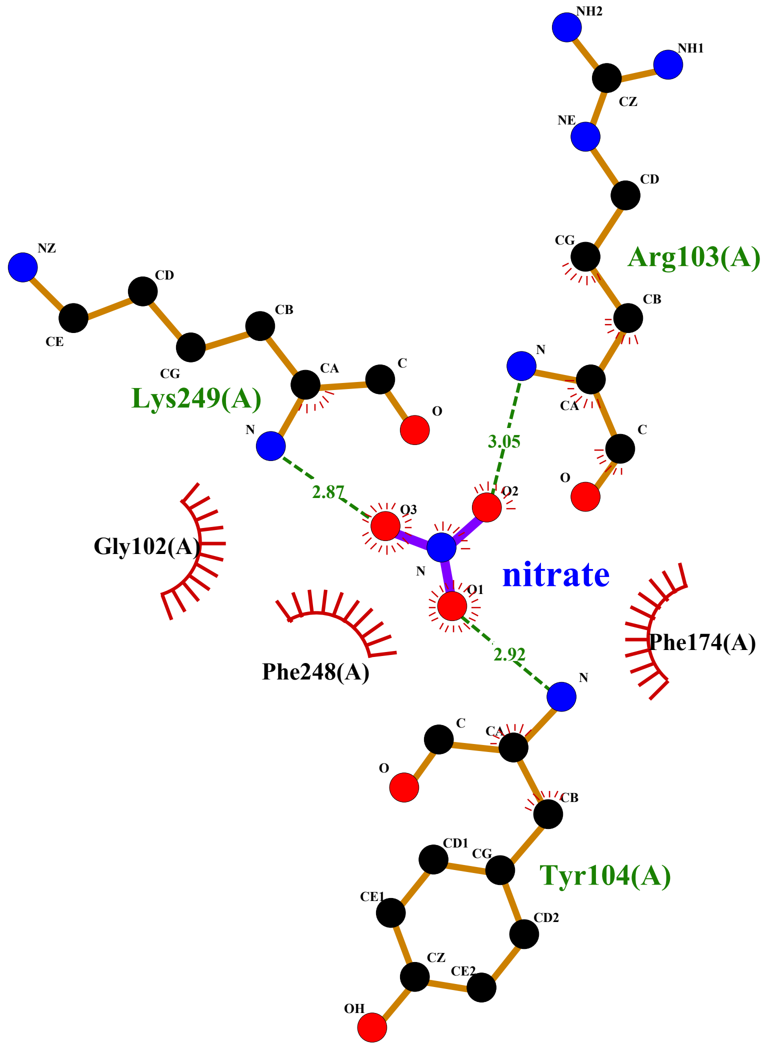
Arg103(A)
Lys249(A)
Gly102(A)
nitrate
Phe174(A)
Phe248(A)
Tyr104(A)
3.05
2.87
2.92
NH2
NH1
CZ
NE
CD
CG
CB
CA
C
O
N
NZ
CE
O1
O2
O3
CD1
CD2
CE1
CE2
OH

Si6g06290.1
