# Supplementary material for: Genome-wide identification and characterization of the NPF genes provide new insight into low nitrogen tolerance in Setaria
Source: Front Plant Sci. 2022 Dec 14;13:1043832. doi: 10.3389/fpls.2022.1043832 (PMC9795848; doi:10.3389/fpls.2022.1043832)
Supplement: Supplementary Figure 1 — Chromosome location and distribution analysis of the SiNPF genes. Tandem duplicated genes are linked by a red curve. [file DataSheet_1.zip › Supplementary Data.zip!/Supplementary Data 3/Si6g12360.1.pdf]

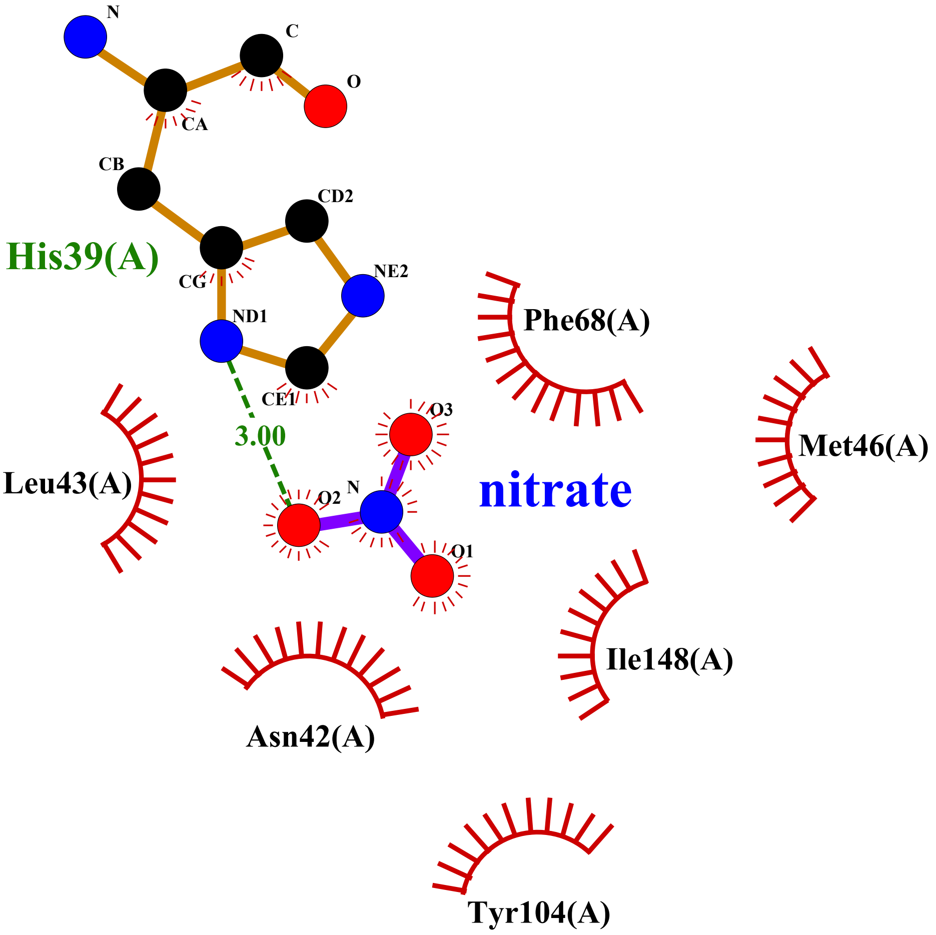
N
C
O
CA
CB
CD2
His39(A)
CG
NE2
ND1
Phe68(A)
CE1
O3
Met46(A)
3.00
Leu43(A)
O2
N
nitrate
O1
Ile148(A)
Asn42(A)
Tyr104(A)

Si6g12360.1
