# Supplementary material for: Genome-wide identification and characterization of the NPF genes provide new insight into low nitrogen tolerance in Setaria
Source: Front Plant Sci. 2022 Dec 14;13:1043832. doi: 10.3389/fpls.2022.1043832 (PMC9795848; doi:10.3389/fpls.2022.1043832)
Supplement: Supplementary Figure 1 — Chromosome location and distribution analysis of the SiNPF genes. Tandem duplicated genes are linked by a red curve. [file DataSheet_1.zip › Supplementary Data.zip!/Supplementary Data 3/Si6g12910.1.pdf]

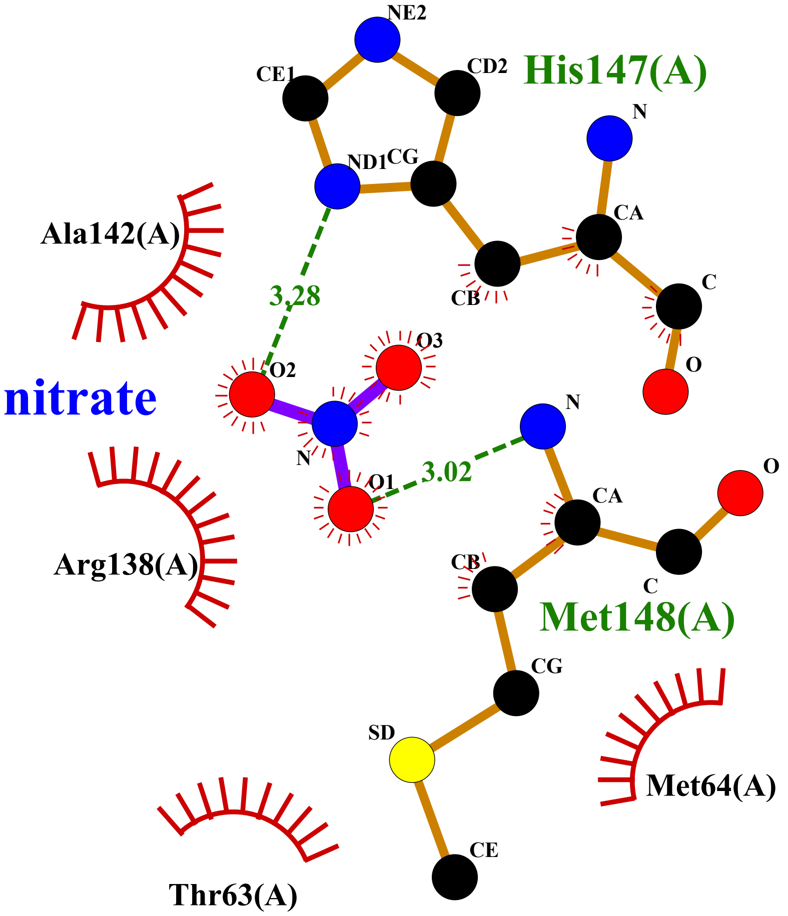
NE2
CE1
CD2
His147(A)
N
ND1
CG
Ala142(A)
CA
3.28
CB
C
O3
nitrate
O2
O
N
N
3.02
O1
O
CA
Arg138(A)
CB
C
Met148(A)
CG
SD
Met64(A)
CE
Thr63(A)

Si6g12910.1
