# Supplementary material for: Genome-wide identification and characterization of the NPF genes provide new insight into low nitrogen tolerance in Setaria
Source: Front Plant Sci. 2022 Dec 14;13:1043832. doi: 10.3389/fpls.2022.1043832 (PMC9795848; doi:10.3389/fpls.2022.1043832)
Supplement: Supplementary Figure 1 — Chromosome location and distribution analysis of the SiNPF genes. Tandem duplicated genes are linked by a red curve. [file DataSheet_1.zip › Supplementary Data.zip!/Supplementary Data 3/Si7g12260.1.pdf]

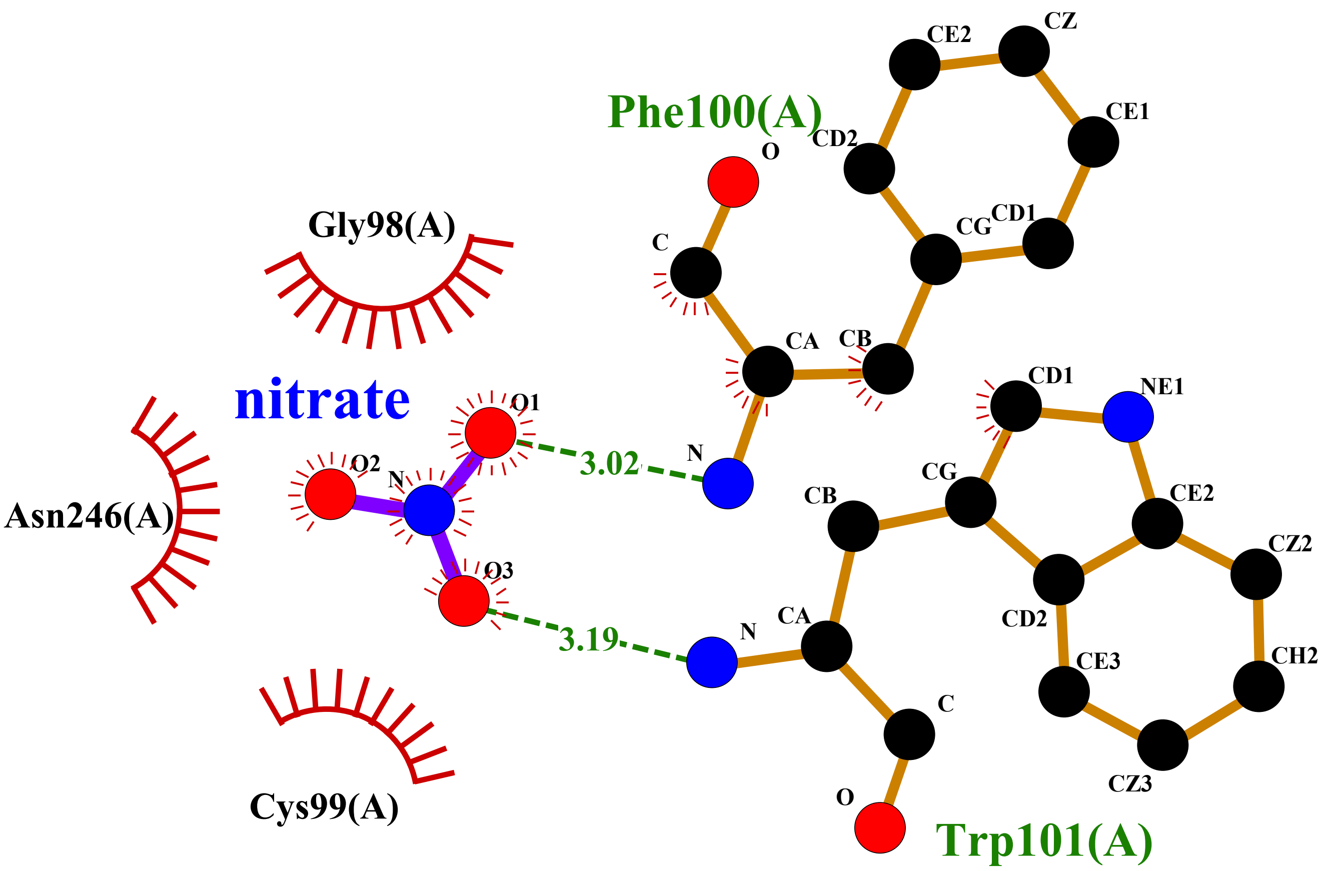
Phe100(A)
O
C
CA
CB
CD2
CE2
CZ
CE1
CD1
CG
N
Gly98(A)
nitrate
O1
O2
N
O3
Asn246(A)
3.02
3.19
Cys99(A)
CD1
NE1
CG
CB
CE2
CZ2
CD2
CA
N
CE3
CH2
C
CZ3
O
Trp101(A)

Si7g12260.1
