# Supplementary material for: Genome-wide identification and characterization of the NPF genes provide new insight into low nitrogen tolerance in Setaria
Source: Front Plant Sci. 2022 Dec 14;13:1043832. doi: 10.3389/fpls.2022.1043832 (PMC9795848; doi:10.3389/fpls.2022.1043832)
Supplement: Supplementary Figure 1 — Chromosome location and distribution analysis of the SiNPF genes. Tandem duplicated genes are linked by a red curve. [file DataSheet_1.zip › Supplementary Data.zip!/Supplementary Data 3/Si7g16260.1.pdf]

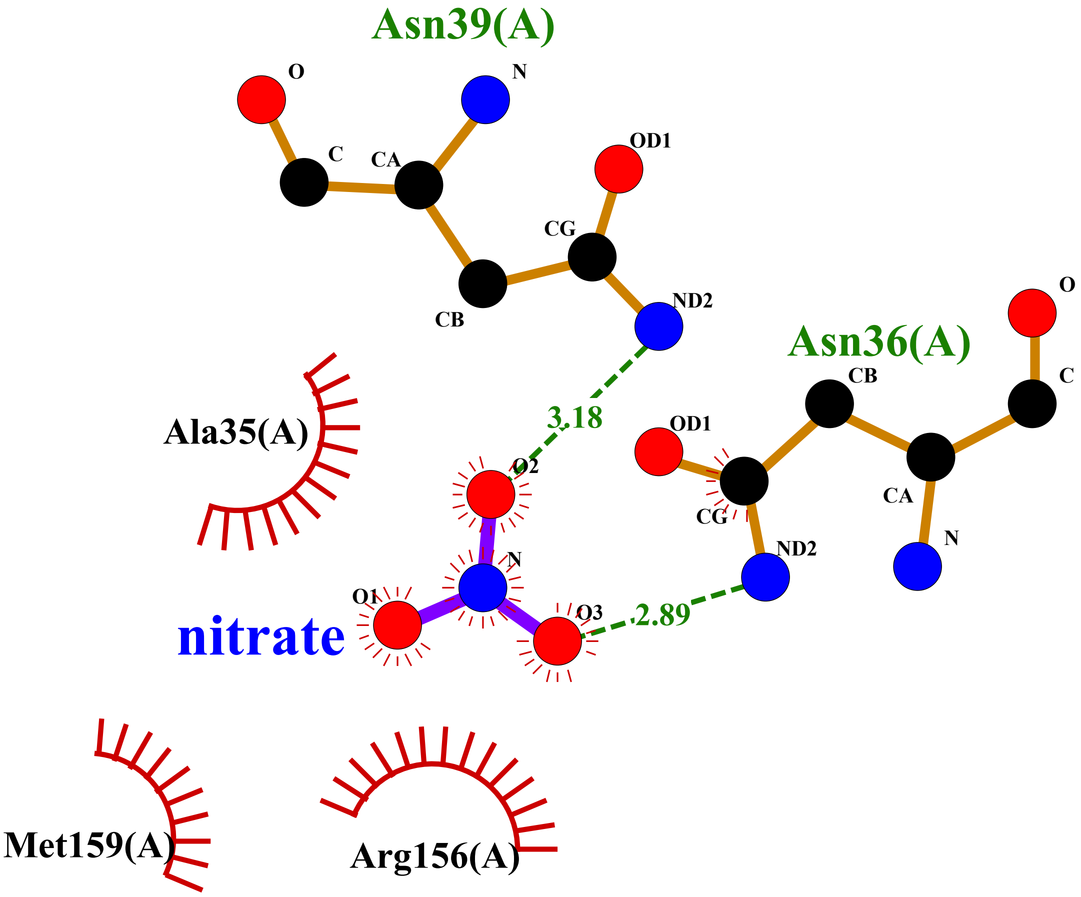
Asn39(A)
O
N
C
CA
OD1
CG
CB
ND2
O
Asn36(A)
CB
C
Ala35(A)
3.18
OD1
O2
CA
CG
ND2
N
N
O1
O3
2.89
nitrate
Met159(A)
Arg156(A)

Si7g16260.1
