# Supplementary material for: Genome-wide identification and characterization of the NPF genes provide new insight into low nitrogen tolerance in Setaria
Source: Front Plant Sci. 2022 Dec 14;13:1043832. doi: 10.3389/fpls.2022.1043832 (PMC9795848; doi:10.3389/fpls.2022.1043832)
Supplement: Supplementary Figure 1 — Chromosome location and distribution analysis of the SiNPF genes. Tandem duplicated genes are linked by a red curve. [file DataSheet_1.zip › Supplementary Data.zip!/Supplementary Data 3/Si7g23220.1.pdf]

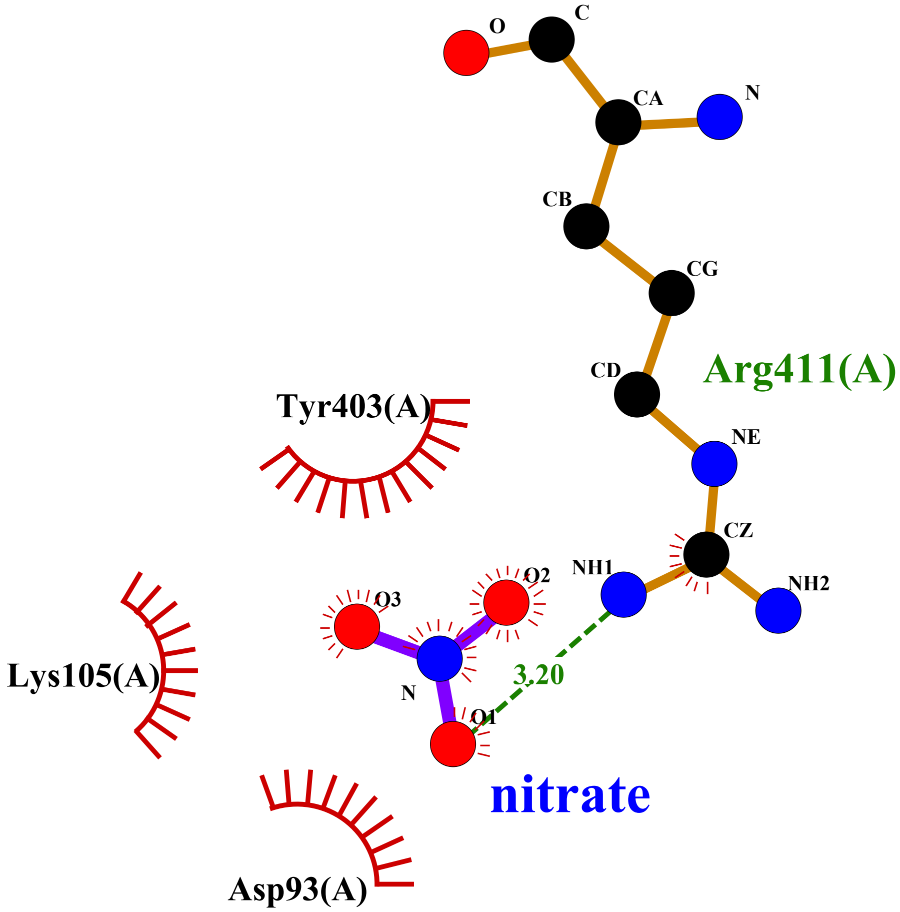
O
C
CA
N
CB
CG
CD
Arg411(A)
Tyr403(A)
NE
CZ
NH1
NH2
O3
O2
Lys105(A)
N
3.20
O1
nitrate
Asp93(A)

Si7g23220.1
