# Supplementary material for: Genome-wide identification and characterization of the NPF genes provide new insight into low nitrogen tolerance in Setaria
Source: Front Plant Sci. 2022 Dec 14;13:1043832. doi: 10.3389/fpls.2022.1043832 (PMC9795848; doi:10.3389/fpls.2022.1043832)
Supplement: Supplementary Figure 1 — Chromosome location and distribution analysis of the SiNPF genes. Tandem duplicated genes are linked by a red curve. [file DataSheet_1.zip › Supplementary Data.zip!/Supplementary Data 3/Si7g23230.1.pdf]

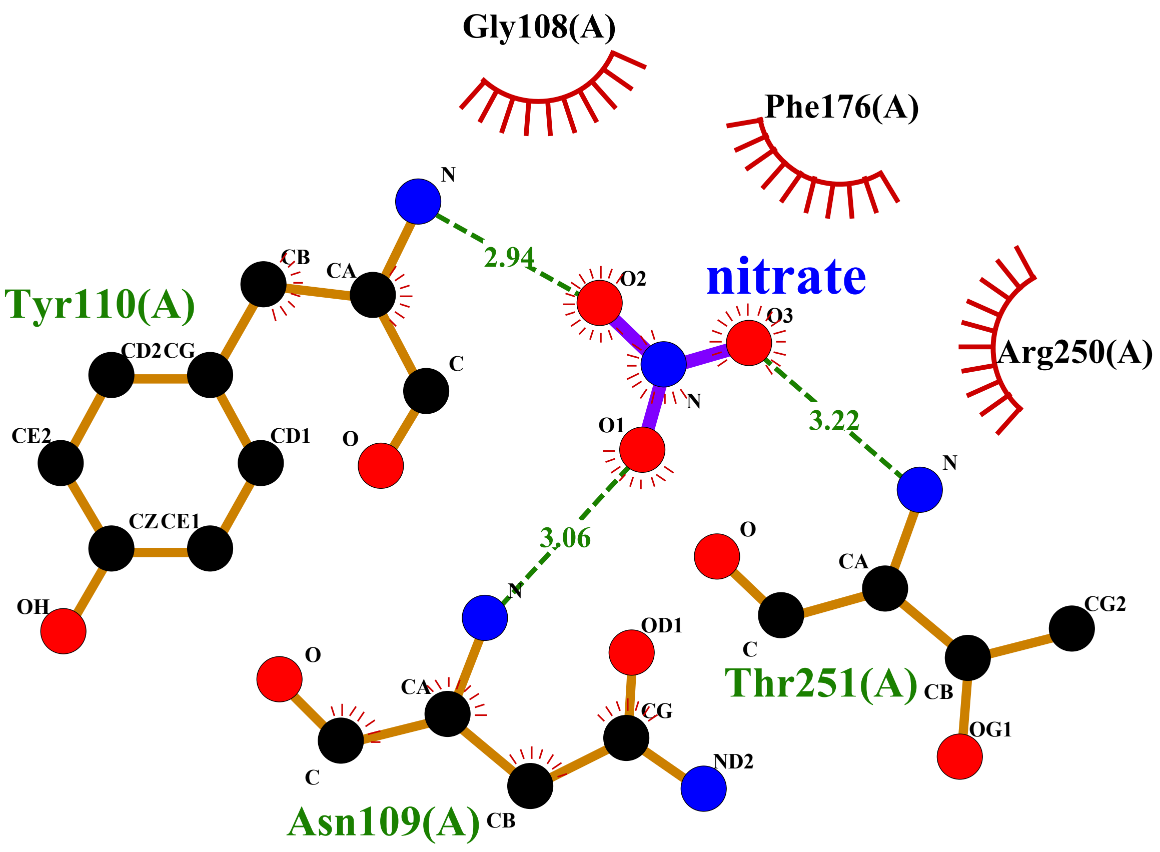

Gly108(A)
Phe176(A)
nitrate
Arg250(A)
Tyr110(A)
N
CB
CA
C
O
CD2
CG
CE2
CD1
CZ
CE1
OH
2.94
O2
O3
N
O1
3.22
3.06
N
O
CA
C
CG2
CB
OG1
Thr251(A)
N
O
CA
C
OD1
CG
ND2
CB
Asn109(A)

Si7g23230.1
