# Supplementary material for: Genome-wide identification and characterization of the NPF genes provide new insight into low nitrogen tolerance in Setaria
Source: Front Plant Sci. 2022 Dec 14;13:1043832. doi: 10.3389/fpls.2022.1043832 (PMC9795848; doi:10.3389/fpls.2022.1043832)
Supplement: Supplementary Figure 1 — Chromosome location and distribution analysis of the SiNPF genes. Tandem duplicated genes are linked by a red curve. [file DataSheet_1.zip › Supplementary Data.zip!/Supplementary Data 3/Si8g08880.1.pdf]

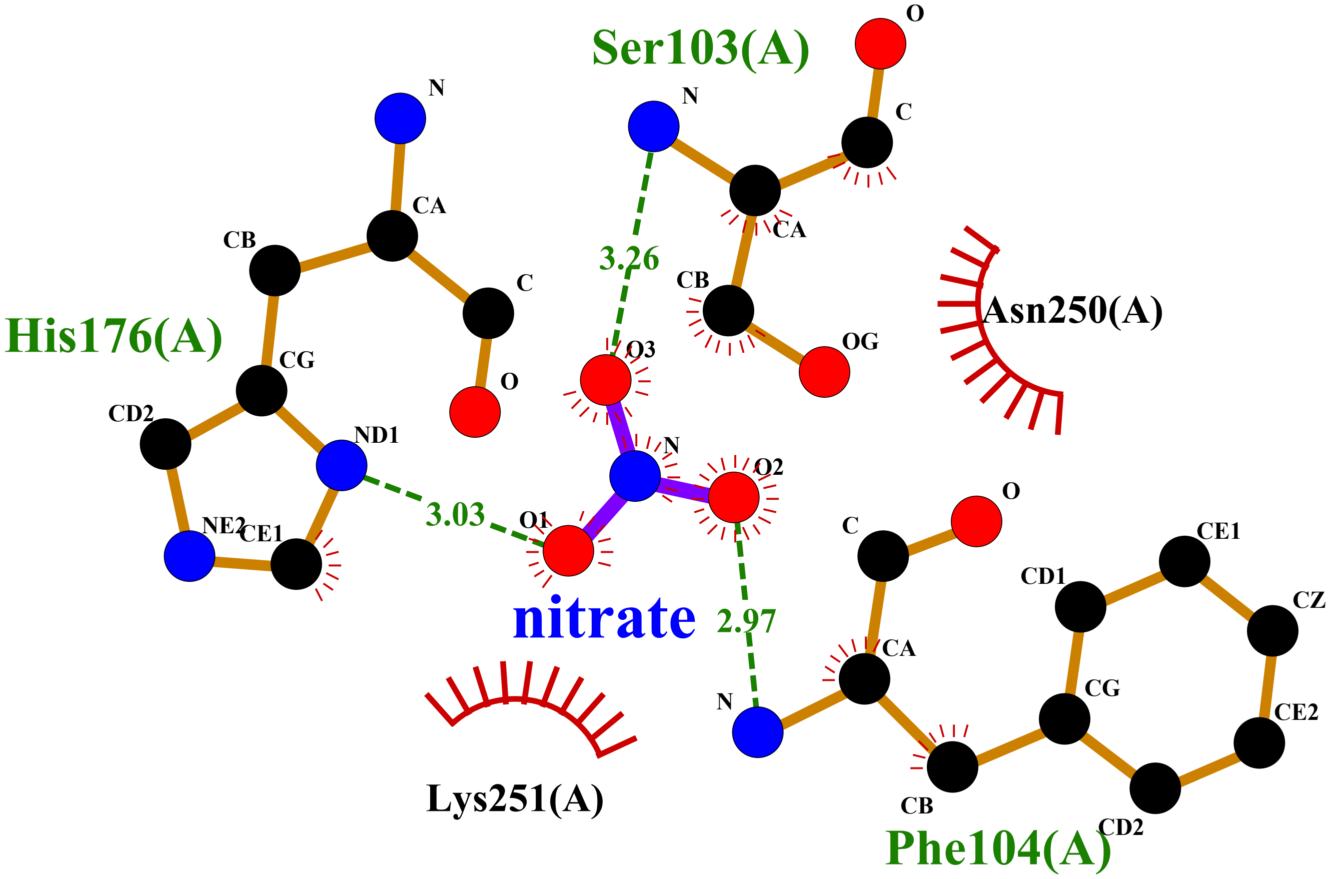
Ser103(A)
His176(A)
Asn250(A)
nitrate
Lys251(A)
Phe104(A)
3.26
3.03
2.97
N
CA
CB
C
O
CG
CD2
ND1
NE2
CE1
OG
O1
O2
O3
CD1
CE2
CZ

Si8g08880.1
