# Supplementary material for: Genome-wide identification and characterization of the NPF genes provide new insight into low nitrogen tolerance in Setaria
Source: Front Plant Sci. 2022 Dec 14;13:1043832. doi: 10.3389/fpls.2022.1043832 (PMC9795848; doi:10.3389/fpls.2022.1043832)
Supplement: Supplementary Figure 1 — Chromosome location and distribution analysis of the SiNPF genes. Tandem duplicated genes are linked by a red curve. [file DataSheet_1.zip › Supplementary Data.zip!/Supplementary Data 3/Si8g10670.1.pdf]

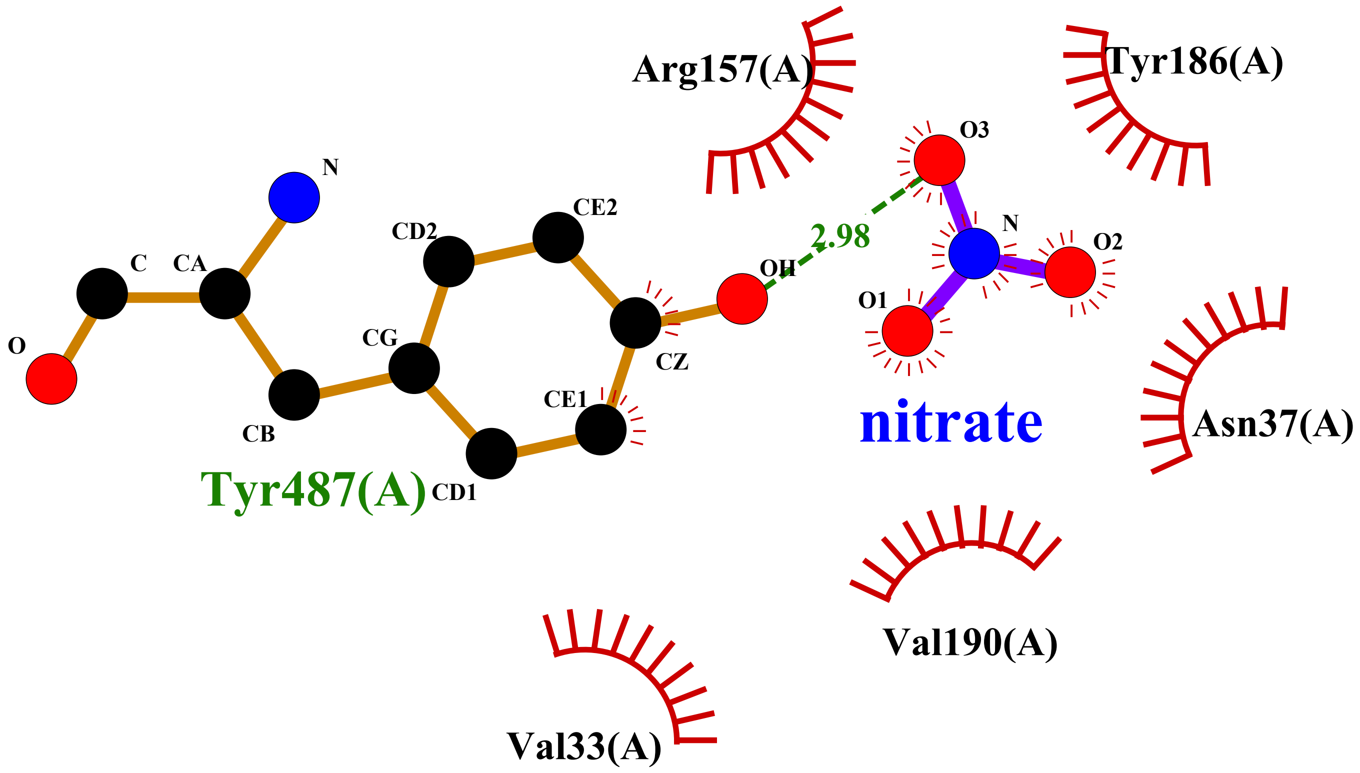
Arg157(A)
Tyr186(A)
O3
N
CE2
CD2
2.98
N
O2
C
CA
OH
O1
O
CG
CZ
CE1
nitrate
Asn37(A)
CB
Tyr487(A)
CD1
Val190(A)
Val33(A)

Si8g10670.1
