# Supplementary material for: Genome-wide identification and characterization of the NPF genes provide new insight into low nitrogen tolerance in Setaria
Source: Front Plant Sci. 2022 Dec 14;13:1043832. doi: 10.3389/fpls.2022.1043832 (PMC9795848; doi:10.3389/fpls.2022.1043832)
Supplement: Supplementary Figure 1 — Chromosome location and distribution analysis of the SiNPF genes. Tandem duplicated genes are linked by a red curve. [file DataSheet_1.zip › Supplementary Data.zip!/Supplementary Data 3/Si8g11510.1.pdf]

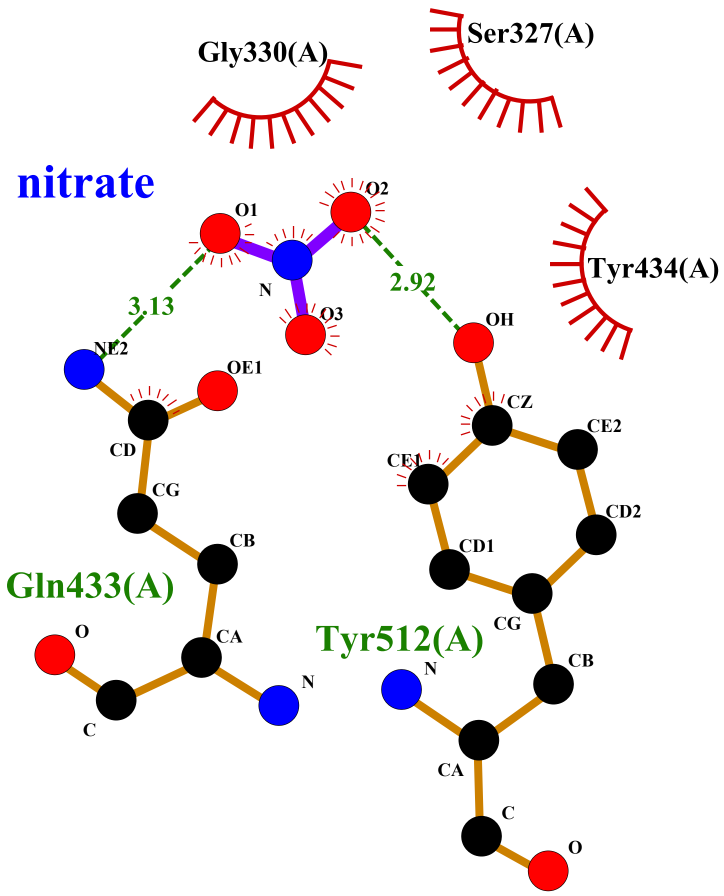
Gly330(A)
Ser327(A)
nitrate
O1
O2
N
O3
3.13
2.92
Tyr434(A)
NE2
OE1
OH
CD
CZ
CE2
CE1
CG
CD2
CB
CD1
Gln433(A)
O
CA
Tyr512(A)
CG
CB
N
N
C
CA
C
O

Si8g11510.1
