# Supplementary material for: Genome-wide identification and characterization of the NPF genes provide new insight into low nitrogen tolerance in Setaria
Source: Front Plant Sci. 2022 Dec 14;13:1043832. doi: 10.3389/fpls.2022.1043832 (PMC9795848; doi:10.3389/fpls.2022.1043832)
Supplement: Supplementary Figure 1 — Chromosome location and distribution analysis of the SiNPF genes. Tandem duplicated genes are linked by a red curve. [file DataSheet_1.zip › Supplementary Data.zip!/Supplementary Data 3/Si8g11640.1.pdf]

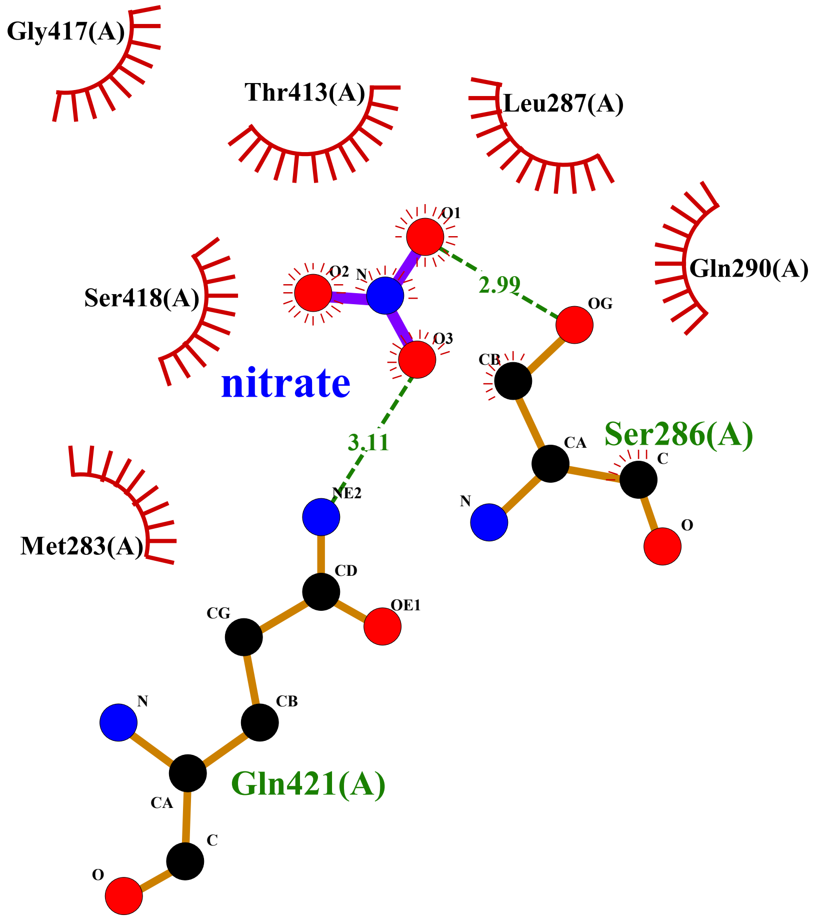
Gly417(A)
Thr413(A)
Leu287(A)
Gln290(A)
Ser418(A)
O1
O2
N
O3
2.99
OG
CB
nitrate
3.11
CA
Ser286(A)
C
N
O
NE2
Met283(A)
CD
CG
OE1
N
CB
CA
Gln421(A)
C
O

Si8g11640.1
