# Supplementary material for: Genome-wide identification and characterization of the NPF genes provide new insight into low nitrogen tolerance in Setaria
Source: Front Plant Sci. 2022 Dec 14;13:1043832. doi: 10.3389/fpls.2022.1043832 (PMC9795848; doi:10.3389/fpls.2022.1043832)
Supplement: Supplementary Figure 1 — Chromosome location and distribution analysis of the SiNPF genes. Tandem duplicated genes are linked by a red curve. [file DataSheet_1.zip › Supplementary Data.zip!/Supplementary Data 3/Si9g03840.1.pdf]

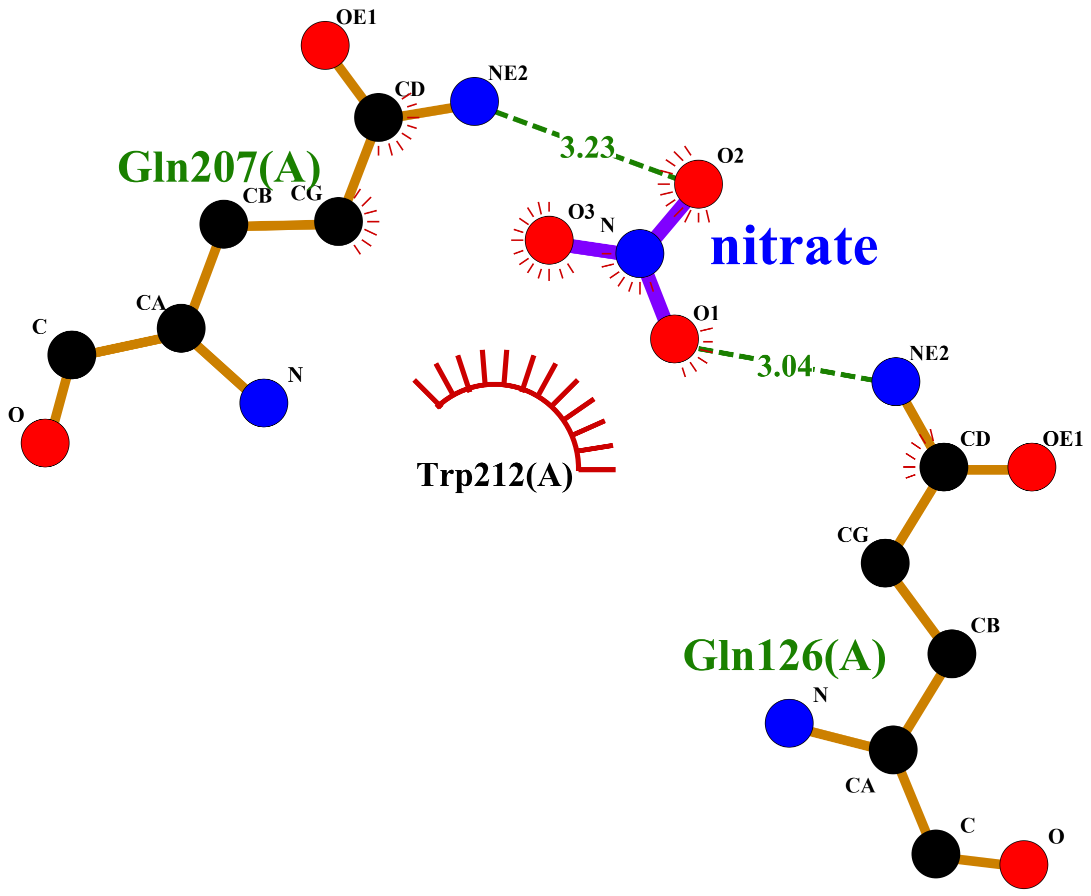

OE1
CD
NE2
3.23
O2
Gln207(A)
CB
CG
O3
N
nitrate
CA
O1
C
3.04
NE2
N
O
CD
OE1
Trp212(A)
CG
CB
Gln126(A)
N
CA
C
O

Si9g03840.1
