# Supplementary material for: Genome-wide identification and characterization of the NPF genes provide new insight into low nitrogen tolerance in Setaria
Source: Front Plant Sci. 2022 Dec 14;13:1043832. doi: 10.3389/fpls.2022.1043832 (PMC9795848; doi:10.3389/fpls.2022.1043832)
Supplement: Supplementary Figure 1 — Chromosome location and distribution analysis of the SiNPF genes. Tandem duplicated genes are linked by a red curve. [file DataSheet_1.zip › Supplementary Data.zip!/Supplementary Data 3/Si9g10280.1.pdf]

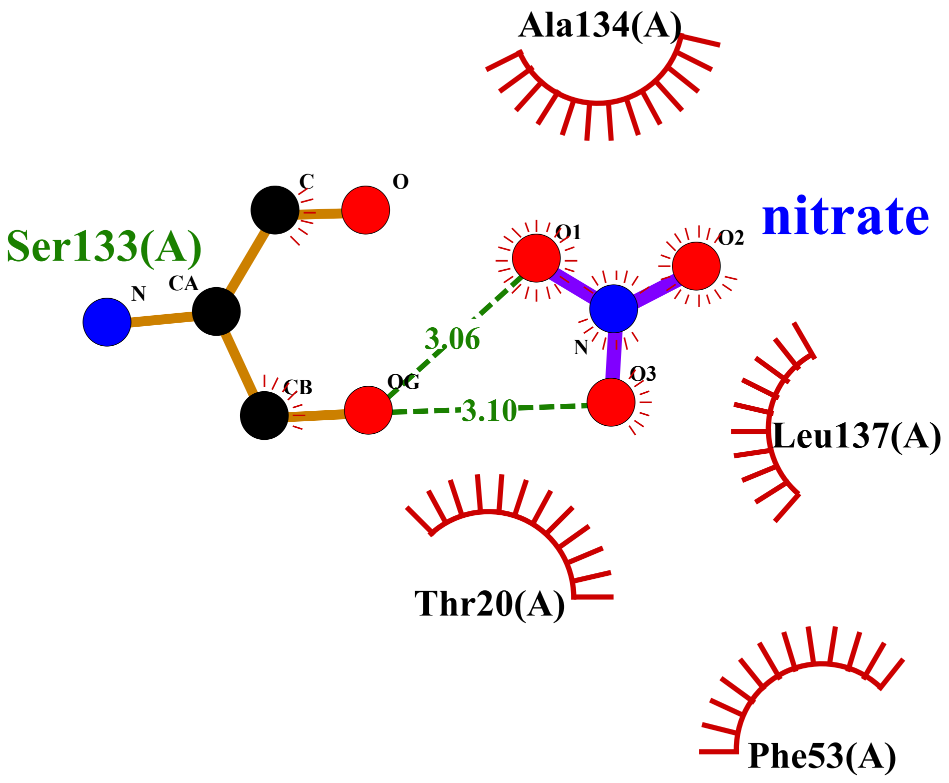
Ala134(A)
nitrate
Ser133(A)
C
O
N
CA
O1
O2
N
O3
CB
OG
3.06
3.10
Leu137(A)
Thr20(A)
Phe53(A)

Si9g10280.1
