# Supplementary material for: Genome-wide identification and characterization of the NPF genes provide new insight into low nitrogen tolerance in Setaria
Source: Front Plant Sci. 2022 Dec 14;13:1043832. doi: 10.3389/fpls.2022.1043832 (PMC9795848; doi:10.3389/fpls.2022.1043832)
Supplement: Supplementary Figure 1 — Chromosome location and distribution analysis of the SiNPF genes. Tandem duplicated genes are linked by a red curve. [file DataSheet_1.zip › Supplementary Data.zip!/Supplementary Data 3/Si9g11400.1.pdf]

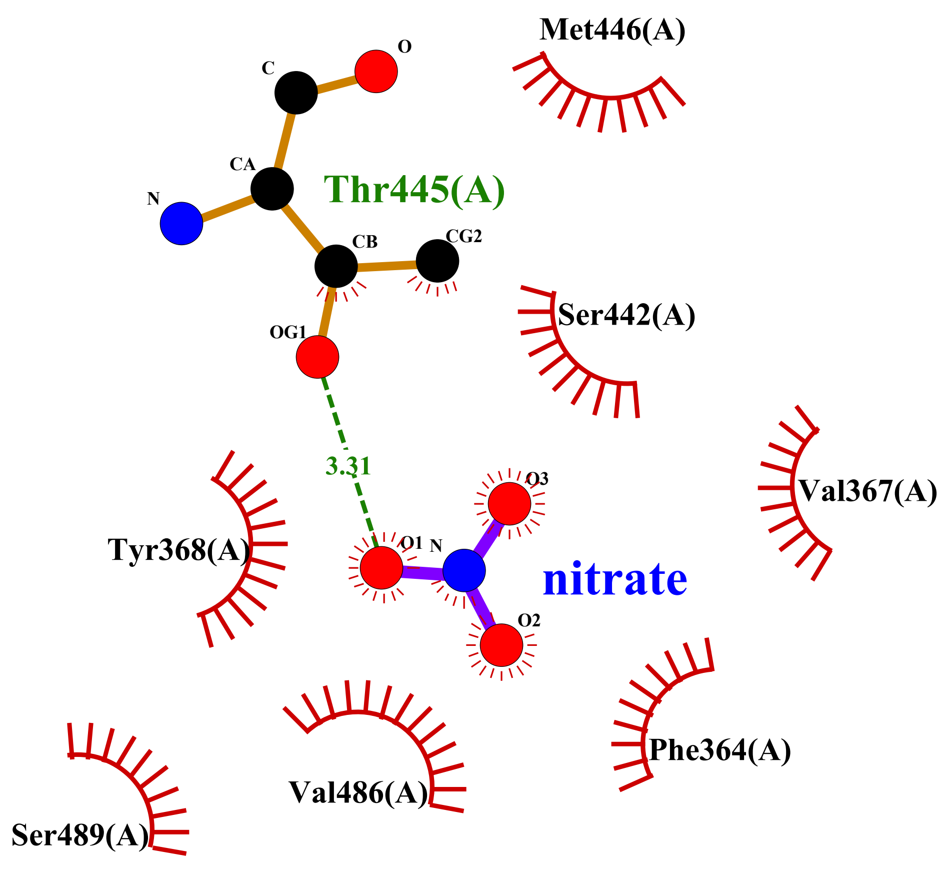
Met446(A)
C
O
CA
N
Thr445(A)
CB
CG2
OG1
Ser442(A)
Val367(A)
3.31
O3
Tyr368(A)
O1
N
nitrate
O2
Phe364(A)
Val486(A)
Ser489(A)

Si9g11400.1
