# Supplementary material for: Genome-wide identification and characterization of the NPF genes provide new insight into low nitrogen tolerance in Setaria
Source: Front Plant Sci. 2022 Dec 14;13:1043832. doi: 10.3389/fpls.2022.1043832 (PMC9795848; doi:10.3389/fpls.2022.1043832)
Supplement: Supplementary Figure 1 — Chromosome location and distribution analysis of the SiNPF genes. Tandem duplicated genes are linked by a red curve. [file DataSheet_1.zip › Supplementary Data.zip!/Supplementary Data 3/Si9g13430.1.pdf]

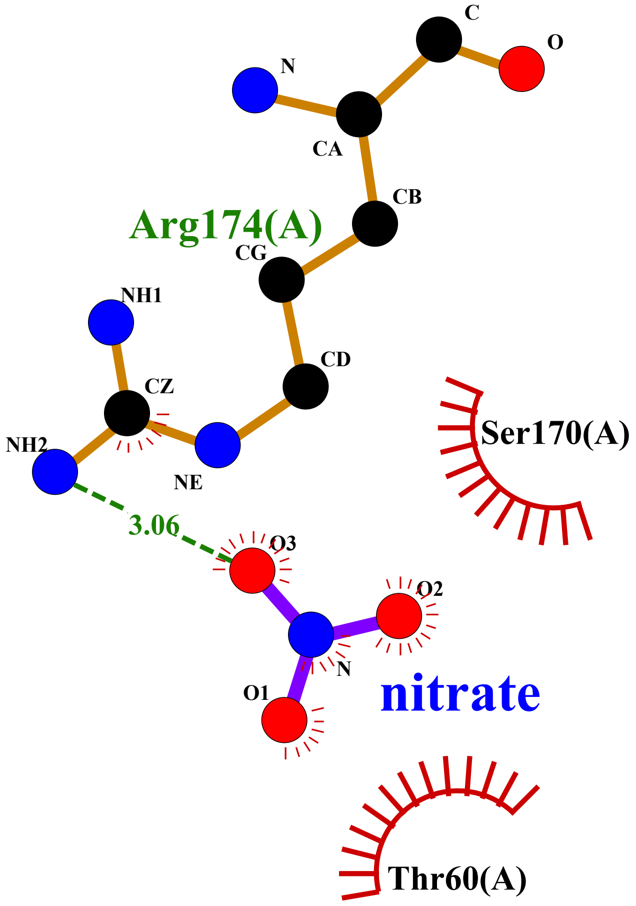
C
O
N
CA
CB
Arg174(A)
CG
NH1
CD
CZ
Ser170(A)
NH2
NE
3.06
O3
O2
N
O1
nitrate
Thr60(A)

Si9g13430.1
