# Supplementary material for: Genome-wide identification and characterization of the NPF genes provide new insight into low nitrogen tolerance in Setaria
Source: Front Plant Sci. 2022 Dec 14;13:1043832. doi: 10.3389/fpls.2022.1043832 (PMC9795848; doi:10.3389/fpls.2022.1043832)
Supplement: Supplementary Figure 1 — Chromosome location and distribution analysis of the SiNPF genes. Tandem duplicated genes are linked by a red curve. [file DataSheet_1.zip › Supplementary Data.zip!/Supplementary Data 3/Si9g22120.1.pdf]

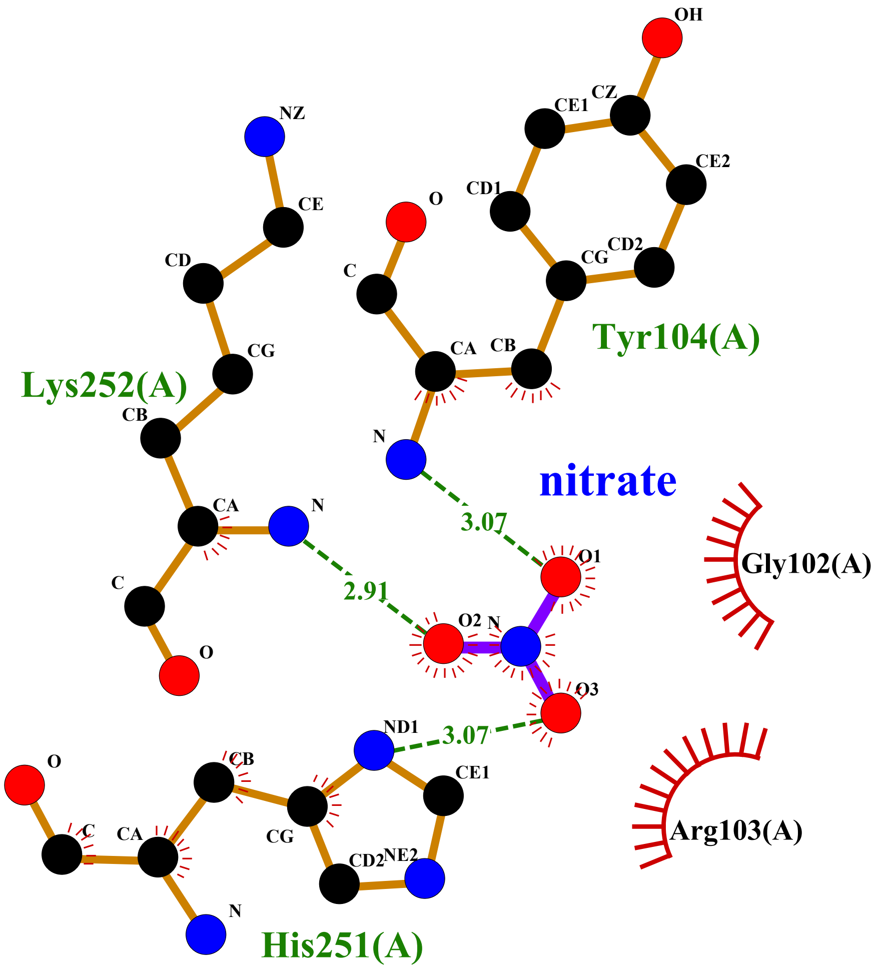
nitrate
Tyr104(A)
Lys252(A)
His251(A)
Gly102(A)
Arg103(A)
3.07
2.91
3.07
OH
CZ
CE1
CE2
CD1
CD2
CG
CA
CB
N
O
C
NZ
CE
CD
CB
O1
O2
O3
ND1
NE2
CD2
CE1

Si9g22120.1
