# Supplementary material for: Genome-wide identification and characterization of the NPF genes provide new insight into low nitrogen tolerance in Setaria
Source: Front Plant Sci. 2022 Dec 14;13:1043832. doi: 10.3389/fpls.2022.1043832 (PMC9795848; doi:10.3389/fpls.2022.1043832)
Supplement: Supplementary Figure 1 — Chromosome location and distribution analysis of the SiNPF genes. Tandem duplicated genes are linked by a red curve. [file DataSheet_1.zip › Supplementary Data.zip!/Supplementary Data 3/Si9g22140.1.pdf]

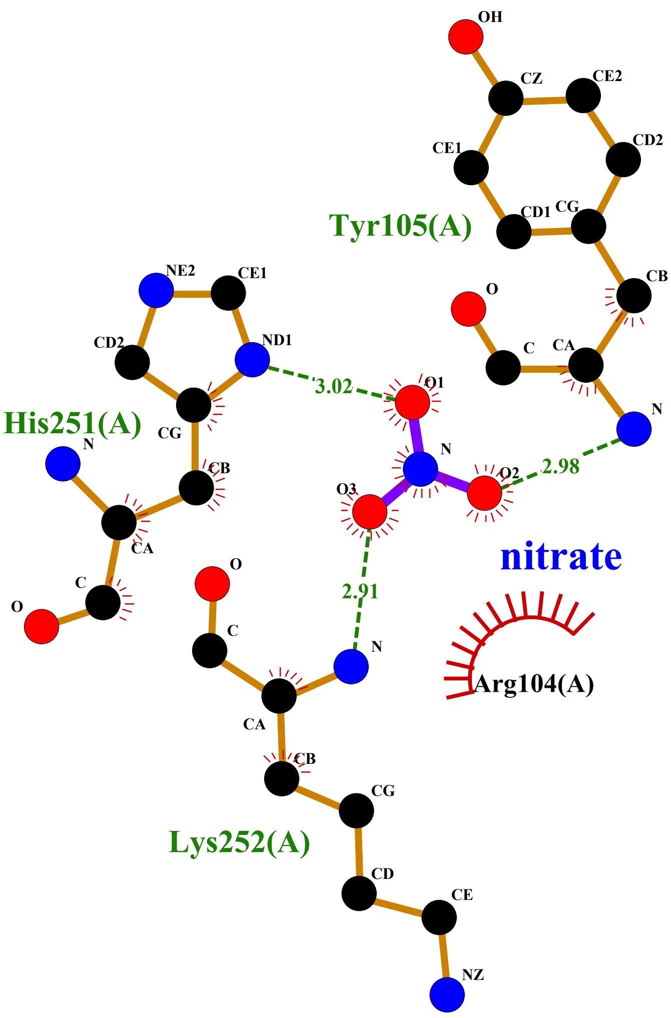
OH
CZ
CE2
CE1
CD2
CD1
CG
Tyr105(A)
NE2
CE1
O
CB
ND1
C
CA
CD2
3.02
O1
His251(A)
CG
N
N
N
CB
O3
O2
2.98
CA
nitrate
O
C
2.91
O
C
N
Arg104(A)
CA
CB
CG
Lys252(A)
CD
CE
NZ

Si9g22140.1
