# Supplementary material for: Genome-wide identification and characterization of the NPF genes provide new insight into low nitrogen tolerance in Setaria
Source: Front Plant Sci. 2022 Dec 14;13:1043832. doi: 10.3389/fpls.2022.1043832 (PMC9795848; doi:10.3389/fpls.2022.1043832)
Supplement: Supplementary Figure 1 — Chromosome location and distribution analysis of the SiNPF genes. Tandem duplicated genes are linked by a red curve. [file DataSheet_1.zip › Supplementary Data.zip!/Supplementary Data 3/Si9g29380.3.pdf]

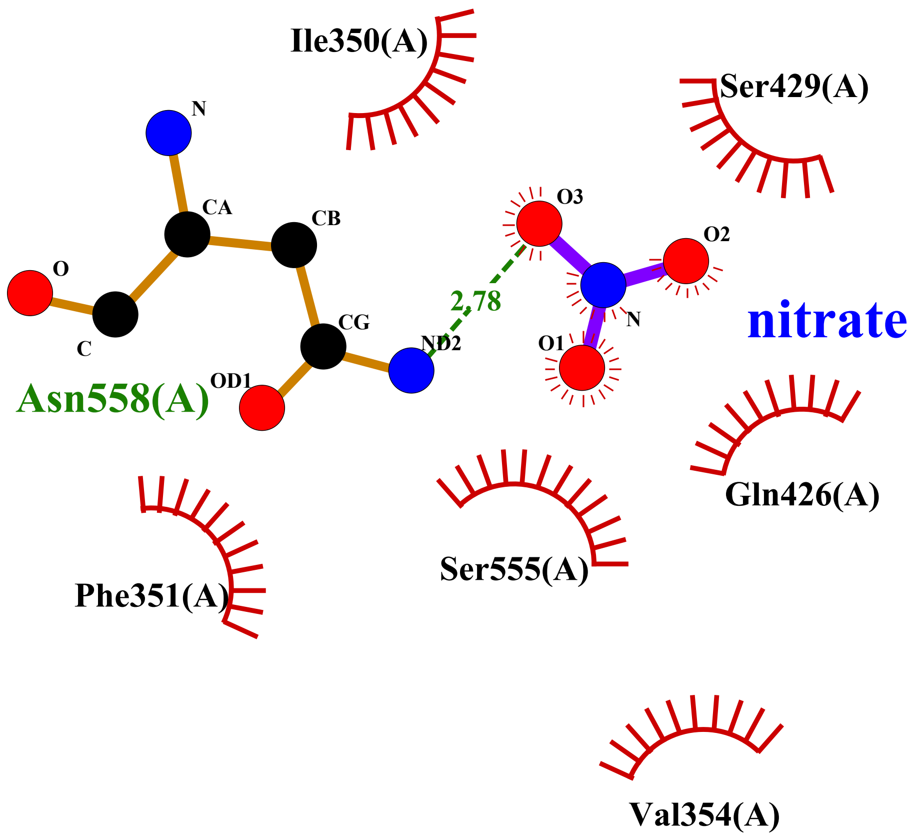

Ile350(A)
Ser429(A)
N
CA
CB
O3
O2
O
2.78
N
nitrate
C
CG
ND2
O1
Asn558(A)
OD1
Gln426(A)
Ser555(A)
Phe351(A)
Val354(A)

Si9g29380.3
