# Supplementary material for: Genome-wide identification and characterization of the NPF genes provide new insight into low nitrogen tolerance in Setaria
Source: Front Plant Sci. 2022 Dec 14;13:1043832. doi: 10.3389/fpls.2022.1043832 (PMC9795848; doi:10.3389/fpls.2022.1043832)
Supplement: Supplementary Figure 1 — Chromosome location and distribution analysis of the SiNPF genes. Tandem duplicated genes are linked by a red curve. [file DataSheet_1.zip › Supplementary Data.zip!/Supplementary Data 3/Si9g29420.1.pdf]

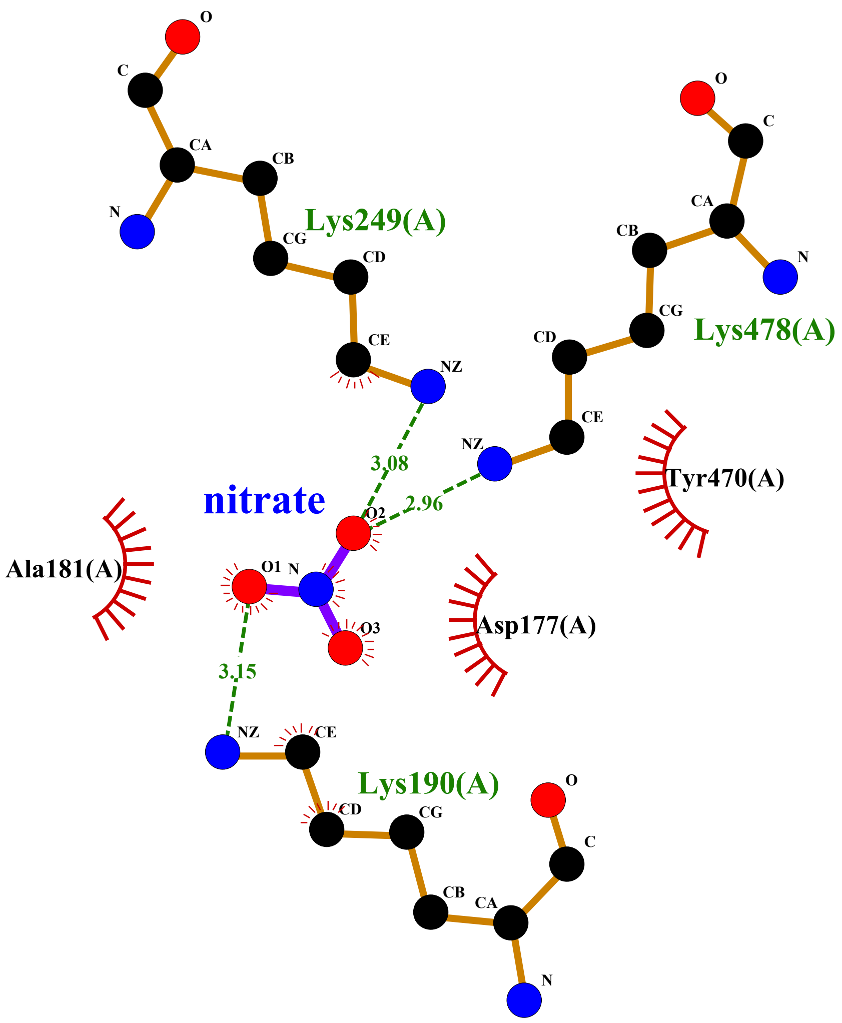

O
C
CA
CB
N
Lys249(A)
CG
CD
CE
NZ
O
C
CA
CB
N
CG
Lys478(A)
CD
CE
NZ
3.08
2.96
nitrate
Tyr470(A)
O2
Ala181(A)
O1
N
O3
Asp177(A)
3.15
NZ
CE
Lys190(A)
CD
CG
O
C
CB
CA
N

Si9g29420.1
