# Supplementary material for: Genome-wide identification and characterization of the NPF genes provide new insight into low nitrogen tolerance in Setaria
Source: Front Plant Sci. 2022 Dec 14;13:1043832. doi: 10.3389/fpls.2022.1043832 (PMC9795848; doi:10.3389/fpls.2022.1043832)
Supplement: Supplementary Figure 1 — Chromosome location and distribution analysis of the SiNPF genes. Tandem duplicated genes are linked by a red curve. [file DataSheet_1.zip › Supplementary Data.zip!/Supplementary Data 3/Si9g29460.1.pdf]

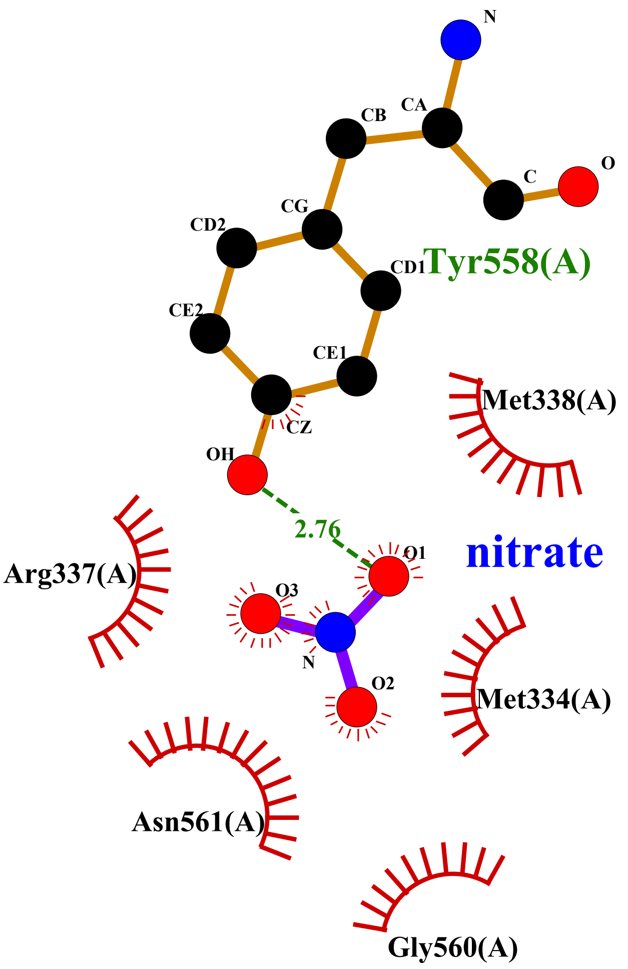
N
CA
CB
C
O
CG
CD2
CD1
Tyr558(A)
CE2
CE1
Met338(A)
CZ
OH
2.76
O1
nitrate
Arg337(A)
O3
N
O2
Met334(A)
Asn561(A)
Gly560(A)

Si9g29460.1
