# Supplementary material for: Genome-wide identification and characterization of the NPF genes provide new insight into low nitrogen tolerance in Setaria
Source: Front Plant Sci. 2022 Dec 14;13:1043832. doi: 10.3389/fpls.2022.1043832 (PMC9795848; doi:10.3389/fpls.2022.1043832)
Supplement: Supplementary Figure 1 — Chromosome location and distribution analysis of the SiNPF genes. Tandem duplicated genes are linked by a red curve. [file DataSheet_1.zip › Supplementary Data.zip!/Supplementary Data 3/Si9g29480.1.pdf]

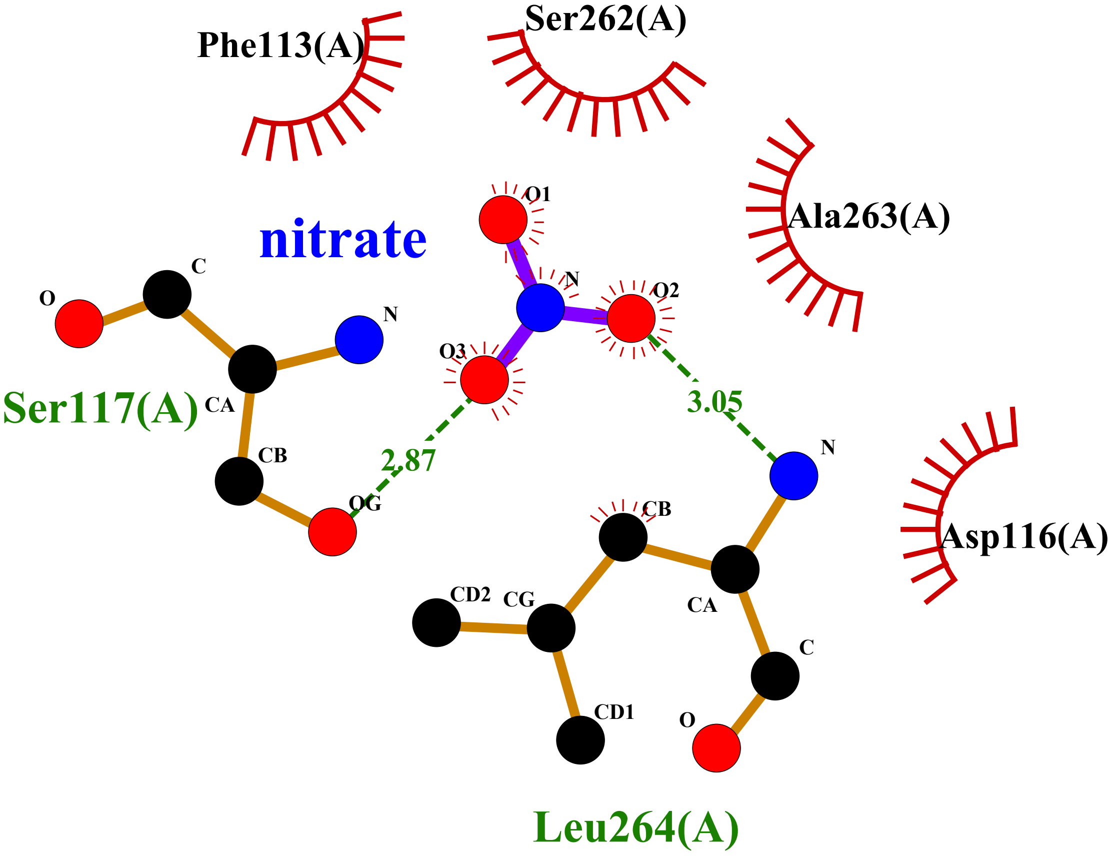
Phe113(A)
Ser262(A)
Ala263(A)
nitrate
O1
N
O2
O3
O
C
N
Ser117(A)
CA
CB
OG
2.87
3.05
N
Asp116(A)
CB
CD2
CG
CA
C
CD1
O
Leu264(A)

Si9g29480.1
