# Supplementary material for: Genome-wide identification and characterization of the NPF genes provide new insight into low nitrogen tolerance in Setaria
Source: Front Plant Sci. 2022 Dec 14;13:1043832. doi: 10.3389/fpls.2022.1043832 (PMC9795848; doi:10.3389/fpls.2022.1043832)
Supplement: Supplementary Figure 1 — Chromosome location and distribution analysis of the SiNPF genes. Tandem duplicated genes are linked by a red curve. [file DataSheet_1.zip › Supplementary Data.zip!/Supplementary Data 3/Si9g29490.1.pdf]

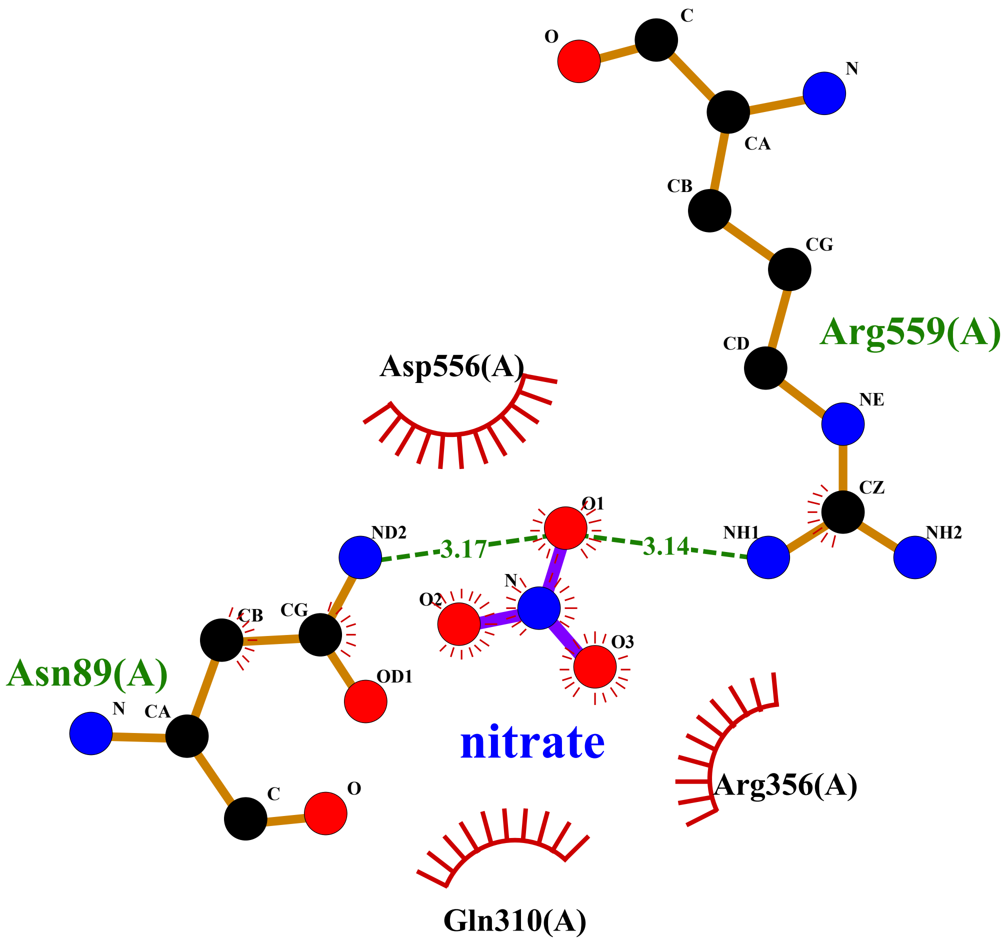

O
C
N
CA
CB
CG
Arg559(A)
CD
NE
Asp556(A)
CZ
O1
ND2
3.17
3.14
NH1
NH2
N
O2
CB
CG
O3
Asn89(A)
OD1
N
CA
nitrate
Arg356(A)
C
O
Gln310(A)

Si9g29490.1
