# Supplementary material for: Genome-wide identification and characterization of the NPF genes provide new insight into low nitrogen tolerance in Setaria
Source: Front Plant Sci. 2022 Dec 14;13:1043832. doi: 10.3389/fpls.2022.1043832 (PMC9795848; doi:10.3389/fpls.2022.1043832)
Supplement: Supplementary Figure 1 — Chromosome location and distribution analysis of the SiNPF genes. Tandem duplicated genes are linked by a red curve. [file DataSheet_1.zip › Supplementary Data.zip!/Supplementary Data 3/Si9g30230.1.pdf]

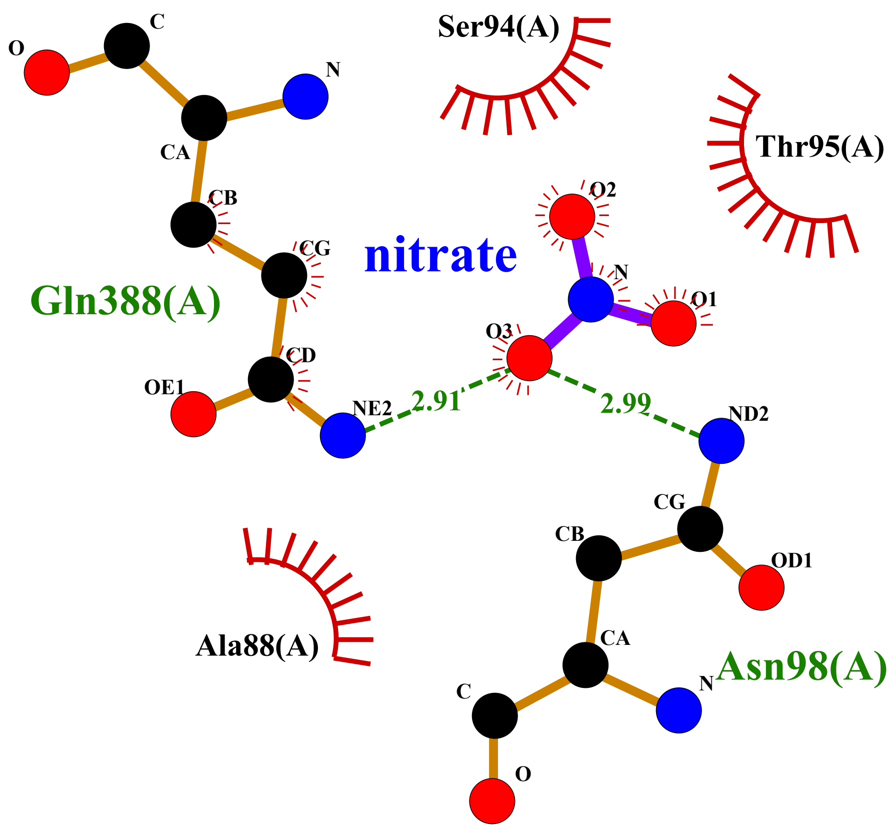
O
C
N
Ser94(A)
Thr95(A)
CA
CB
O2
CG
nitrate
N
Gln388(A)
O1
O3
CD
OE1
NE2
2.91
2.99
ND2
CG
CB
OD1
Ala88(A)
CA
Asn98(A)
N
C
O

Si9g30230.1
