# Supplementary material for: Genome-wide identification and characterization of the NPF genes provide new insight into low nitrogen tolerance in Setaria
Source: Front Plant Sci. 2022 Dec 14;13:1043832. doi: 10.3389/fpls.2022.1043832 (PMC9795848; doi:10.3389/fpls.2022.1043832)
Supplement: Supplementary Figure 1 — Chromosome location and distribution analysis of the SiNPF genes. Tandem duplicated genes are linked by a red curve. [file DataSheet_1.zip › Supplementary Data.zip!/Supplementary Data 3/Si9g30240.1.pdf]

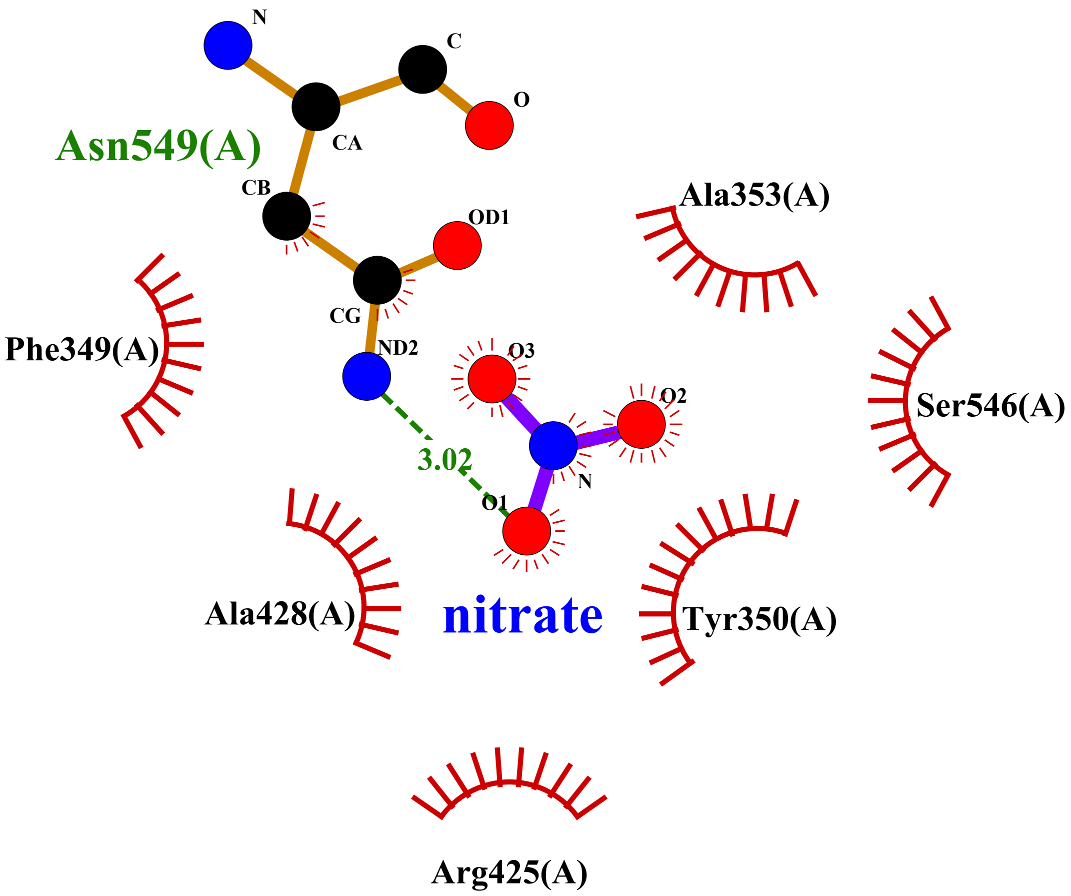

N
C
O
Asn549(A)
CA
CB
OD1
Ala353(A)
Phe349(A)
CG
ND2
O3
O2
Ser546(A)
3.02
N
O1
Ala428(A)
nitrate
Tyr350(A)
Arg425(A)

Si9g30240.1
