# Supplementary material for: Genome-wide identification and characterization of the NPF genes provide new insight into low nitrogen tolerance in Setaria
Source: Front Plant Sci. 2022 Dec 14;13:1043832. doi: 10.3389/fpls.2022.1043832 (PMC9795848; doi:10.3389/fpls.2022.1043832)
Supplement: Supplementary Figure 1 — Chromosome location and distribution analysis of the SiNPF genes. Tandem duplicated genes are linked by a red curve. [file DataSheet_1.zip › Supplementary Data.zip!/Supplementary Data 3/Si9g31060.1.pdf]

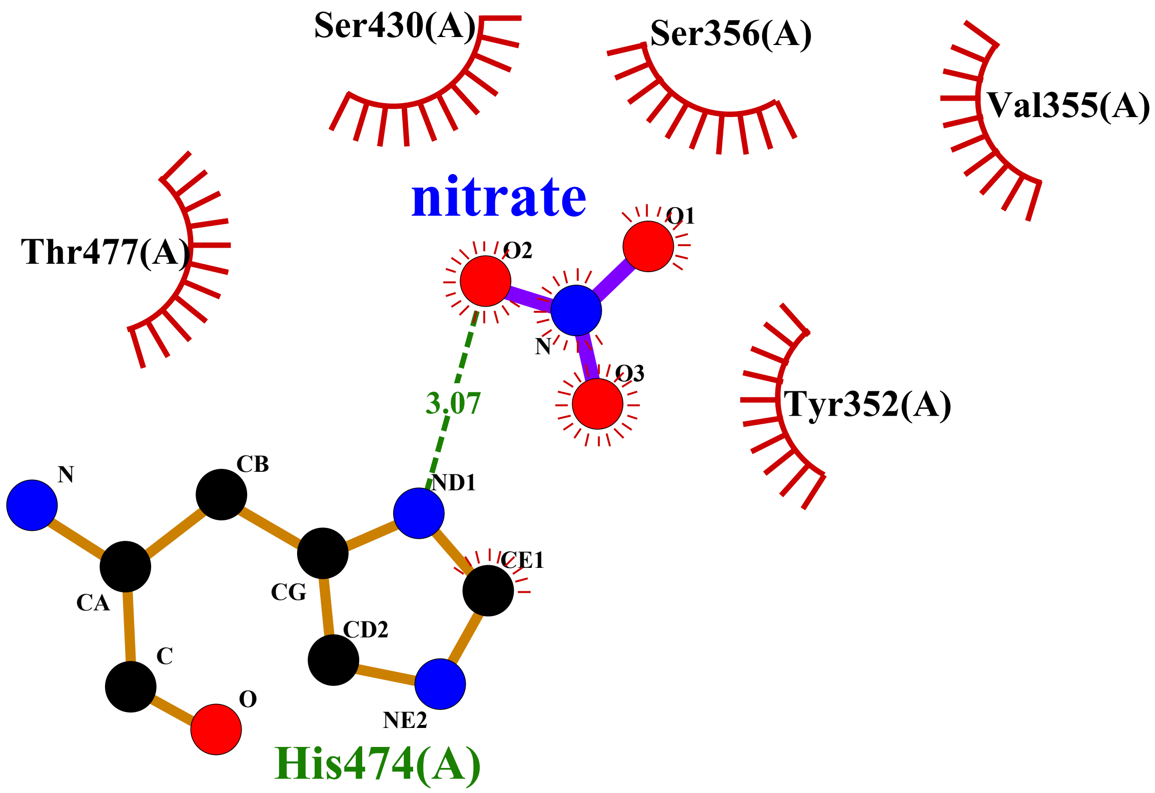
Ser430(A)
Ser356(A)
Val355(A)
nitrate
Thr477(A)
O1
O2
N
O3
Tyr352(A)
3.07
N
CB
ND1
CE1
CA
CG
CD2
C
O
NE2
His474(A)

Si9g31060.1
