# Supplementary material for: Genome-wide identification and characterization of the NPF genes provide new insight into low nitrogen tolerance in Setaria
Source: Front Plant Sci. 2022 Dec 14;13:1043832. doi: 10.3389/fpls.2022.1043832 (PMC9795848; doi:10.3389/fpls.2022.1043832)
Supplement: Supplementary Figure 1 — Chromosome location and distribution analysis of the SiNPF genes. Tandem duplicated genes are linked by a red curve. [file DataSheet_1.zip › Supplementary Data.zip!/Supplementary Data 3/Si9g46590.1.pdf]

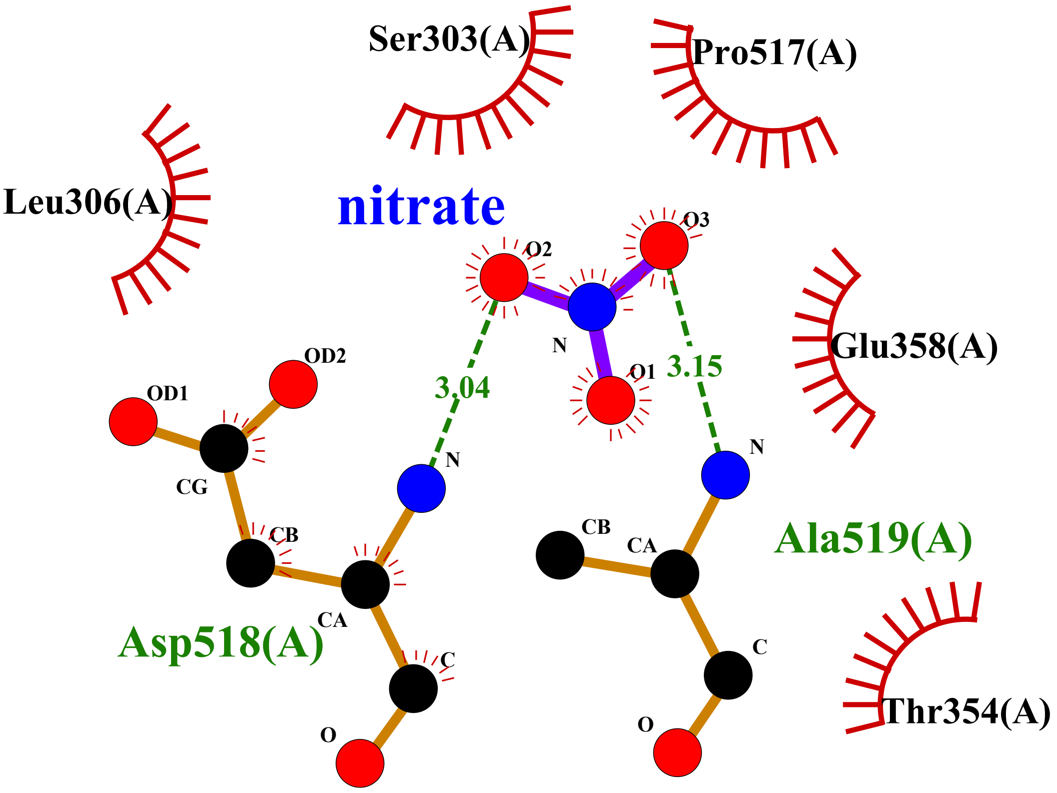

Ser303(A)
Pro517(A)
Leu306(A)
nitrate
O3
O2
N
O1
Glu358(A)
OD2
OD1
3.04
3.15
CG
N
N
CB
CB
CA
Ala519(A)
CA
Asp518(A)
C
C
Thr354(A)
O
O

Si9g46590.1
