# Supplementary material for: Genome-wide identification and characterization of the NPF genes provide new insight into low nitrogen tolerance in Setaria
Source: Front Plant Sci. 2022 Dec 14;13:1043832. doi: 10.3389/fpls.2022.1043832 (PMC9795848; doi:10.3389/fpls.2022.1043832)
Supplement: Supplementary Figure 1 — Chromosome location and distribution analysis of the SiNPF genes. Tandem duplicated genes are linked by a red curve. [file DataSheet_1.zip › Supplementary Data.zip!/Supplementary Data 3/Si9g46630.1.pdf]

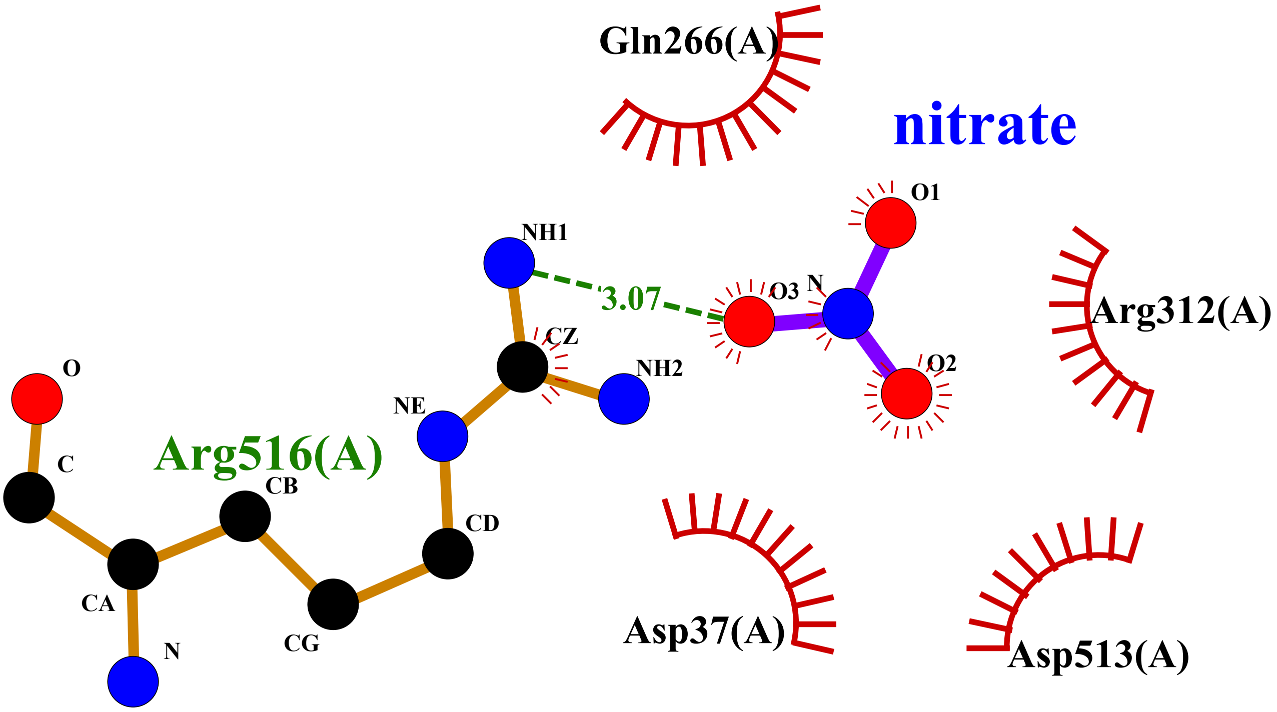
Gln266(A)
nitrate
O1
NH1
3.07
O3
N
Arg312(A)
CZ
NH2
O2
O
NE
Arg516(A)
C
CB
CD
CA
CG
N
Asp37(A)
Asp513(A)

Si9g46630.1
