# Supplementary material for: Genome-wide identification and characterization of the NPF genes provide new insight into low nitrogen tolerance in Setaria
Source: Front Plant Sci. 2022 Dec 14;13:1043832. doi: 10.3389/fpls.2022.1043832 (PMC9795848; doi:10.3389/fpls.2022.1043832)
Supplement: Supplementary Figure 1 — Chromosome location and distribution analysis of the SiNPF genes. Tandem duplicated genes are linked by a red curve. [file DataSheet_1.zip › Supplementary Data.zip!/Supplementary Data 3/Si9g46640.1.pdf]

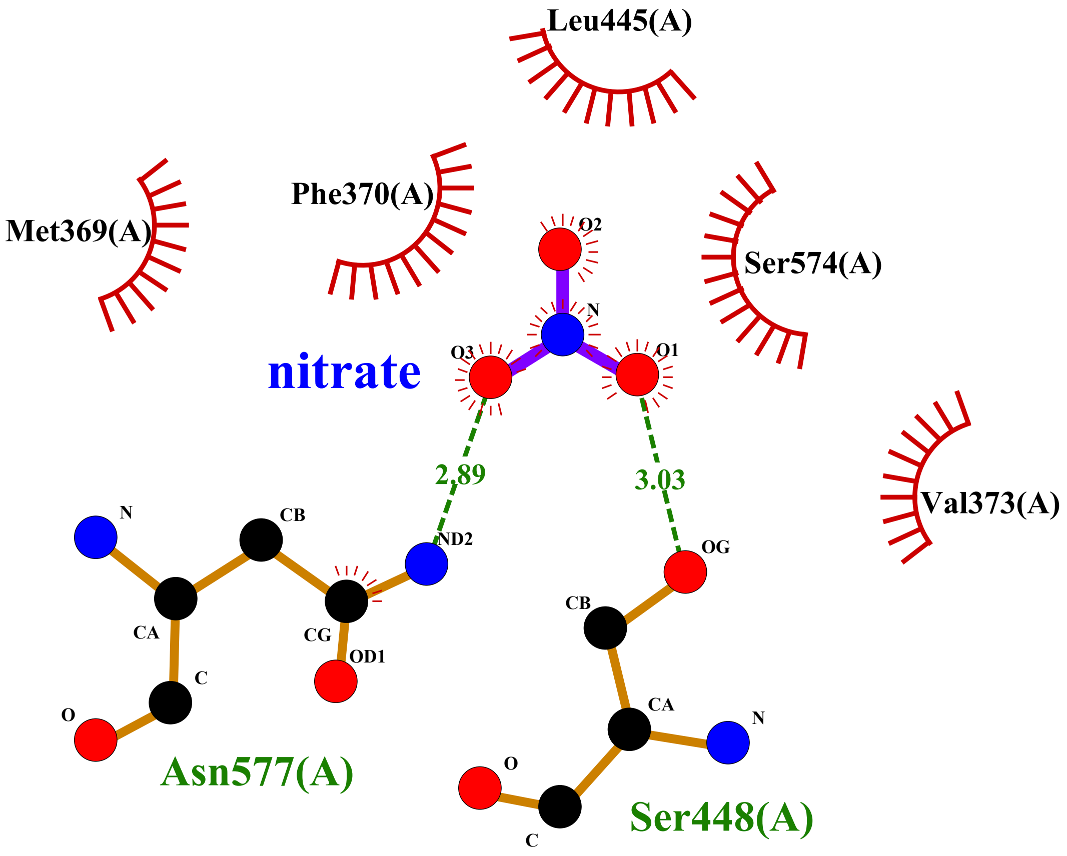
Leu445(A)
Met369(A)
Phe370(A)
O2
Ser574(A)
N
nitrate
O3
O1
2.89
3.03
Val373(A)
N
CB
ND2
OG
CA
CG
OD1
CB
C
O
CA
N
Asn577(A)
O
C
Ser448(A)

Si9g46640.1
