# Supplementary material for: Genome-wide identification and characterization of the NPF genes provide new insight into low nitrogen tolerance in Setaria
Source: Front Plant Sci. 2022 Dec 14;13:1043832. doi: 10.3389/fpls.2022.1043832 (PMC9795848; doi:10.3389/fpls.2022.1043832)
Supplement: Supplementary Figure 1 — Chromosome location and distribution analysis of the SiNPF genes. Tandem duplicated genes are linked by a red curve. [file DataSheet_1.zip › Supplementary Data.zip!/Supplementary Data 3/Si9g53060.1.pdf]

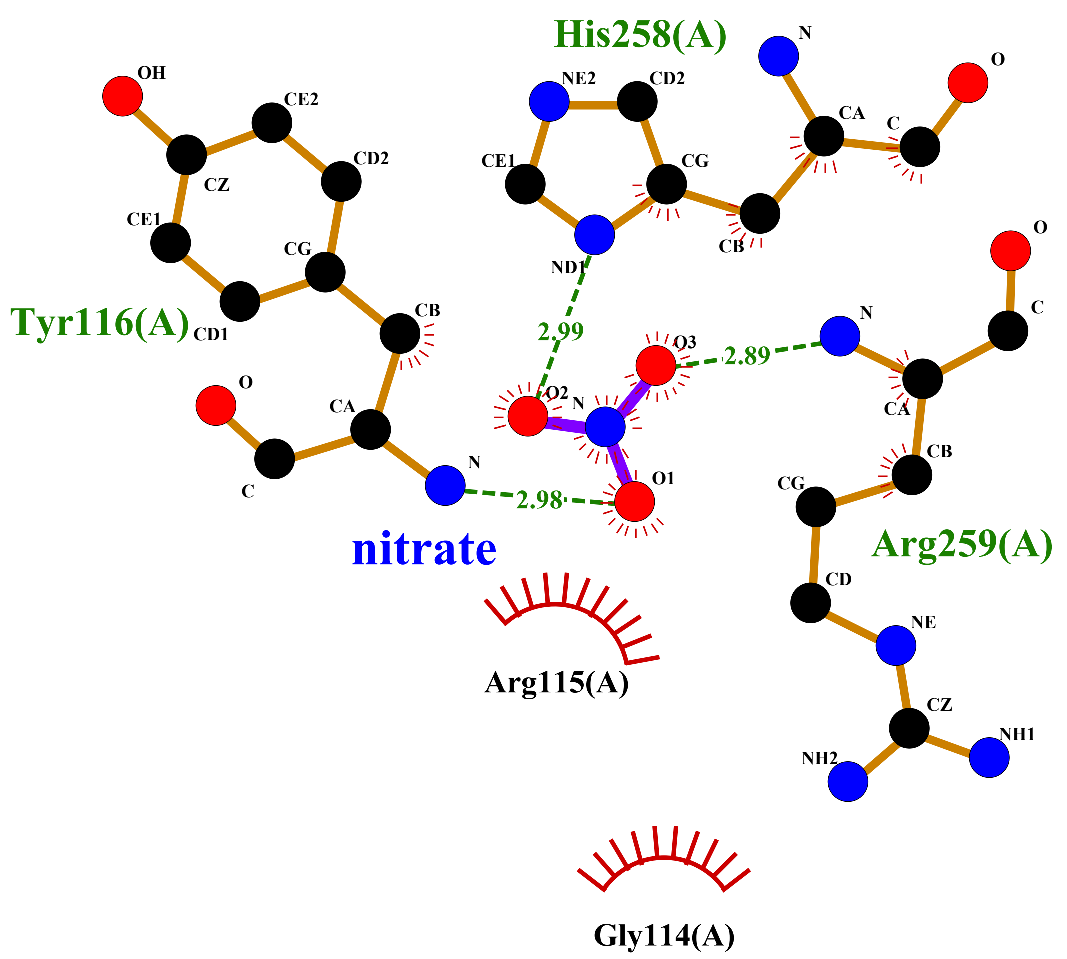

His258(A)
N
O
NE2
CD2
CA
C
CE1
CG
CB
OH
CE2
CD2
CZ
CE1
CG
ND1
O
C
Tyr116(A)
CD1
CB
2.99
O3
2.89
N
O
CA
O2
N
CA
CA
CB
N
CG
C
2.98
O1
Arg259(A)
nitrate
CD
NE
Arg115(A)
CZ
NH1
NH2
Gly114(A)

Si9g53060.1
