# Supplementary material for: Genome-wide identification and characterization of the NPF genes provide new insight into low nitrogen tolerance in Setaria
Source: Front Plant Sci. 2022 Dec 14;13:1043832. doi: 10.3389/fpls.2022.1043832 (PMC9795848; doi:10.3389/fpls.2022.1043832)
Supplement: Supplementary Figure 1 — Chromosome location and distribution analysis of the SiNPF genes. Tandem duplicated genes are linked by a red curve. [file DataSheet_1.zip › Supplementary Data.zip!/Supplementary Data 3/Si9g56050.1.pdf]

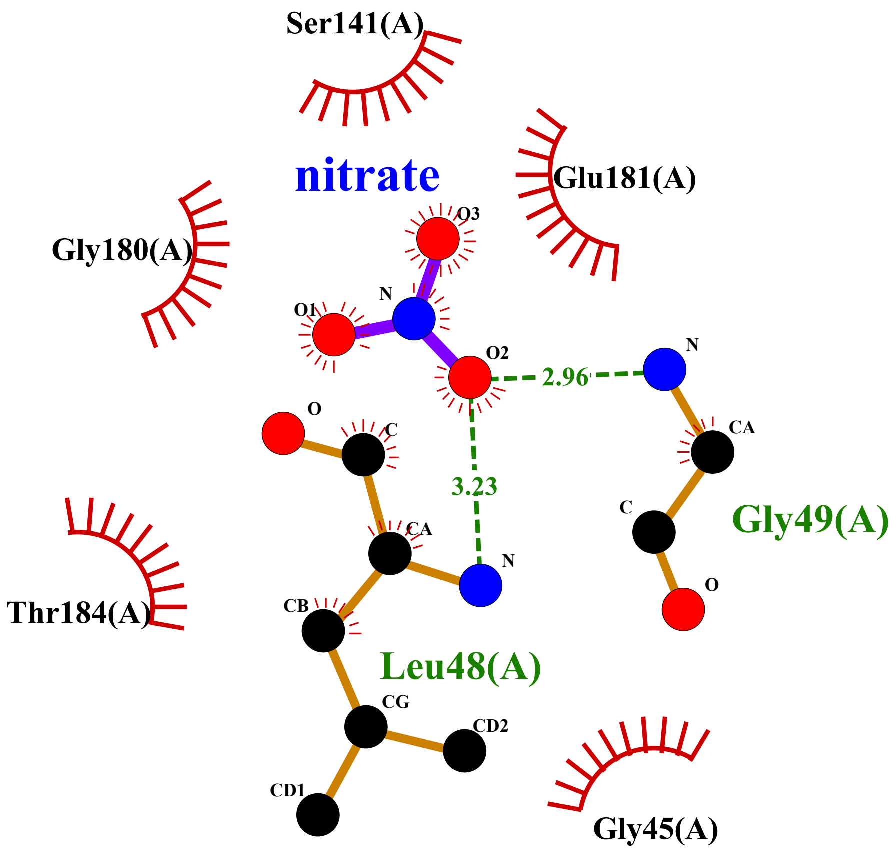
Ser141(A)
nitrate
Glu181(A)
Gly180(A)
O3
N
O1
O2
2.96
N
O
C
CA
3.23
C
Gly49(A)
CA
N
O
Thr184(A)
CB
Leu48(A)
CG
CD2
CD1
Gly45(A)

Si9g56050.1
