# Supplementary material for: Genome-wide identification and characterization of the NPF genes provide new insight into low nitrogen tolerance in Setaria
Source: Front Plant Sci. 2022 Dec 14;13:1043832. doi: 10.3389/fpls.2022.1043832 (PMC9795848; doi:10.3389/fpls.2022.1043832)
Supplement: Supplementary Figure 1 — Chromosome location and distribution analysis of the SiNPF genes. Tandem duplicated genes are linked by a red curve. [file DataSheet_1.zip › Supplementary Data.zip!/Supplementary Data 3/Si9g56350.1.pdf]

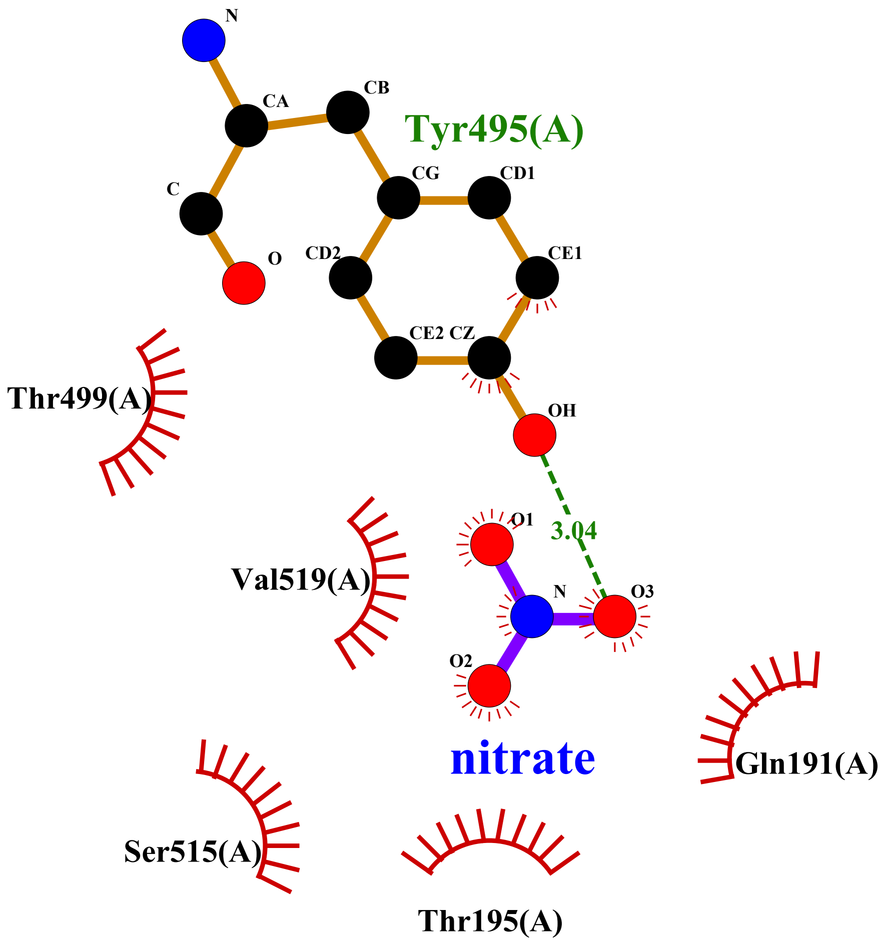
N
CA
CB
Tyr495(A)
C
CG
CD1
O
CD2
CE1
CE2 CZ
Thr499(A)
OH
O1
3.04
Val519(A)
N
O3
O2
nitrate
Gln191(A)
Ser515(A)
Thr195(A)

Si9g56350.1
